# Organoarsonate- and Dimethylarsinate-Functionalized Hexamolybdates(V): A Multifaceted Study on Synthesis, Structural Dynamics, and Antibacterial Properties

**DOI:** 10.1021/acs.inorgchem.5c02852

**Published:** 2025-08-25

**Authors:** Vinaya Siby, Arun Pal, Anupam Sarkar, Bassem S. Bassil, Anneke Immoor, James Ziemah, Jana Hölscher, Levente Kiss, Cristian Silvestru, Matthias S. Ullrich, Nikolai Kuhnert, Ulrich Kortz

**Affiliations:** a School of Science, Constructor University, Campus Ring 1, 28759 Bremen, Germany; b Department of Chemistry, Supramolecular Organic and Organometallic Chemistry Centre (SOOMCC), Faculty of Chemistry and Chemical Engineering, Babeş-Bolyai University, 11 Arany Janos, 400028 Cluj-Napoca, Romania

## Abstract

We report on the synthesis, functionalization, and structural characterization of 11 novel dimethylarsinate-functionalized arsenomolybdates­(V), [RAsMo^V^_6_O_15_(OH)_3_{AsO_2_(CH_3_)_2_}_3_]^2–^ (R = HO, CH_3_, C_2_H_5_, C_6_H_5_, 3,5-(HOOC)_2_C_6_H_3_, 4-FC_6_H_4_, 4-F_3_CC_6_H_4_, 4-F_3_COC_6_H_4_, 4-BrC_6_H_4_, and 4-N_3_C_6_H_4_) and [As^III^Mo^V^_6_­O_15_(OH)_3_{AsO_2_(CH_3_)_2_}_3_]^3–^, featuring a reduced hexanuclear {Mo^V^_6_O_24_} core, peripherally coordinated by three dimethylarsinate ligands and centrally functionalized with diverse organoarsonate, arsenate, or arsenite groups, including carboxylated, fluorinated, brominated, and azido derivatives. Synthesized via a simple one-pot aqueous method, the compounds were thoroughly characterized by single-crystal X-ray diffraction, thermogravimetric analysis, and elemental analysis in the solid state. Solution-phase stability was assessed by multinuclear (^1^H, ^13^C, and ^19^F) nuclear magnetic resonance, while gas-phase behavior and fragmentation pathways were probed through electrospray ionization mass spectrometry, and tandem mass spectrometry (collision-induced dissociation). Antibacterial screening against *Escherichia coli, Bacillus subtilis*, and three pathogenic bacterial strains *Listeria monocytogenes, Salmonella enterica*, and *Vibrio parahaemolyticus* revealed that three of the 11 polyanions exhibit moderate antibacterial activity. Additionally, the synthesis of 3,5-bis­(carboxy)­phenylarsonic acid, 3,5-(HOOC)_2_C_6_H_3_AsO_3_H_2_, is reported here for the first time.

## Graphical abstract

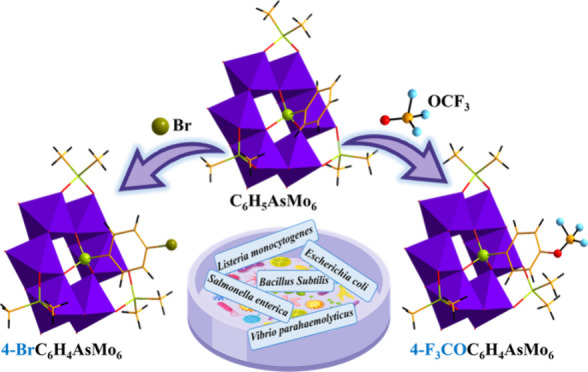

## Introduction

Polyoxometalates (POMs) are a class of discrete, anionic polynuclear metal oxide clusters composed of early transition metals in high oxidation states (M = W^VI^, Mo^VI^, V^V^, Nb^V^, and Ta^V^),^1^ known for their broad applications in photo/electrocatalysis, medicine, magnetism, and energy storage/conversion.^2^ POMs offer significant potential for structural and functional modification, as the covalent integration of organic or organometallic moieties into their frameworks enables precise control over molecular properties such as shape, size, solubility, lipophilicity, stability, toxicity, redox, and acid–base behavior, thereby facilitating the rational design of advanced inorganic–organic hybrid materials with tailored functionalities for diverse applications.^3^

Building on this structural versatility, we recently reported a series of reduced dimethylarsinate-functionalized, hexanuclear phosphomolybdenum clusters with the general formula [RPMo^V^_6_O_15_(OH)_3_{AsO_2_(CH_3_)_2_}_3_]^2–^ (R = H, HO, CH_3_, HO_2_CCH_2_, HO_2_CC_2_H_4_, C_6_H_5_, 4-FC_6_H_4_, and 4-F_3_COC_6_H_4_), characterized by a cyclic {Mo_6_^V^O_24_} core, flanked by dimethylarsinate ligands and central organophosphonate substitutions.^4^ This system was extensively studied in solid, solution, and gas phases, with ESI-MS confirming the coexistence of monomeric and dimeric species. Ion mobility mass spectrometry further enabled the separation of both entities, allowing for the acquisition of clean spectra for each. Haushalter and Lai originally isolated this hexanuclear molybdic wheel archetype as the reduced molybdenum phosphate, {P_4_Mo^V^_6_}, where peripheral phosphates and hydroxo bridges connect {Mo^V^_2_O_4_} dimers.^5^ Subsequent structural analogues, such as {(C_6_H_5_P)_4_Mo^V^_6_},^6a–6c^ {(C_6_H_5_As)_4_Mo^V^_6_},^6d^ and thiomolybdate variants {X_4_Mo^V^_6_S_6_O_6_} (X = HPO_4_ or HAsO_4_),^7^ have further highlighted the framework’s adaptability. Additionally, reactions of reduced Mo^V^ systems with methylenebisphosphonates and various structure-directing agents yielded cyclic compounds, {(Mo^V^_2_O_4_)­(O_3_PCH_2_PO_3_)}_*n*_ (*n* = 3, 4, or 10}, encapsulating additional ligands such as MoO_4_^2–^, SO_3_^2–^, CO_3_^2–^, O_3_PCH_2_PO_3_^4–^, and CH_3_AsO_3_^2–^.^8^

Organoarsenic chemistry, although it has progressed more slowly than organophosphorus chemistry,^9^ boasts a rich history, dating back to 1760, when Cadet de Gassicourt synthesized cacodyl ((CH_3_)_2_AsAs­(CH_3_)_2_), the first known organometallic compound.^10^ This slower development, partly due to toxicity concerns, contrasts with the rapid progress in organophosphorus chemistry, driven by ^31^P NMR spectroscopy as well as its well-documented reactivity and electronic characteristics. In the realm of POM chemistry, initial pivotal contributions to organoarsonate-functionalized POMs were made by Pope’s group, which reported tetramolybdobisarsonate complexes, [R_2_AsMo_4_O_15_H]^2–^ (R = CH_3_, C_2_H_5_, or C_6_H_5_),^11^ a system initially proposed by Rosenheim and Bilecki,^12^ and cyclic, hexamolybdobisarsonate complexes, [(RAs)_2_Mo_6_O_24_]^4–^ (R = CH_3_, C_6_H_5_, or *p*-C_6_H_4_NH_2_).^13^ Further advancements involved Matsumoto’s synthesis of [(C_6_H_5_As)_2_Mo_6_O_24_(H_2_O)]^4–^ and Liu et al.’s contributions with the pentamolybdate [(*n*-C_3_H_7_As)_2_Mo_5_O_21_]^4–^ and hexamolybdate [(*n*-C_3_H_7_As)_2_Mo_6_O_24_]^4–^ frameworks.^14^ Moreover, Kortz’s group expanded this family of organoarsonate-containing hexamolybdates by integrating *para*-substituted phenyl derivatives (R = 4-BrC_6_H_4_, 4-N_3_C_6_H_4_, 4-FC_6_H_4_, 4-F_3_CC_6_H_4_, or 4-F_3_COC_6_H_4_).^15^ Beyond these, dual-site functionalization has also been realized. In one such example, a hexamolybdic wheel is centrally occupied by lone pair-containing heteroatoms and surrounded peripherally by amino acids bound via their carboxylate groups as in [As^III^Mo_6_O_21_(O_2_CRNH_3_)_3_]^3–^ (R = CH_2_, C_2_H_4_, or C_3_H_6_).^16^

POMs are recognized as potent metallodrugs with promising antimicrobial, antiviral, and antitumor properties.^17^ In particular, hybrid POM systems, formed by integrating electron-rich POM cores into bioactive frameworks, have emerged as strong candidates for biomedical research.^18^ With antibiotic resistance becoming a global health crisis, the development of novel antimicrobial agents is of paramount importance.^19^ Halogenated compounds, especially fluoro-pharmaceuticals, are widely employed in contemporary medicinal chemistry; thus, the strategic introduction of biologically active halogen substituents, such as fluoro or bromo, at the *para* position of phenylarsonate rings in polyoxomolybdates represents a promising approach.^20^ Such hybrid frameworks not only enhance stability under physiological conditions but also offer tailored bioactivity via modulation of their organic constituents. Notably, Kortz’s group has shown previously that {PhSb^III^}-incorporated polyoxotungstates exhibit enhanced antibacterial and antitumor effects, underlining the therapeutic potential of functionalized POMs.^21^

Expanding on our prior work with reduced molybdate systems in the presence of cacodylate and phosphonates under aqueous, reducing conditions, we now report the substitution of phosphonates with arsonates. This modification yielded a series of reduced, dimethylarsinate-functionalized arsenomolybdates­(V), [RAsMo^V^_6_O_15_(OH)_3_{AsO_2_(CH_3_)_2_}_3_]^2–^ and [As^III^Mo^V^_6_O_15_(OH)_3_{AsO_2_(CH_3_)_2_}_3_]^3–^. These structures incorporate a variety of organoarsonates (RAsO_3_^2–^), arsenate, and arsenite as structure-directing agents. Given the increasing utility of ESI-MS for characterizing the intricate structures of complex inorganic POM clusters, with numerous gas-phase fragmentation studies of POMs being investigated using CID MS/MS,^22^ we undertook a detailed mass spectrometric analysis to elucidate the fragmentation behavior of these novel arsenomolybdate species. Furthermore, recognizing both their structural novelty and the rising interest in bioactive POMs, we carried out preliminary studies to assess the biological activity of these arsenomolybdates.

## Experimental Section

### Materials and Physical Measurements

The precursors ethylarsonic acid, C_2_H_5_AsO_3_H_2_,^23^ (4-fluorophenyl)­arsonic acid, (4-FC_6_H_4_)­AsO_3_H_2_,^24^ (4-trifluoromethylphenyl)­arsonic acid, (4-F_3_CC_6_H_4_)­AsO_3_H_2_,^15b^ (4-trifluoromethoxyphenyl)­arsonic acid, (4-F_3_COC_6_H_4_)­AsO_3_H_2_,^15b^ (4-bromophenyl)­arsonic acid, (4-BrC_6_H_4_)­AsO_3_H_2_,^15a^ and (4-azidophenyl)­arsonic acid, (4-N_3_C_6_H_4_)­AsO_3_H_2_,^25^ were synthesized following published literature procedures and characterized using infrared (IR) and nuclear magnetic resonance (NMR) spectroscopy. The precursor 3,5-bis­(carboxy)­phenylarsonic acid, (3,5-(HOOC)_2_C_6_H_3_AsO_3_H_2_), is reported and characterized here for the first time. All other reagents were purchased from commercial sources and used as received without further purification.

The elemental analyses were performed by Zentrallabor, Technische Universität Hamburg (TUHH), Am Schwarzenberg-Campus 1, 21073 Hamburg (Na, Mo, and As), and Analytische Laboratorien, Industriepark Kaiserau (Haus Heidbruch), 51789 Lindlar, Germany (C, H, N, F, and Br). The sodium content was further verified in-house by atomic absorption (AA) spectroscopy on a Varian SpectrAA 220 AA spectrometer. The FT-IR spectra were recorded on KBr pellets using a Nicolet Avatar 370 spectrophotometer operating in the 400–4000 cm^–1^ range with 32 scans and a 4 cm^–1^ resolution. The peak intensities are abbreviated as follows: w, weak; m, medium; s, strong; sh, shoulder. The crystal water content was analyzed via thermogravimetric analysis (TGA) on a TA Instrument SDT Q600 ramped from room temperature to 600 °C at a heating rate of 5 °C min^–1^ under a N_2_ flow of 10 mL min^–1^. Multinuclear solution NMR spectra (^1^H, ^13^C­{H}, and ^19^F) were recorded at room temperature on a JEOL ECS 400 MHz spectrometer using 5 mm probes, tuned to respective resonance frequencies of 399.78 MHz (^1^H), 100.52 MHz (^13^C­{H}), and 376.17 MHz (^19^F). The chemical shifts were referenced to tetramethylsilane (^1^H and ^13^C­{H}) and CFCl_3_ (^19^F). Acquisition of the ^13^C­{H} NMR spectra for the polyanions required an overnight run. For assignments of the ^1^H and ^13^C NMR resonances, see numbering schemes in the SI. Ultraviolet–visible (UV–vis) studies were conducted using a Varian Cary 100 Bio UV–vis spectrophotometer over the 200–800 nm range, using 1 cm quartz cuvettes.

High-resolution mass spectra were recorded using a Bruker Daltonics QTOF Impact HD mass spectrometer employing both negative and positive electrospray ionization modes. The QTOF Impact mass spectrometer (Bruker Daltonics) was fitted with an ESI source, and external calibration was achieved with 10 mL of 0.1 M sodium formate solution. The instrument ion source and tubing were rinsed with methanol. Calibration was carried out using the enhanced quadratic calibration mode. All MS measurements were performed in both negative and positive ion modes. Samples were measured as direct infusions at a concentration of 10 μg/mL in deionized water at a flow rate of 180 μL/min. Samples were prepared by dissolving 1 mg of POM in 1 mL of deionized water followed by a 1:100 dilution. Spectral simulations were carried out in Data Analysis 4.1 (Bruker Daltonics, Bremen). For tandem MS measurements, POM precursor ions were isolated with an isolation window of 20 Da and fragmented by CID if necessary, with an increase in collision energy until the disappearance of the precursor ion.

### Synthetic Procedures

#### Synthesis of 3,5-Bis­(carboxy)­phenylarsonic Acid, 3,5-(HOOC)_2_C_6_H_3_AsO_3_H_2_

Dimethyl 5-aminoisophtalate (4.18 g, 20.0 mmol) was dissolved in 22 mL of H_2_O, acidified with 5 mL of conc. HCl, and cooled to 0 °C in an ice bath. After stirring for 15 min, a cold aqueous solution of NaNO_2_ (1.45 g, 21.0 mmol, in 5 mL H_2_O) was added dropwise. Separately, a mixture of As_2_O_3_ (2.50 g, 12.6 mmol) and Na_2_CO_3_ (5.30 g, 33.1 mmol) was dissolved in 25 mL of hot water and stirred for 20 min, followed by the addition of CuSO_4_·5H_2_O (0.11 g, 21.7 mmol). The solution was heated until boiling for another 15 min. Upon cooling, 5 mL of diethyl ether was added to form an organic layer on the liquid. Then, the former solution was added dropwise under stirring for 1 h in an ice bath. The resulting mixture was then filtered and acidified to pH 6, filtered again, and finally acidified to pH 1 before a final filtration. The volume of the solution was reduced to one-third, and the mixture was refrigerated to induce precipitation. The resulting off-white solid, 3,5-bis­(methoxycarbonyl)­phenylarsonic acid, was washed with diluted HCl and collected for further analysis. Yield: 1.2 g (19%). FT-IR (KBr pellet, ν/cm^–1^): 3444 (m), 2957 (m), 2336 (m), 1736 (s), 1444 (m), 1277 (s), 1144 (w), 1112 (w), 998 (m), 916 (s), 849 (w), 803 (m), 751 (m), 671 (w), 495 (w) (Figure S1). ^1^H NMR (DMSO-*d*_6_, ppm): δ 3.9 (s, 6H, C*H*_3_), 8.4 (s, 2H, *ortho*-C_6_ *H*_3_), 8.6 (s, 1H, *para*-C_6_ *H*_3_). ^13^C­{^1^H} NMR (DMSO-*d*_6_, ppm): δ 54.0 (*C*H_3_), 132.1 (*C*-2), 134.0 (*C*-1), 135.4 (*C*-3), 136.8 (*C*-4), 165.4 (*C*OOCH_3_) (Figure S2).

3,5-Bis­(methoxycarbonyl)­phenylarsonic acid (1.2 g) was dissolved in 14 mL of water, followed by the addition of 7 mL of concentrated HCl. The solution was refluxed for 24 h at 105 °C. After cooling, the solvent was evaporated under vacuum, and the resulting white precipitate of 3,5-bis­(carboxy)­phenylarsonic acid, 3,5-(HOOC)_2_C_6_H_3_AsO_3_H_2_, was air-dried for 1 day. Yield: 0.7 g (64%). FT-IR (KBr pellet, ν/cm^–1^): 3460 (m), 3174 (m), 3097 (m), 2325 (m), 1881 (m), 1725 (s), 1668 (m), 1603 (m), 1453 (w), 1322 (m), 1293 (s), 1238 (s), 1194 (s), 1161 (s), 1118 (s), 994 (w), 881 (s), 821 (m), 799 (s), 776 (m), 712 (w), 668 (s), 518 (w), 486 (w), 433 (w) (Figure S1). ^1^H NMR (DMSO-*d*_6_, ppm): δ 8.4 (s, 2H, *ortho*-C_6_ *H*_3_), 8.6 (s, 1H, *para*-C_6_ *H*_3_). ^13^C­{^1^H} NMR (DMSO-*d*_6_, ppm): δ 132.9 (*C*-2), 134.1 (*C*-1), 134.9 (*C*-3), 135.8 (*C*-4), 166.0 (*C*OOH) (Figure S3).

#### Synthesis of Na_2.5_(NH_4_)_0.5_[As^III^Mo^V^_6_O_15_(OH)_3_{AsO_2_(CH_3_)_2_}_3_]·11H_2_O (**NaNH_4_–As^III^Mo_6_**)

Na_2_MoO_4_·2H_2_O (0.024 g, 0.10 mmol), N_2_H_4_·2HCl (0.011 g, 0.10 mmol), and arsenic trioxide, As_2_O_3_ (0.01 g, 0.05 mmol) were dissolved in 2 mL of 1 M sodium dimethylarsinate buffer (1 M aqueous solution of dimethylarsinic acid (pH 4) adjusted to pH 7 by adding NaOH pellets). The resulting mixture was stirred and heated at 80 °C for 1 h in a sealed vial. Dark-red, hexagonal crystals (Figure S4a) gradually formed over 2 weeks as the reaction mixture cooled. The crystals were then filtered and air-dried. Isolated yield: 0.017 g (63% based on Mo). Elemental analysis (%) calcd (found) for **NaNH_4_–As^III^Mo_6_**: Na, 3.55 (3.85); Mo, 35.6 (35.8); As, 18.5 (19.3); C, 4.46 (4.49); H, 2.80 (3.27); N, 0.43 (0.62). FT-IR (2% KBr pellet, ν/cm^–1^): 3430 (s) [ν­(O–H)], 2997 (w), 2927 (w) [ν­(C–H)], 1643 (s) [δ­(O–H)], 1410 (m) [δ­(C–H)], 1276 (m) [ν­(As–C)], 1032 (m), 938 (s) [ν­(Mo=O)], 856 (s) [ν­(As–O)], 753 (s), 622 (s), 493 (s), 428 (s) [δ­(Mo–O­(Mo))] (Figure S5a). ^1^H NMR (H_2_O/D_2_O, ppm): δ 1.6 (s, 3H, (C*H*_3_)_2_AsO_2_), 2.0 (s, 3H, (C*H*_3_)_2_AsO_2_) (Figure 2a). ^13^C­{^1^H} NMR (H_2_O/D_2_O, ppm): δ 17.9 ((*C*H_3_)_2_AsO_2_), 19.4 ((*C*H_3_)_2_AsO_2_) (Figure 2b).

#### Synthesis of Na_1.5_(NH_4_)_0.5_[HOAsMo^V^_6_O_15_(OH)_3_{AsO_2_(CH_3_)_2_}_3_]·0.6NH_4_Cl·10H_2_O (**NaNH_4_–As^V^Mo_6_**)

(NH_4_)_6_Mo_7_O_24_·4H_2_O (0.265 g, 0.215 mmol) and N_2_H_4_·2HCl (0.052 g, 0.500 mmol) were completely dissolved in 10 mL of 0.5 M sodium dimethylarsinate buffer (pH 7). Following this, disodium hydrogen arsenate heptahydrate, Na_2_HAsO_4_·7H_2_O (0.078 g, 0.250 mmol), was added to the mixture and the pH was gradually adjusted to 6.8–6.9 by the dropwise addition of 6 M NaOH solution. The resulting dark-red solution was heated at 80 °C for 1 h. After 3 weeks of cooling at room temperature, dark-red, block-shaped crystals (Figure S4b) formed, which were collected and air-dried. Isolated yield: 0.06 g (15% based on Mo). Elemental analysis (%) calcd (found) for **NaNH_4_–As^V^Mo_6_**: Na, 2.12 (2.46); Mo, 35.4 (34.9); As, 18.4 (18.0); C, 4.43 (4.48); H, 2.88 (2.95); N, 0.95 (1.19). FT-IR (2% KBr pellet, ν/cm^–1^): 3397 (s), 3220 (s) [ν­(N–H), ν­(O–H)], 3014 (w), 2924 (w) [ν­(C–H)], 1630 (m) [δ­(O–H)], 1402 (m) [δ­(C–H)], 1271 (w) [ν­(As–C)], 966 (s), 921 (w) [ν­(Mo=O)], 822 (s) [ν­(As–O)], 746 (m), 654 (w), 527 (w), 494 (m), 457 (m) [δ­(Mo–O­(Mo))] (Figure S5b). ^1^H NMR (H_2_O/D_2_O, ppm): δ 1.9 (s, 3H, (C*H*_3_)_2_AsO_2_), 2.2 (s, 3H, (C*H*_3_)_2_AsO_2_) (Figure 2a). ^13^C­{^1^H} NMR (H_2_O/D_2_O, ppm): δ 18.7 ((*C*H_3_)_2_AsO_2_), 18.9 ((*C*H_3_)_2_AsO_2_) (Figure 2b).

#### Synthesis of NaNH_4_[CH_3_AsMo^V^_6_O_15_(OH)_3_{AsO_2_(CH_3_)_2_}_3_]·10H_2_O (**NaNH_4_–CH_3_AsMo_6_**)

(NH_4_)_6_Mo_7_O_24_·4H_2_O (0.053 g, 0.04 mmol), N_2_H_4_·2HCl (0.011 g, 0.10 mmol), and disodium methylarsonate, CH_3_AsO_3_Na_2_ (0.009 g, 0.05 mmol), were added to 2 mL of 0.5 M sodium dimethylarsinate buffer (pH 7). The resulting mixture was stirred and heated at 80 °C for 1 h. Dark-red, block-shaped crystals started appearing after 1 day as the solution cooled to room temperature. Isolated yield: 0.030 g (40% based on Mo). Elemental analysis (%) calcd (found) for **NaNH_4_–CH_3_AsMo_6_**: Na, 1.45 (1.22); Mo, 36.2 (35.5); As, 18.9 (19.1); C, 5.29 (5.41); H, 3.05 (3.17); N, 0.88 (1.07). FT-IR (2% KBr pellet, ν/cm^–1^): 3416 (s), 3202 (s) [ν­(N–H), ν­(O–H)], 3017 (w), 2925 (w) [ν­(C–H)], 1625 (s) [δ­(O–H)], 1477 (w), 1442 (w), 1403 (m) [δ­(C–H)], 1275 (w) [ν­(As–C)], 968 (s), 936 (w) [ν­(Mo=O)], 822 (s) [ν­(As–O)], 748 (m), 656 (w), 521 (w), 493 (m), 459 (m) [δ­(Mo–O­(Mo))] (Figure S5c). ^1^H NMR (H_2_O/D_2_O, ppm): δ 0.96 (s, 3H, C*H*_3_AsO_3_), 1.9 (s, 9H, (C*H*_3_)_2_AsO_2_), and 2.2 (s, 9H, (C*H*_3_)_2_AsO_2_) (Figure S20a). ^13^C­{^1^H} NMR (H_2_O/D_2_O, ppm): δ 18.0 ((*C*H_3_)_2_AsO_2_), 18.9 (*C*H_3_AsO_3_), 19.7 ((*C*H_3_)_2_AsO_2_) (Figure S20b).

#### Synthesis of Na_1.5_(NH_4_)_0.5_[C_2_H_5_AsMo^V^_6_O_15_(OH)_3_{AsO_2_(CH_3_)_2_}_3_]·10H_2_O (**NaNH_4_–C_2_H_5_AsMo_6_**)

The polyanion **C_2_H_5_AsMo_6_** was synthesized following the same procedure as for **As^III^Mo_6_**, except that ethylarsonic acid, C_2_H_5_AsO_3_H_2_ (0.008 g, 0.05 mmol), was used instead of As_2_O_3_. The reaction mixture (pH 7) was heated at 80 °C for 1 h in a sealed vial. Upon cooling to room temperature, red crystals (Figure S4c) began forming within a day. Isolated yield: 0.015 g (56% based on Mo). Elemental analysis (%) calcd (found) for **NaNH_4_–C_2_H_5_AsMo_6_**: Na, 2.2 (2.4); Mo, 35.9 (36.3); As, 18.7 (19.8); C, 5.99 (6.16); H, 3.01 (3.30); N, 0.44 (0.49). FT-IR (2% KBr pellet, ν/cm^–1^): 3401 (s) [ν­(O–H)], 3014 (w), 2926­(w) [ν­(C–H)], 1636 (m) [δ­(O–H)], 1456 (w), 1406 (m) [δ­(C–H)], 1274 (m) [ν­(As–C)], 968 (s), 918 (w) [ν­(Mo=O)], 824 (s) [ν­(As–O)], 747 (m), 655 (w), 615 (w), 493 (m), 459 (m) [δ­(Mo–O­(Mo))] (Figure S5d). ^1^H NMR (H_2_O/D_2_O, ppm): δ 0.57 (t, 3H, ^3^ *J*_HH_ = 8 Hz, C*H*_3_CH_2_AsO_3_), 1.21 (q, 2H, ^3^ *J*_HH_ = 8 Hz, CH_3_C*H*_2_AsO_3_), 1.9 (s, 9H, (C*H*_3_)_2_AsO_2_), 2.3 (s, 9H, (C*H*_3_)_2_AsO_2_) (Figure S22).

#### Synthesis of NaNH_4_[C_6_H_5_AsMo^V^_6_O_15_(OH)_3_{AsO_2_(CH_3_)_2_}_3_]·10H_2_O (**NaNH_4_–C_6_H_5_AsMo_6_**)

The polyanion **C_6_H_5_AsMo_6_** was synthesized by dissolving (NH_4_)_6_Mo_7_O_24_·4H_2_O (0.053 g, 0.04 mmol), N_2_H_4_·2HCl (0.011 g, 0.10 mmol), and phenylarsonic acid, C_6_H_5_AsO_3_H_2_ (0.01 g, 0.05 mmol) in 2 mL of 0.5 M sodium dimethylarsinate buffer (pH 7). The reaction mixture was stirred and heated at 80 °C for 1 h. Red block-shaped crystals formed within a day upon cooling to room temperature. Isolated yield: 0.025 g (32% based on Mo). Elemental analysis (%) calcd (found) for **NaNH_4_–C_6_H_5_AsMo_6_**: Na, 1.39 (1.15); Mo, 34.9 (34.4); As, 18.2 (18.5); C, 8.73 (9.04); H, 3.05 (3.21); N, 0.85 (1.07). FT-IR (2% KBr pellet, ν/cm^–1^): 3410 (s) [ν­(N–H), ν­(O–H)], 3014 (w), 2927 (w) [ν­(C–H)], 1623 (m) [δ­(O–H)], 1444 (w), 1401 (m) [δ­(C–H)], 1274 (w) [ν­(As–C)], 1097 (w), 969 (s), 939 (w), 912 (w) [ν­(Mo=O)], 826 (s) [ν­(As–O)], 746 (s), 692 (w), 655 (w), 612 (w), 524 (w), 493 (m), 458 (m) [δ­(Mo–O­(Mo))] (Figure S5e). ^1^H NMR (H_2_O/D_2_O, ppm): δ 1.6 (s, 9H, (C*H*_3_)_2_AsO_2_), 2.3 (s, 9H, (C*H*_3_)_2_AsO_2_), 7.07 (d, 2H, ^3^ *J*_HH_ = 8 Hz, *ortho*-C_6_ *H*_5_), 7.37 (t, 2H, ^3^J_HH_ = 8 Hz, *meta*-C_6_ *H*_5_), 7.46 (t, 1H, ^3^ *J*_HH_ = 8 Hz, *para*-C_6_ *H*_5_). ^13^C­{^1^H} NMR (H_2_O/D_2_O, ppm): δ 17.8 ((*C*H_3_)_2_AsO_2_), 19.5 ((*C*H_3_)_2_AsO_2_), 129.4 (*C*-3), 129.7 (*C*-2), 131.2 (*C*-4), 133.6 (*C*-1) (Figure S23).

High-quality single crystals of **Na–C_6_H_5_AsMo_6_** suitable for single-crystal XRD were obtained by heating Na_2_MoO_4_·2H_2_O (0.024 g, 0.10 mmol), N_2_H_4_·2HCl (0.011 g, 0.10 mmol), and phenylarsonic acid, C_6_H_5_AsO_3_H_2_ (0.01 g, 0.05 mmol), in 2 mL, 1 M sodium dimethylarsinate buffer (pH 7) at 80 °C for 1 h. The resulting product was characterized via IR spectroscopy (Figure S6).

#### Synthesis of NaNH_4_[4-FC_6_H_4_AsMo^V^_6_O_15_(OH)_3_{AsO_2_(CH_3_)_2_}_3_]·7H_2_O (**NaNH_4_–FC_6_H_4_AsMo_6_**)

The polyanion **4-FC_6_H_4_AsMo_6_** is synthesized in the same way as **C_6_H_5_AsMo_6_** by adding (4-fluorophenyl)­arsonic acid, 4-FC_6_H_4_AsO_3_H_2_ (0.011 g, 0.05 mmol), instead of C_6_H_5_AsO_3_H_2._ The reaction mixture (pH 6.7–6.8) was stirred and heated at 80 °C for 1 h. Dark-red, block-shaped crystals started appearing after 2 days as the solution cooled to room temperature. Isolated yield: 0.067 g (89% based on Mo). Elemental analysis (%) calcd (found) for **NaNH_4_–FC_6_H_4_AsMo_6_**: Na, 1.42 (1.54); Mo, 35.6 (36.1); As, 18.6 (19.1); F, 1.18 (1.02); C, 8.92 (8.37); H, 2.68 (2.77); N, 0.87 (1.03). FT-IR (2% KBr pellet, ν/cm^–1^): 3403 (s), 3214 (s) [ν­(N–H), ν­(O–H)], 3017 (w), 2920 (w) [ν­(C–H)], 1631 (m) [δ­(O–H)], 1591 (sh), 1496 (w) [ν­(C = C)], 1403 (m) [δ­(C–H)], 1273 (w) [ν­(As–C)], 1232 (w), 1164 (w), 1095 (w) [ν­(C–F)], 968 (s), 918 (w) [ν­(Mo=O)], 843 (s) [ν­(As–O)], 743 (m), 655 (w), 586 (w), 492 (m), 460 (m) [δ­(Mo–O­(Mo))] (Figure S7a). ^1^H NMR (H_2_O/D_2_O, ppm): δ 1.6 (s, 9H, (C*H*_3_)_2_AsO_2_), 2.3 (s, 9H, (C*H*_3_)_2_AsO_2_), 7.06 (m, 4H, C_6_ *H*_4_) (Figure S25). ^13^C­{^1^H} NMR (H_2_O/D_2_O, ppm): δ 17.9 ((*C*H_3_)_2_AsO_2_), 19.7 ((*C*H_3_)_2_AsO_2_), 116.9 (d, ^2^ *J*_CF_ = 22.14 Hz, *C*-3), 127.2 (s, *C*-1), 132.5 (d, ^3^ *J*_CF_ = 8.44 Hz, *C*-2), 165.7 (d, ^1^ *J*_CF_ = 261.35 Hz, *C*-4). ^19^F NMR (H_2_O/D_2_O, ppm): δ −104.5 (Figure S26).

#### Synthesis of NaNH_4_[4-F_3_CC_6_H_4_AsMo^V^_6_O_15_(OH)_3_{AsO_2_(CH_3_)_2_}_3_]·11H_2_O (**NaNH_4_–F_3_CC_6_H_4_AsMo_6_**)

The polyanion **4-F_3_CC_6_H_4_AsMo_6_** is prepared like **C_6_H_5_AsMo_6_** by replacing C_6_H_5_AsO_3_H_2_ with (4- trifluoromethylphenyl)­arsonic acid, 4-F_3_CC_6_H_4_AsO_3_H_2_ (0.013 g, 0.05 mmol). The reaction mixture (pH 6.7–6.8) was stirred and heated at 80 °C for 1 h. The total isolated yield of red, block-shaped crystals after 2 days is 0.07 g (86% based on Mo). Elemental analysis (%) calcd (found) for **NaNH_4_–F_3_CC_6_H_4_AsMo_6_**: Na, 1.32 (1.13); Mo, 33.1 (32.9); As, 17.2 (17.6); F, 3.28 (3.45); C, 8.99 (8.41); H, 2.96 (2.79); N, 0.81 (1.24). FT-IR (2% KBr pellet, ν/cm^–1^): 3418 (s), 3199 (s) [ν­(N–H), ν­(O–H)], 3017­(w), 2928 (w) [ν­(C–H)], 1636 (s) [δ­(O–H)], 1402 (s) [δ­(C–H)], 1326 (s), 1273 (w) [ν­(As–C)], 1173 (m), 1132 (m), 1060 (m) [ν­(C–F)], 1012 (sh), 969 (m), 924 (w) [ν­(Mo=O)], 844 (s) [ν­(As–O)], 744 (m), 655 (w), 590 (m), 494 (m), 465 (m) [δ­(Mo–O­(Mo))] (Figure S7b). ^1^H NMR (H_2_O/D_2_O, ppm): δ 1.6 (s, 9H, (C*H*_3_)_2_AsO_2_), 2.3 (s, 9H, (C*H*_3_)_2_AsO_2_), 7.18 (d, 2H, ^3^J_HH_ = 8 Hz, *ortho*-C_6_ *H*_4_), 7.67 (d, 2H, ^3^ *J*_HH_ = 8 Hz, *meta*-C_6_ *H*_4_) (Figure S27). ^13^C­{^1^H} NMR (H_2_O/D_2_O, ppm): δ 17.7 ((*C*H_3_)_2_AsO_2_), 19.5 ((*C*H_3_)_2_AsO_2_), 123.3 (q, ^1^ *J*_CF_ = 272.4 Hz, *C*F_3_), 126.2 (s, *C*-3), 130.3 (s, *C*-2), 134.3 (q, ^2^ *J*_CF_ = 32.2 Hz, *C*-4), 135.3 (s, *C*-1). ^19^F NMR (H_2_O/D_2_O, ppm): δ −63.1 (Figure S28).

#### Synthesis of NaNH_4_[4-F_3_COC_6_H_4_AsMo^V^_6_O_15_(OH)_3_{AsO_2_(CH_3_)_2_}_3_]·10H_2_O (**NaNH_4_–F_3_COC_6_H_4_AsMo_6_**)

The polyanion **4-F_3_COC_6_H_4_AsMo_6_** was prepared by following the same procedure as for **C_6_H_5_AsMo_6_**, by replacing C_6_H_5_AsO_3_H_2_ with (4-trifluoromethoxyphenyl)­arsonic acid, 4-F_3_COC_6_H_4_AsO_3_H_2_ (0.012 g, 0.05 mmol). The reaction mixture (pH 6.7–6.8) was stirred and heated at 80 °C for 1 h. After 2 days of cooling to room temperature, dark-red, rod-shaped crystals (Figure S4d) began to form. The total isolated yield is 0.06 g (74% based on Mo). Elemental analysis (%) calcd (found) for **NaNH_4_–F_3_COC_6_H_4_AsMo_6_**: Na, 1.32 (1.32); Mo, 33.2 (32.2); As, 17.3 (17.3); F, 3.28 (3.36); C, 9.0 (9.4); H, 2.8 (2.4); N, 0.81 (1.21). FT-IR (2% KBr pellet, ν/cm^–1^): 3403 (s), 3208 (s) [ν­(N–H), ν­(O–H)], 3023 (w), 2929 (w) [ν­(C–H)], 1630 (s) [δ­(O–H)], 1495 (m), 1404 (s) [δ­(C–H)], 1259 (s) [ν­(As–C)], 1217 (s), 1171 (s) [ν­(C–F)], 1097 (w), 969 (s) [ν­(Mo=O)], 829 (s) [ν­(As–O)], 745 (s), 657 (w), 612 (w), 494 (m), 459 (m) [δ­(Mo–O­(Mo))] (Figure S7e). ^1^H NMR (H_2_O/D_2_O, ppm): δ 1.6 (s, 9H, (C*H*_3_)_2_AsO_2_), 2.3 (s, 9H, (C*H*_3_)_2_AsO_2_), 7.07 (d, 2H, ^3^ *J*_HH_ = 8 Hz, *ortho*-C_6_ *H*_4_), 7.23 (d, 2H, ^3^ *J*_HH_ = 8 Hz, *meta*-C_6_ *H*_4_). ^19^F NMR (H_2_O/D_2_O, ppm): δ −57.7 (Figure S29). ^13^C­{^1^H} NMR (H_2_O/D_2_O, ppm): δ 17.6 ((*C*H_3_)_2_AsO_2_), 19.4 ((*C*H_3_)_2_AsO_2_), 118.6 (O*C*F_3_), 121.4 (s, *C*-3), 129.6 (s, *C*-4), 131.8 (s, *C*-2), 152.4 (s, *C*-1) (Figure S30).

#### Synthesis of Na_0.7_(NH_4_)_1.3_[4-Br­C_6_­H_4_­AsMo^V^_6_O_15_­(OH)_3_{AsO_2_­(CH_3_)_2_}_3_]·8H_2_O (**NaNH_4_–BrC_6_H_4_AsMo_6_**)

The polyanion **4-BrC_6_H_4_AsMo_6_** was prepared by following the same procedure as **C_6_H_5_AsMo_6_**, by replacing C_6_H_5_AsO_3_H_2_ with (4-bromophenyl)­arsonic acid, 4-BrC_6_H_4_AsO_3_H_2_ (0.014 g, 0.05 mmol). After stirring and heating the reaction mixture (pH 6.7–6.8) at 80 °C for 1 h, it was allowed to cool gradually to room temperature over 2 days, leading to the formation of dark-red, rod-shaped crystals. The total isolated yield is 0.069 g (87% based on Mo). Elemental analysis (%) calcd (found) for **NaNH_4_–BrC_6_H_4_AsMo_6_:** Na, 0.95 (1.25); Mo, 34.0 (34.6); As, 17.7 (18.6); Br, 4.72 (4.15); C, 8.52 (7.87); H, 2.75 (2.77); N, 1.08 (1.31). FT-IR (2% KBr pellet, ν/cm^–1^): 3419 (s), 3179 (s) [ν­(N–H), ν­(O–H)], 3008 (w), 2926 (w) [ν­(C–H)], 1632 (m) [δ­(O–H)], 1573 (w), 1474 (sh), 1402 (m) [δ­(C–H)], 1272 (w) [ν­(As–C)], 1183 (w), 1095 (w), 1063 (w) [ν­(C–Br)], 1009 (w), 969 (s), 921 (w) [ν­(Mo=O)], 834 (s) [ν­(As–O)], 744 (s), 655 (w), 587 (w), 497 (m), 468 (m) [δ­(Mo–O­(Mo))] (Figure S7c). ^1^H NMR (H_2_O/D_2_O, ppm): δ 1.6 (s, 9H, (C*H*_3_)_2_AsO_2_), 2.3 (s, 9H, (C*H*_3_)_2_AsO_2_), 6.9 (d, 2H, ^3^ *J*_HH_ = 9 Hz, *meta*-C_6_ *H*_4_), 7.5 (d, 2H, ^3^ *J*_HH_ = 9 Hz, *ortho*-C_6_ *H*_4_). ^13^C­{^1^H} NMR (H_2_O/D_2_O, ppm): δ 17.7 ((*C*H_3_)_2_AsO_2_), 19.4 ((*C*H_3_)_2_AsO_2_), 128.1 (*C*-4), 130.2 (*C*-1), 131.1 (*C*-3), 132.5 (*C*-2) (Figure S32).

High-quality crystals for single-crystal XRD were synthesized by heating Na_2_MoO_4_·2H_2_O (0.024 g, 0.10 mmol), N_2_H_4_·2HCl (0.011 g, 0.1 mmol), and (4-bromophenyl)­arsonic acid, 4-BrC_6_H_4_AsO_3_H_2_ (0.014 g, 0.05 mmol), in 2 mL of 1 M sodium dimethylarsinate buffer (pH 7) at 80 °C for 1 h. The identity of the crystals was verified by IR spectroscopy (Figure S8).

#### Synthesis of Na_1.3_(NH_4_)_0.7_­[4-N_3_­C_6_H_4_­AsMo^V^_6_O_15_­(OH)_3_­{AsO_2_­(CH_3_)_2_}_3_]·10H_2_O (**NaNH_4_–N_3_C_6_H_4_AsMo_6_**)

The polyanion **4-N_3_C_6_H_4_AsMo_6_** was prepared by following the same procedure as **C_6_H_5_AsMo_6_**, by replacing C_6_H_5_AsO_3_H_2_ with (4-azidophenyl)­arsonic acid, 4-N_3_C_6_H_4_AsO_3_H_2_ (0.012 g, 0.05 mmol). Following 1 h of stirring and heating at 80 °C, the reaction mixture (pH 6.7–6.8) was left to cool at room temperature for 2 days, resulting in the formation of dark-red, rod-shaped crystals. Isolated yield: 0.072 g (91% based on Mo). Elemental analysis (%): Calcd (found) for **NaNH_4_–N_3_C_6_H_4_AsMo_6_**: Na, 1.76 (1.94); Mo, 34.0 (34.2); As, 17.7 (18.5); C, 8.5 (7.9); H, 2.8 (2.8); N, 3.06 (2.76). FT-IR (2% KBr pellet, ν/cm^–1^): 3403 (s), 3185 (s) [ν­(N–H), ν­(O–H)], 3014 (w), 2926 (w) [ν­(C–H)], 2129 (s) [ν­(N_3_)], 1634 (s) [δ­(O–H)], 1589 (sh), 1492 (sh), 1403 (s) [δ­(C–H)], 1276 (s) [ν­(As–C)], 1188 (w), 1133 (w), 1096 (w), 968 (m), 922 (w) [ν­(Mo=O)], 843 (s) [ν­(As–O)], 742 (m), 655 (w), 586 (m), 491 (m), 465 (m) [δ­(Mo–O­(Mo))] (Figure S7d). ^1^H NMR (H_2_O/D_2_O, ppm): δ 1.6 (s, 9H, (C*H*_3_)_2_AsO_2_), 2.3 (s, 9H, (C*H*_3_)_2_AsO_2_), 6.58 (d, 2H, ^3^ *J*_HH_ = 9 Hz, *ortho*-C_6_ *H*_4_), 6.72 (d, 2H, ^3^ *J*_HH_ = 9 Hz, *meta*-C_6_ *H*_4_) (Figure S34).

#### Synthesis of NaNH_4_­[3,5-(HOOC)_2_C_6_­H_3_AsMo^V^_6_O_15_­(OH)_3_{AsO_2_­(CH_3_)_2_}_3_]·14H_2_O (**NaNH_4_–H_2_O_4_C_2_C_6_H_3_AsMo_6_**)

The polyanion **H_2_O_4_C_2_C_6_H_3_AsMo_6_** was synthesized using the same procedure for **C_6_H_5_AsMo_6_**, substituting C_6_H_5_AsO_3_H_2_ with 3,5-bis­(carboxy)­phenylarsonic acid, 3,5-(HOOC)_2_C_6_H_3_AsO_3_H_2_ (0.015 g, 0.05 mmol). After stirring and heating at 80 °C for 1 h, the reaction mixture (pH 6.7–6.8) was cooled, centrifuged at 4000 rpm for 15 min, and filtered. The resulting red filtrate was left undisturbed in an open vial for slow crystallization. After 7 days, reddish block-shaped crystals were obtained. Isolated yield: 0.028 g (33% based on Mo). Elemental analysis (%): calcd (found) for **NaNH_4_–H_2_O_4_C_2_C_6_H_3_AsMo_6_**: Na, 1.27 (1.52); Mo, 31.8 (30.6); As, 16.6 (16.7); C, 9.29 (9.32); H, 3.23 (3.07); N, 0.77 (1.45). FT-IR (2% KBr pellet, ν/cm^–1^): 3445 (s), 3229 (s) [ν­(N–H), ν­(O–H)], 3022 (w), 2927 (w) [ν­(C–H)], 1635 (m) [δ­(O–H)], 1560 (m) [υ­(C=O)], 1401 (m) [δ­(C–H)], 1280 (w) [ν­(As–C)], 1108 (w), 970 (s) [ν­(Mo=O)], 840 (s) [ν­(As–O)], 746 (m), 699 (w), 656 (w), 488 (m), 460 (m) [δ­(Mo–O­(Mo))] (Figure S7f). ^1^H NMR (H_2_O/D_2_O, ppm): δ 1.5 (s, 9H, (C*H*_3_)_2_AsO_2_), 2.2 (s, 9H, (C*H*_3_)_2_AsO_2_), 7.6 (s, 2H, *ortho*-C_6_ *H*_3_), 8.3 (s, 1H, *para*-C_6_ *H*_3_). ^13^C­{^1^H} NMR (H_2_O/D_2_O, ppm): δ 17.8 ((*C*H_3_)_2_AsO_2_), 19.4 ((*C*H_3_)_2_AsO_2_), 131.8 (*C*-1), 133.1 (*C*-3), 134.0 (*C*-2), 135.6 (*C*-4), 171.0 (*C*OOH) (Figure 3).

### X-ray Crystallography

Single-crystal XRD data for **NaNH_4_–As^V^Mo_6_** were collected at 100 K using a Bruker D8 SMART APEX II CCD diffractometer with kappa geometry and a graphite monochromator (λ_Mo Kα_ = 0.71073 Å), with the APEX III software package. Cell refinement and data reduction were performed using SAINT, while multiscan absorption corrections were applied via SADABS.^26^ For **NaNH_4_–As^III^Mo_6_**, **NaNH_4_–CH_3_AsMo_6_**, **NaNH_4_–C_2_H_5_AsMo_6_**, **NaNH_4_–C_6_H_5_AsMo_6_**, **NaNH_4_–FC_6_H_4_AsMo_6,_** **NaNH_4_–F_3_CC_6_H_4_AsMo_6_**, **NaNH_4_–F_3_­COC_6_­H_4_As­Mo_6_**, **NaNH_4_–Br­C_6_H_4_As­Mo_6_**, **NaNH_4_–N_3_­C_6_H_4_As­Mo_6_** and **NaNH_4_–H_2_­O_4_C_2_­C_6_H_3_AsMo_6_**, indexing and data collection were carried out on a Rigaku XtaLAB Synergy Dualflex HyPix single-crystal diffractometer with kappa geometry and a graphite monochromator with Mo Kα/Cu Kα radiation (λ = 0.71073/1.54184 Å) (Table S1). All measurements were conducted with crystals mounted on Hampton cryoloops with paratone-N oil at 100 K. Corrections for absorption effects were applied with CrysAlisPro 1.171.43.97a. The structures were solved by direct methods using SHELXT, and full-matrix least-squares structure refinements were performed with SHELXL-2018/3 implemented in Olex2 1.5-ac5–024.^27^ All nonhydrogen atoms were refined anisotropically. In several crystal structures, the voids contain disordered solvents. The Olex2 solvent mask routine (similar to PLATON/SQUEEZE) was used to mask out the disordered electron density. Crystallographic data for the polyanions are summarized in Table S1. The CIF files are provided free of charge by The Cambridge Crystallographic Data Centre (CCDC 2464737–2464747).

### Bond Valence Sum Calculations

The bond valence sum (BVS) calculations for the molybdenum and oxygen atoms (Tables S2–S12) were conducted using a program developed by Chris Hormillosa and Sean Healy and distributed by I. D. Brown.^28^

### Antibacterial Activity: Determination of Minimal Inhibitory Concentrations (MICs) for Bacterial Cells

The MIC experiments were built upon our prior research, the procedures following our previous work and Elshamy et al.^29^ Precultures were established by inoculating single bacterial colonies into 5 mL of the appropriate liquid medium. After 24 h, the optical density (OD) at 600 nm was assessed. The resulting cell suspension was adjusted to a concentration of 2 × 10^6^ bacterial cells per mL. To determine the MICs, 96-well plates were set up with a dilution series of **NaNH_4_–As^V^Mo_6_**, **NaNH_4_–CH_3_­As­Mo_6_**, **Na­NH_4_–C_6_H_5_­AsMo_6_**, **Na­NH_4_–FC_6_­H_4_As­Mo_6_**, **Na­NH_4_–F_3_­CC_6_H_4_As­Mo_6_**, **Na­NH_4_–F_3_­COC_6_­H_4_As­Mo_6_**, **Na­NH_4_–N_3_­C_6_H_4_­As­Mo_6_**, **Na­NH_4_–Br­C_6_H_4_­AsMo_6_**, and **Na­NH_4_–H_2_­O_4_C_2_C_6_H_3_­AsMo_6_** beginning at 500 μg/mL. Stock solutions were prepared using sterile deionized water. The 96-well plates were incubated overnight at the optimal growth temperature for each organism tested. Outcomes were visually assessed, with MICs established based on the presence or absence of growth. For better visualization, resazurin was used as a fluorometric indicator to aid in determining the MICs. All MIC experiments were performed in triplicate, with three independent replicates. Agar diffusion and MIC are simple and widely known methods of determining the zone of inhibition and the lowest concentration of the samples against a defined organism, respectively.^30^

## Results and Discussion

### Synthesis and Structure

A one-pot aqueous reaction of ammonium heptamolybdate, hydrazine hydrochloride, organoarsonate heterogroup, and sodium cacodylate buffer in a 1:2:1:20 molar ratio yielded a series of dimethylarsinate-functionalized arsenomolybdates: [CH_3_As­Mo^V^_6_­O_15_­(OH)_3_­{AsO_2_­(CH_3_)_2_}_3_]^2–^ (**CH_3_As­Mo_6_**), [C_6_H_5_­AsMo^V^_6_­O_15_(OH)_3_­{AsO_2_­(CH_3_)_2_}_3_]^2–^ (**C_6_­H_5_AsMo_6_**), [FC_6_H_4_­AsMo^V^_6_­O_15_(OH)_3_­{AsO_2_­(CH_3_)_2_}_3_]^2–^ (**4-FC_6_­H_4_AsMo_6_**), [F_3_­CC_6_H_4_­AsMo^V^_6_­O_15_(OH)_3_­{AsO_2_­(CH_3_)_2_}_3_]^2–^ (**4-F_3_­CC_6_H_4_As­Mo_6_**), [F_3_­COC_6_­H_4_As­Mo^V^_6_O_15_­(OH)_3_­{AsO_2_(CH_3_)_2_}_3_]^2–^ (**4-F_3_­COC_6_H_4_­As­Mo_6_**), [Br­C_6_H_4_­As­Mo^V^_6_­O_15_(OH)_3_­{AsO_2_­(CH_3_)_2_}_3_]^2–^ (**4-Br­C_6_H_4_­As­Mo_6_**), [N_3_­C_6_H_4_­AsMo^V^_6_­O_15_­(OH)_3_­{AsO_2_­(CH_3_)_2_}_3_]^2–^ (**4-N_3_­C_6_H_4_As­Mo_6_**), and [3,5-(HOOC)_2_­C_6_H_3_­As­Mo^V^_6_O_15_­(OH)_3_{AsO_2_­(CH_3_)_2_}_3_]^2–^ (**H_2_O_4_­C_2_­C_6_H_3_­AsMo_6_**), all isolated as hydrated mixed ammonium-sodium salts. These compounds were successfully synthesized within a pH range of 6.4–6.8 at room temperature, 50, or 80 °C. In each case, the formation of a dark-red solution indicated the reduction of Mo^VI^ to Mo^V^, which was confirmed by bond valence sum (BVS) calculations (Tables S4 and S6–S12). The highest isolated yields were achieved at 80 °C, with crystallization occurring within 1 week. Substituting ammonium heptamolybdate ((NH_4_)_6_Mo_7_O_24_·4H_2_O) with sodium molybdate (Na_2_MoO_4_·2H_2_O) under identical conditions resulted in high-quality crystals, albeit at significantly lower yields. For the polyanion **H_2_O_4_C_2_C_6_H_3_AsMo_6_**, BVS calculations indicated that the carboxylate moieties are monoprotonated (Table S12). Attempts to prepare the structural analogue using 4-HOOCC_6_H_4_AsO_3_H_2_ as the precursor ligand were unsuccessful. Despite numerous reactions and pH adjustments, the reported [Mo^VI^_18_Mo^V^_12_O_84_{AsO_2_(CH_3_)_2_}_18_]^18–^ {**Mo_30_**} was formed instead.^31^

Synthesis of the three isostructural polyanions [HO­As­Mo^V^_6_­O_15_­(OH)_3_­{As­O_2_­(CH_3_)_2_}_3_]^2–^ (**As^V^­Mo_6_**), [As^III^­Mo^V^_6_­O_15_(OH)_3_­{AsO_2_­(CH_3_)_2_}_3_]^3–^ (**As^III^Mo_6_**), and [C_2_H_5_­AsMo^V^_6_­O_15_(OH)_3_­{As­O_2_­(CH_3_)_2_}_3_]^2–^ (**C_2_­H_5_­As­Mo_6_**) required precise control over the molybdate precursor, reactant ratios, temperature, and pH. The **As^V^Mo_6_** polyanion was obtained under specific conditions, requiring a 1:2:1 molar ratio of ammonium heptamolybdate, hydrazine hydrochloride, and arsenate heterogroup in 10 mL of 0.5 M sodium cacodylate buffer at pH 6.6–6.8 and a reaction temperature of 80 °C. Attempts to use sodium molybdate or reduce the reaction temperature were unsuccessful. Structural analysis of **As^V^Mo_6_** indicated the monoprotonation of terminal oxygen (O9) in the As–O bond (1.689 Å), with a corresponding BVS value (*v*_*ij*_ = 1.24) (Table S2).

Conversely, **As^III^Mo_6_** and **C_2_H_5_AsMo_6_** were only obtained using sodium molybdate as the molybdenum source. The optimal molar ratio of sodium molybdate to As_2_O_3_ or C_2_H_5_AsO_3_H_2_ was 2:1, with reactions carried out at 80 °C within a pH range of 6.8–6.9. For these three species, prolonged heating did not improve yield, and crystallization occurred more slowly than the other members of the series.

Single-crystal XRD analysis confirmed that the molecular structures of these polyanions, [RAs­Mo^V^_6_­O_15_­(OH)_3_­{As­O_2_­(CH_3_)_2_}_3_]^2–^ (R = HO, CH_3_, C_2_H_5_, C_6_H_5_, 4-FC_6_H_4_, 4-F_3_­CC_6_­H_4_, 4-F_3_­COC_6_­H_4,_ 4-Br­C_6_H_4_, 4-N_3_­C_6_H_4_, and 3,5-(HOOC)_2_­C_6_H_3_) (Figure 1a) and [As^III^­Mo^V^_6_­O_15_­(OH)_3_­{As­O_2_­(CH_3_)_2_}_3_]^3–^ (**As^III^Mo_6_**) (Figure 1b) closely resemble the previously reported [RPMo^V^_6_O_15_(OH)_3_{AsO_2_(CH_3_)_2_}_3_]^2–^ (R = H, HO, CH_3_, HO_2_CCH_2_, HO_2_CC_2_H_4_, C_6_H_5_, 4-FC_6_H_4_, and 4-F_3_COC_6_H_4_)^4^ and exhibit a distinct flowerpot-like architecture. The core structure consists of a cyclic hexanuclear molybdenum­(V)-oxo ring, formed by three edge-sharing {Mo^V^_2_O_10_} fragments interconnected via hydroxo bridges (verified through BVS calculations, see Tables S2–S12). These units are capped by three dimethylarsinate ((CH_3_)_2_AsO_2_), also known as cacodylate ligands, which act as strongly coordinating bidentate ligands. This arrangement features alternating short Mo–Mo bonding interactions (∼2.59 Å) and longer Mo···Mo nonbonding contacts (∼3.58 Å). The reduced Mo^V^_2_ pairs adopt a highly distorted octahedral coordination geometry, with each molybdenum center coordinated by two μ_2_-oxo groups, Mo–O­(Mo) bonds: 1.939–1.949 Å, one μ_2_-hydroxo group, Mo–O­(Mo) bond: 2.115–2.124 Å, one terminal oxo group, Mo=O_term_ bond: 1.682–1.696 Å, one oxo-donor from the terminal dimethylarsinate, Mo–O­(As) bond: 2.079–2.107 Å, and an oxo-donor from the central heterogroup, Mo–O­(As) bond: 2.212–2.304 Å.

**Figure 1:**
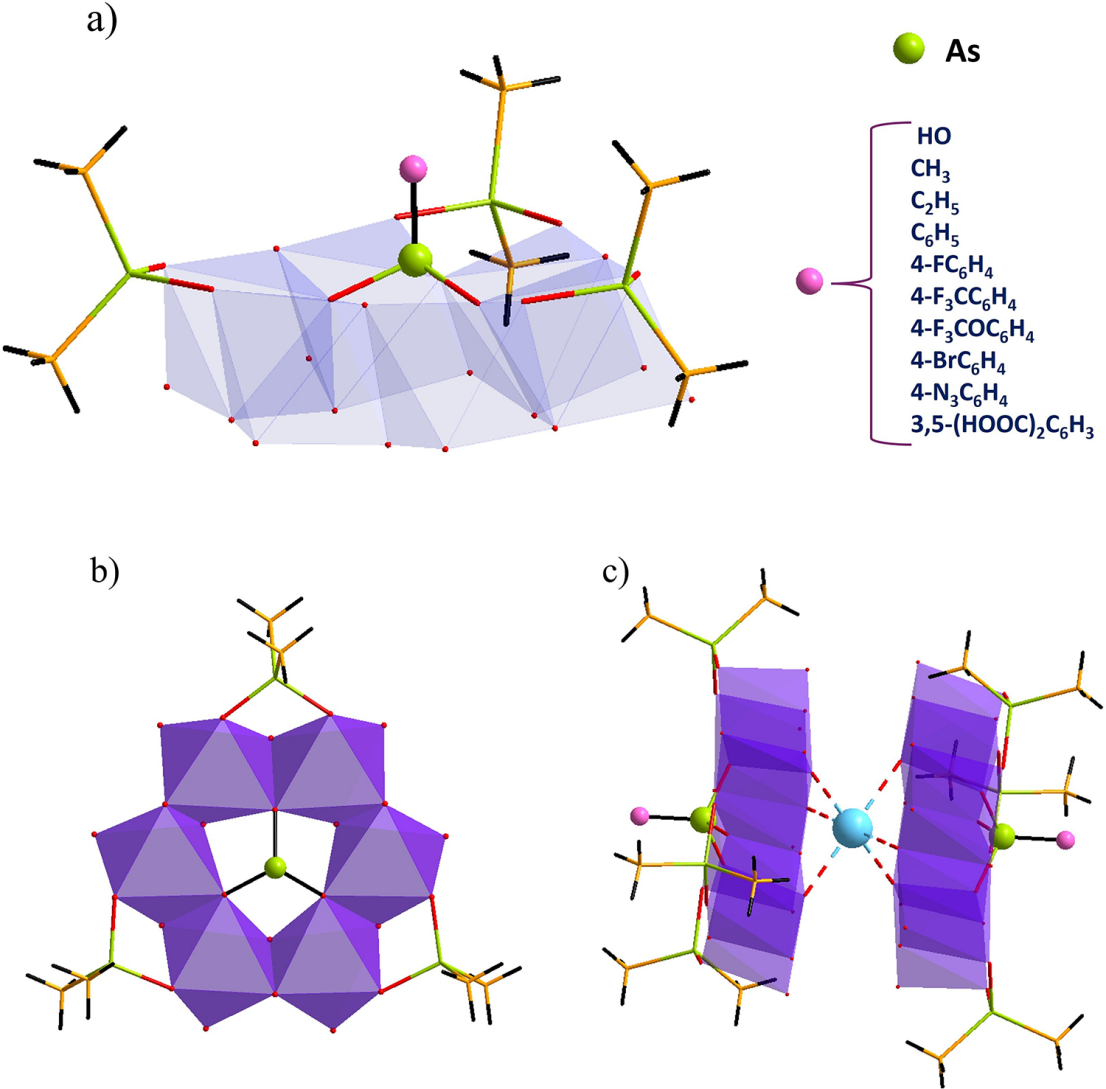
(a) Combined polyhedral/ball-and-stick representation of the polyanion family [RAsMo^V^_6_O_15_(OH)_3_{AsO_2_(CH_3_)_2_}_3_]^2–^ (R = HO, CH_3_, C_2_H_5_, C_6_H_5_, 4-FC_6_H_4_, 4-F_3_CC_6_H_4_, 4-F_3_COC_6_H_4_, 4-BrC_6_H_4_, 4-N_3_C_6_H_4_, and 3,5-(HOOC)_2_C_6_H_3_), and (b) the As^III^-centered derivative [As^III^Mo^V^_6_O_15_(OH)_3_{AsO_2_(CH_3_)_2_}_3_]^3–^, and (c) sodium ion-mediated dimer (formed by all polyanions in the solid state). Color code: MoO_6_, violet octahedra; As, green; C, yellow; O, red; H, black; Na, light blue.

The central RAsO_3_ heterogroups, adopting slightly distorted tetrahedral coordination, are positioned above the plane of the six molybdenum atoms and coordinate via three μ_3_-oxo bridges. Notably, the As^III^ center in **As^III^Mo_6_**, which adopts pyramidal geometry due to its lone pair, exhibits stronger Mo–O­(As) bonding (2.212 Å) compared to the As^V^ center in **As^V^Mo_6_** (2.289 Å). Additionally, all three peripheral dimethylarsinate ligands feature tetrahedrally coordinated As centers, with average As–O­(Mo) bond lengths ranging from 1.67 to 1.80 Å. The average bond angles around As closely resemble those in uncoordinated dimethylarsinic acid (109.4°), and the As–C distances (1.90–1.94 Å) fall within the expected range, consistent with literature values.^11b^ Both the peripheral ligands and the central heterogroups are grafted onto the same side of the polyanion.

Introducing a certain concentration of Na^+^ cations plays a crucial role in the polyanion formation, as evidenced by the solid-state structure (Figure 1c). A hexa-coordinated Na^+^ ion interacts with oxo bridges from the Mo^V^_2_ pairs (Na···O = 2.27 Å), on the opposite side of the coordinated dimethylarsinate ligands, thus facilitating Na^+^-mediated dimerization of the polyanions.

The precise formula units of the polyanions were determined through elemental analysis, thermogravimetric analysis (TGA) of the bulk material, bond valence sum (BVS) analysis, and single-crystal XRD analysis. The thermograms (Figures S9–S19) indicated two primary stages of weight loss. The first stage, between 25 and 130 °C, corresponds to dehydration, during which interstitial water molecules were released from the structure. The number of crystal water molecules identified by TGA and elemental analysis varied due to differences in drying times and the aging of the samples at room temperature prior to measurements. The consecutive weight loss steps between 160–480 °C were attributed to the elimination of ammonia (NH_3_) molecules, organic dimethylarsinate ligands, and central organic moieties, leading to complete structural decomposition.

### Infrared (IR) Spectroscopy

Fourier transform infrared (FT-IR) spectra (Figures S5–S8) display bands corresponding to the asymmetric stretching (υ_as_[O–H]) and bending (δ­[O–H]) vibrations of interstitial water molecules in the regions of 3400–3000 and 1640–1620 cm^–1^. Furthermore, broad peaks in the 3600–3400 cm^–1^ range indicate the stretching vibrations of the NH_4_^+^ group. The presence of NH_4_^+^ as counter cations in the clusters is confirmed by elemental analysis. Two weak peaks in the 3030–2800 cm^–1^ range, along with medium-intensity bands near 1404 cm^–1^, are attributed to asymmetric stretching (υ_as_[C–H]) and bending (δ­[C–H]) vibrations of the methyl groups in the dimethylarsinate ligands.^4^ The characteristic bands observed between 980–850 and 600–400 cm^–1^ are assigned to the asymmetric stretching (υ_as_[Mo=O]) and (υ_as_[Mo–O­(Mo)]) vibrations. Strong bands at 850–820 cm^–1^ and medium-intensity peaks at 1280–1250 cm^–1^ are associated with asymmetric stretching (υ_as_[As–O]) and (υ_as_[As–C]) vibrations. The strong band at 2120 cm^–1^, attributed to the asymmetric stretching of the azido group, and weak bands for asymmetric C–F stretching between 1200 and 1000 cm^–1^ (Figure S7) confirm the incorporation of phenylarsonate derivatives. The C–Br stretching occurs between 1093 and 1063 cm^–1^ (Figure S7).

### Nuclear Magnetic Resonance (NMR) Studies

The solution behavior of the arsenic-containing polyanions [As^III^Mo^V^_6_O_15_(OH)_3_{AsO_2_(CH_3_)_2_}_3_]^3–^ and [RAsMo^V^_6_O_15_(OH)_3_{AsO_2_(CH_3_)_2_}_3_]^2–^ (R = HO, CH_3_, C_2_H_5_, C_6_H_5_, 4-FC_6_H_4_, 4-F_3_COC_6_H_4_, 4-F_3_CC_6_H_4_, 4-BrC_6_H_4_, 4-N_3_C_6_H_4_, and 3,5-(HOOC)_2_C_6_H_3_) was examined using multinuclear (^1^H, ^19^F, and ^13^C­{^1^H}) NMR spectroscopy following the redissolution of the solid salts in a H_2_O/D_2_O mixture. These polyanions demonstrated greater solubility in water than their phosphorus-containing counterparts.^4^ Most polyanions dissolved readily at room temperature, except for **NaNH_4_–CH_3_AsMo_6_** and **NaNH_4_–C_2_H_5_AsMo_6_**, which required heating to 40 °C.

The reference compounds, including dimethylarsinic acid, (CH_3_)_2_AsO_2_H (cacodylic acid, **H-Cac**, pH 4.10), and sodium dimethylarsinate, (CH_3_)_2_AsO_2_Na (sodium cacodylate, **Na-Cac**, pH 6.0), displayed distinct NMR signals at δ = 1.8 and 1.6 ppm (^1^H) and δ = 17.0 and 16.7 ppm (^13^C­{^1^H}) in H_2_O/D_2_O, corresponding to the two equivalent methyl groups attached to the arsenic center. For the bioactive polyanions **NaNH_4_–C_6_H_5_AsMo_6_**, **NaNH_4_–F_3_COC_6_H_4_AsMo_6_**, and **NaNH_4_–BrC_6_H_4_AsMo_6_**, the solutions were carefully adjusted to pH 7 using a 1 M NaOH solution prior to the measurements.

The ^1^H NMR spectra of polyanions **As^III^Mo_6_** and **As^V^Mo_6_** exhibited distinct singlets at δ = 2.0 and 1.6 ppm (intensity ratio 1:1) for the former and δ = 2.2 and 1.9 ppm (intensity ratio 1:1) for the latter (Figure 2a). The ^13^C­{^1^H} NMR spectra showed sharp resonances at δ = 19.4 and 17.9 ppm for **As^III^Mo_6_** and closely spaced peaks at δ = 18.9 and 18.7 ppm for **As^V^Mo_6_** (Figure 2b), corresponding to the nonequivalent methyl groups of the coordinated dimethylarsinate moiety, consistent with the solid-state structure. Although all cacodylates in the polyanions are structurally equivalent, their two methyl groups are not. A careful examination of the polyanion structure reveals that one methyl group points toward the heterogroup at the center, while the other extends outward, resulting in structural and thus magnetic inequivalence. The minor peaks at 1.62 ppm (^1^H) and 17.0 ppm (^13^C­{^1^H}) in the **As^III^Mo_6_** spectra were attributed to decomposed free cacodylate.

**Figure 2:**
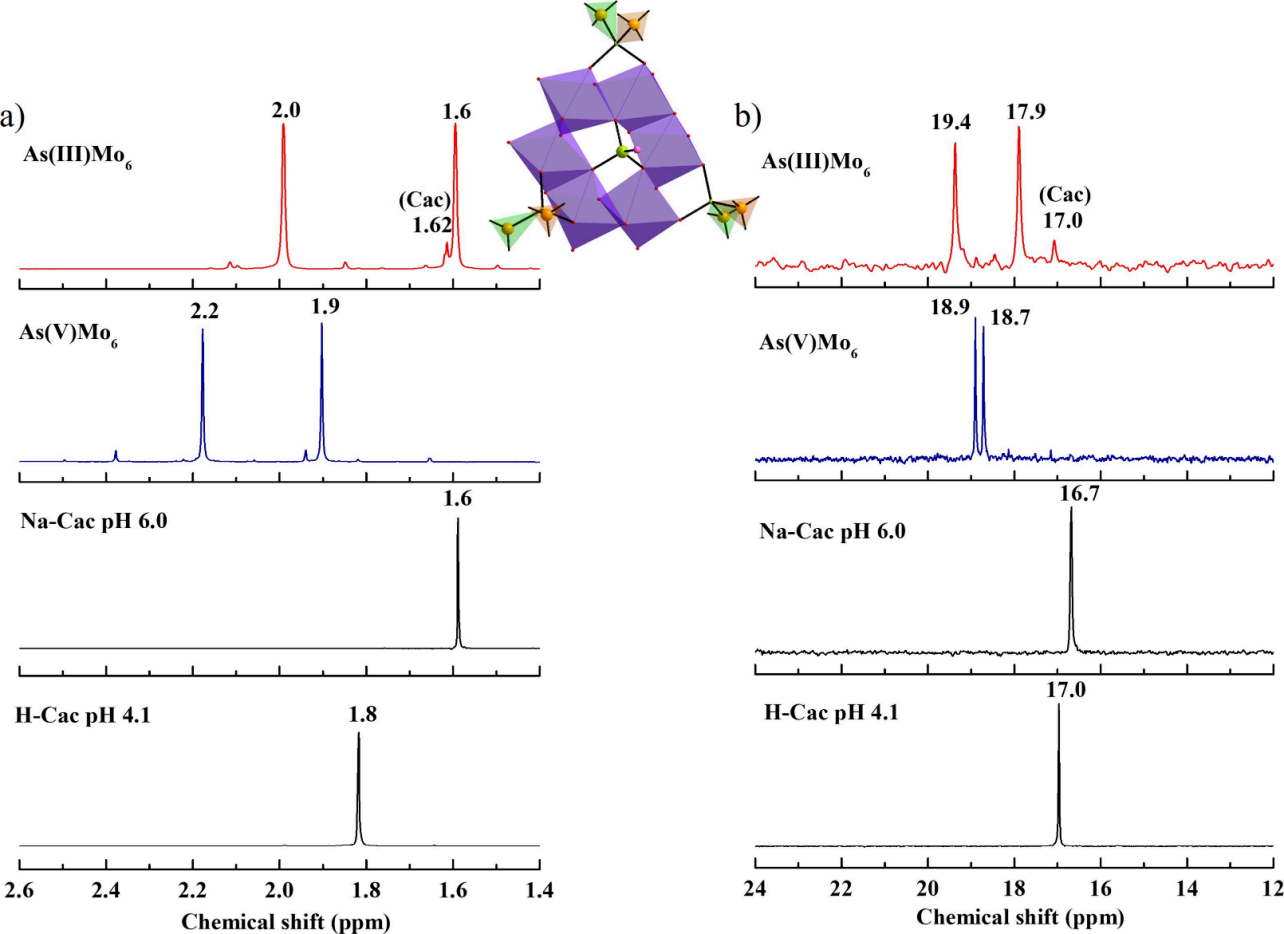
Multinuclear NMR study: (a) ^1^H and (b) ^13^C­{^1^H} NMR spectra of **NaNH_4_-As^III^Mo_6_** (red) and **NaNH_4_-As^V^Mo_6_** (blue), dissolved in H_2_O/D_2_O at room temperature. The spectra of the reference compounds, i.e., cacodylic acid (**H-Cac**, pH 4.10) and sodium cacodylate (**Na-Cac**, pH 6.0) buffer, are shown in black.

For the methylarsonate analogue **CH_3_AsMo_6_**, the peaks at δ = 2.4 and 2.0 ppm (^1^H) (Figure S20a) and δ = 19.7 and 18.0 ppm (^13^C­{^1^H}) (Figure S20b) correspond to the nonequivalent methyl groups of the coordinated cacodylate ligands, as expected. The signals at δ = 0.96 ppm (^1^H) and δ = 18.9 ppm (^13^C­{^1^H}) are attributed to the central methyl arsonate ligand. Additionally, minor decomposition of the bound cacodylate moieties is evident from peaks at δ = 1.7 ppm (^1^H) and δ = 17.1 ppm (^13^C­{^1^H}). The solution stability of **As^V^Mo_6_** and **CH_3_AsMo_6_** was monitored via ^13^C­{^1^H} NMR over 1 week. No spectral changes were observed for **As^V^Mo_6_**, indicating complete stability, whereas **CH_3_AsMo_6_** exhibited minor cacodylate decomposition over time (Figure S21). Overall, these findings confirm that the cacodylate groups remain strongly bound to the polyanion, consistent with a previous report.^4^

For the ethylarsonate analogue **C_2_H_5_AsMo_6_**, the ^1^H NMR spectrum displayed singlets at δ = 2.3 and 1.9 ppm (methyl groups of cacodylates), a quartet at δ = 1.2 ppm (−CH_2_), and a triplet at δ = 0.6 ppm (−CH_3_, ^3^ *J* = 8 Hz) (Figure S22). Due to the compound’s low solubility, even after heating to 40 °C, obtaining a reliable ^13^C­{^1^H} NMR spectrum was challenging.

The ^1^H NMR spectrum of **C_6_H_5_AsMo_6_** exhibited resonances at δ = 2.3 and 1.6 ppm (methyl groups of the coordinated cacodylate ligands). In the aromatic region, a doublet at δ = 7.07 ppm (^3^J = 8 Hz) and triplets at δ = 7.37 ppm (^3^ *J* = 8 Hz) and 7.46 ppm (^3^ *J* = 8 Hz) were assigned to the *ortho*, *meta*, and *para* protons of the phenylarsonate framework, respectively (Figure S23a). The ^13^C­{^1^H} NMR spectrum showed corresponding signals at δ = 19.5 and 17.8 ppm (methyl groups of cacodylates) along with peaks at δ = 129.40 (*meta*), 129.72 (*ortho*), 131.18 (*para*), and 133.64 ppm (*ipso*) (Figure S23b). Additionally, minor peaks at δ = 1.8 ppm (^1^H) and 17.0 ppm (^13^C­{^1^H}) were attributed to slight cacodylate decomposition. A one-week analysis of the ^1^H and ^13^C­{^1^H} NMR spectra confirmed the polyanion’s stability, apart from minor decomposition of the cacodylate ligands (Figure S24).

In the ^19^F NMR spectra of the fluorinated polyanions **4-FC_6_H_4_AsMo_6_**, **4-F_3_CC_6_H_4_AsMo_6_**, and **4-F_3_COC_6_H_4_AsMo_6_**, singlet resonances were observed at −104.5 ppm (Figure S26a), −63.1 ppm (Figure S28a), and −57.7 ppm (Figure S29a), respectively. In the ^1^H NMR spectrum of **4-FC_6_H_4_AsMo_6_**, unresolved multiplets were observed around 7.06 ppm. In contrast, the spectrum of **4-F_3_CC_6_H_4_AsMo_6_** showed well-resolved doublets corresponding to the *ortho* and *meta* aromatic protons at 7.18 and 7.67 ppm, respectively. The ^13^C­{^1^H} NMR spectra of **4-FC_6_H_4_AsMo_6_** and **4-F_3_CC_6_H_4_AsMo_6_** displayed signals at δ = 19.7 and 17.9 ppm (Figure S26b) and δ = 19.5 and 17.7 ppm (Figure S28b), respectively, assigned to the methyl carbons of the coordinated cacodylate moieties. For **4-FC_6_H_4_AsMo_6_**, carbon–fluorine coupling resulted in characteristic doublets at δ = 165.7 ppm (*para*, ^1^ *J*_C–F_ = 261.35 Hz), 132.5 ppm (*ortho*, ^3^ *J*_C–F_ = 8.44 Hz), and 116.9 ppm (*meta*, ^2^ *J*_C–F_ = 22.14 Hz), while the *ipso* carbon appeared as a singlet at δ = 127.2 ppm. In the trifluoromethyl analogue **4-F_3_CC_6_H_4_AsMo_6_**, quartets at δ = 123.3 ppm (−CF_3_, ^1^ *J*_C–F_ = 272.4 Hz) and 134.3 ppm (*para*, ^2^ *J*_C–F_ = 32.2 Hz) were observed due to carbon–fluorine coupling from the trifluoromethyl group. The peaks at δ = 126.2, 130.3, and 135.3 ppm were assigned to the *meta*, *ortho*, and *ipso* carbons, respectively. In both cases, the minor peak at δ = 17.0 ppm was attributed to free cacodylate, and in **4-F_3_CC_6_H_4_AsMo_6_**, an additional unassigned signal was detected at δ = 20.0 ppm.

The ^1^H NMR spectrum of the polyanion **4-F_3_COC_6_H_4_AsMo_6_** displayed characteristic resonances at δ = 1.6 and 2.3 ppm (methyl groups of cacodylates) along with peaks in the aromatic region, doublets at δ = 7.07 ppm (^3^ *J* = 8 Hz) and 7.23 ppm (^3^ *J* = 8 Hz) corresponding to the *ortho* and *meta* protons of the phenyl ring, respectively (Figure S29b). The ^13^C­{^1^H} NMR spectrum provided peaks at δ = 152.4 ppm (*ipso*), 131.8 ppm (*ortho*), 129.6 ppm (*para*), 121.4 ppm, (*meta*) and 118.6 ppm (−OCF_3_) in the aromatic region. Long-term monitoring of the ^1^H and ^19^F NMR spectra over 1 week confirmed the polyanion’s stability, aside from minor cacodylate decomposition (Figure S31).

For the polyanion **4-BrC_6_H_4_AsMo_6_**, the ^1^H NMR spectrum gave doublets at δ = 6.9 ppm (*meta*, ^3^ *J* = 9 Hz) and 7.5 ppm (*ortho*, ^3^ *J* = 9 Hz) in the aromatic region (Figure S32a). The ^13^C­{^1^H} NMR spectrum revealed resonances at δ = 128.1 ppm (*para*), 130.2 ppm (*ipso*), 131.1 ppm (*meta*), and 132.5 ppm (*ortho*), corresponding to the aromatic carbons (Figure S32b). Distinct peaks at δ = 2.3 and 1.6 ppm (^1^H) and δ = 19.4 and 17.7 ppm (^13^C­{^1^H}) were assigned to the inequivalent methyl groups of the coordinated cacodylate moieties. Additionally, peaks at δ = 1.7 ppm (^1^H) and δ = 16.9 ppm (^13^C­{^1^H}) were attributed to free cacodylate species. Time-dependent ^1^H NMR spectra suggested decomposition of the cacodylate moiety without significant structural changes (Figure S33).

The polyanion **4-N_3_C_6_H_4_AsMo_6_** exhibited the expected ^1^H NMR signals for the cacodylate methyl protons at δ = 2.3 and 1.6 ppm. Aromatic phenyl protons appeared as doublets at δ = 6.58 ppm (*ortho*, ^3^ *J* = 9 Hz) and 6.72 ppm (*meta*, ^3^ *J* = 9 Hz) (Figure S34). However, additional unassigned peaks at δ = 6.97, 7.59, and 7.64 ppm persisted in time-dependent studies, suggesting partial decomposition of the centrally bound ligand and instability of the polyanion upon dissolution.

For the polyanion **H_2_O_4_C_2_C_6_H_3_AsMo_6_**, ^1^H NMR showed peaks at δ = 7.6 and 8.3 ppm, corresponding to the *ortho* and *para* aromatic protons, respectively, and signals at δ = 1.5 and 2.2 ppm for the methyl protons of the coordinated cacodylate. In the ^13^C­{^1^H} NMR spectrum, signals at δ = 171.0, 135.6, 133.1, 134.0, and 131.8 ppm were assigned to the carboxylate group and the *para*, *meta*, *ortho*, and *ipso* carbons of the phenyl ring, respectively. Peaks at 19.4 and 17.8 ppm correspond to the bound cacodylates (Figure 3). As in previous cases, signals at δ = 1.8 ppm (^1^H) and 16.9 ppm (^13^C­{^1^H}) were attributed to free cacodylate species.

**Figure 3:**
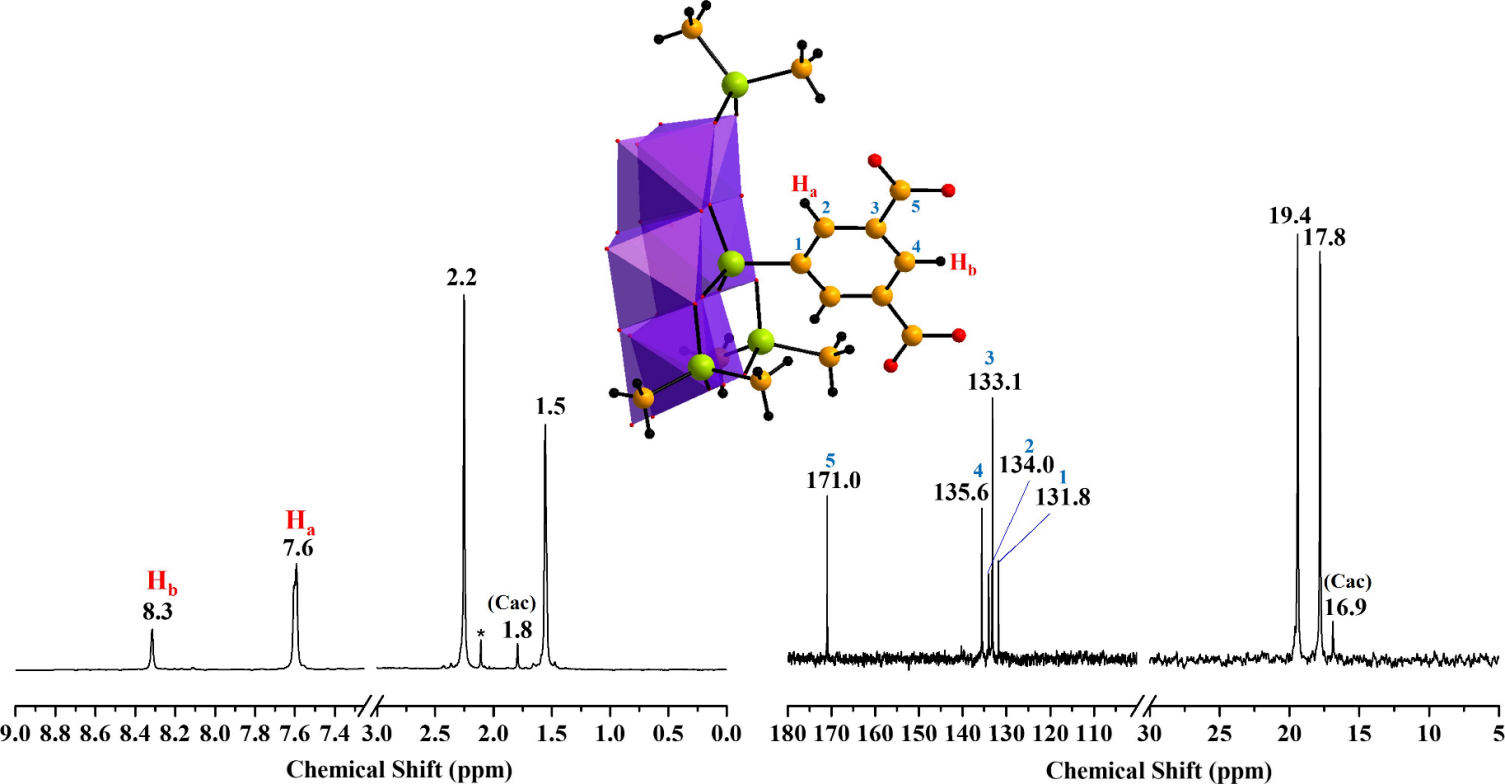
^1^H (left) and ^13^C­{^1^H} (right) NMR spectra of **NaNH_4_–H_2_O_4_C_2_C_6_H_3_AsMo_6_** dissolved in H_2_O/D_2_O at room temperature. The peaks corresponding to structurally and hence magnetically inequivalent carbon atoms are labeled accordingly. Color code: MoO_6_, violet octahedra; As, green; C, yellow; O, red; H, black.

### UV–Vis Spectroscopy

The stability of the bioactive polyanions **C_6_H_5_AsMo_6_**, **4-F_3_COC_6_H_4_AsMo_6_**, and **4-BrC_6_H_4_AsMo_6_** was further evaluated by UV–vis spectroscopy (Figures S35)_._ Three absorption bands were observed at 200, 230–254, and 320 nm. The 200 nm band corresponds to the pπ–dπ charge transfer transition of the O_t_ → Mo, while the 230–254 nm band arises from O_b,c_ → Mo charge transfer and π–π* transitions within the aromatic ring. The intense 320 nm absorption signifies ligand-to-metal charge transfer (LMCT), involving electron transfer from the filled π-orbitals of surrounding oxygens to the vacant d orbitals of Mo^5+^ centers. The unchanged position and intensity of these bands suggest that no significant structural transformations occurred.

### ESI Mass Spectrometry

ESI mass spectra of the polyanions were acquired from aqueous solution in both positive and negative ion mode. In positive ion mode, complex spectra with multiple Mo_*x*_ species could be observed. In most cases, the intact polyanion corresponded to the dominant signal. Four such examples are shown in the Supporting Information (Figures S36–S39).

In negative ion mode, all compounds analyzed showed high-quality ESI-MS spectra with a systematic pattern. All compounds showed two strong cluster of ions, a first between *m*/*z* 680–750 and a second between *m*/*z* 1380–1540. For a detailed discussion we selected two representative examples **C_6_H_5_AsMo_6_** and **4-FC_6_H_4_AsMo_6_**. All mass spectrometry data are summarized in Table S13.

The polyanion **C_6_H_5_AsMo_6_** showed a first cluster of signals at *m*/*z* 714.55 and a second cluster at *m*/*z* 1452.09 (Figure 4). The first cluster at *m/*z 714.55 was assigned to a doubly charged negative ion with an elemental composition of [Mo_6_As_4_C_12_H_26_O_24_]^2–^. The observed isotope pattern is in full agreement with the structure as demonstrated by comparison of the experimental spectrum to a simulation of the spectrum. The second cluster at *m*/*z* 1452.09 was assigned to originate from two individual species in the gas phase. First, a monomeric singly charged species with an elemental composition of [NaMo_6_As_4_C_12_H_26_O_24_]^−^ with a mass difference of 1 *m*/*z* between isotope peaks. Second, in between these signals a second species with isotope differences of 0.5 *m*/*z* was observed, which was assigned to a dimeric species with an elemental composition of [NaMo_6_As_4_C_12_H_26_O_24_]_2_^2–^.

**Figure 4:**
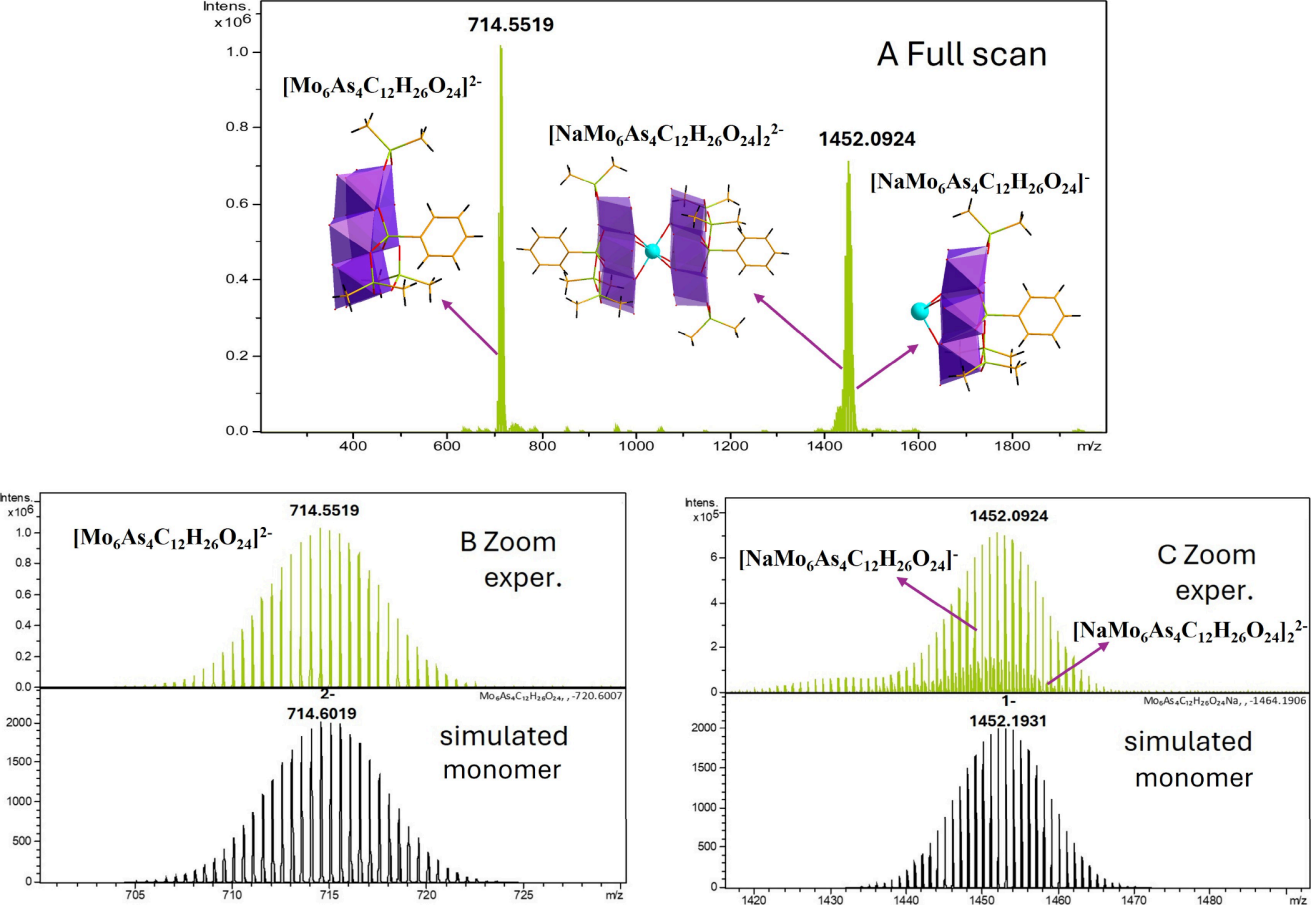
Negative mode ESI mass spectra of compound **C_6_H_5_AsMo_6_**. (A) Full-scan full-range view with two main signal clusters, (B) expanded region around *m*/*z* 714 with experimental spectra in the upper panel and a simulated isotope pattern in the bottom panel, and (C) expanded region around *m*/*z* 1452 with experimental spectra in the upper panel (please note signals of dimeric species at a 0.5 *m*/*z* mass difference at lower intensity in between main signals) and simulated isotope pattern in the bottom panel.

Again, simulated spectra are in full agreement with experimental spectra (see Figure 5 for **4-FC_6_H_4_AsMo_6_** as an example). As demonstrated from single-crystal X-ray structural data, the compound as well dimerizes in the gas phase with a central Na ion, reminiscent of a 15-crown-5 crown ether. From the relative ion intensities of the two monomeric species and the single dimeric species, a ratio of monomer to dimer of 3.1 was determined. When comparing monomer to dimer ratios, it appears that electron-withdrawing substituents on the *para*-position of the aromatic moiety attached to As favor and stabilize the dimeric species in the gas phase.

**Figure 5:**
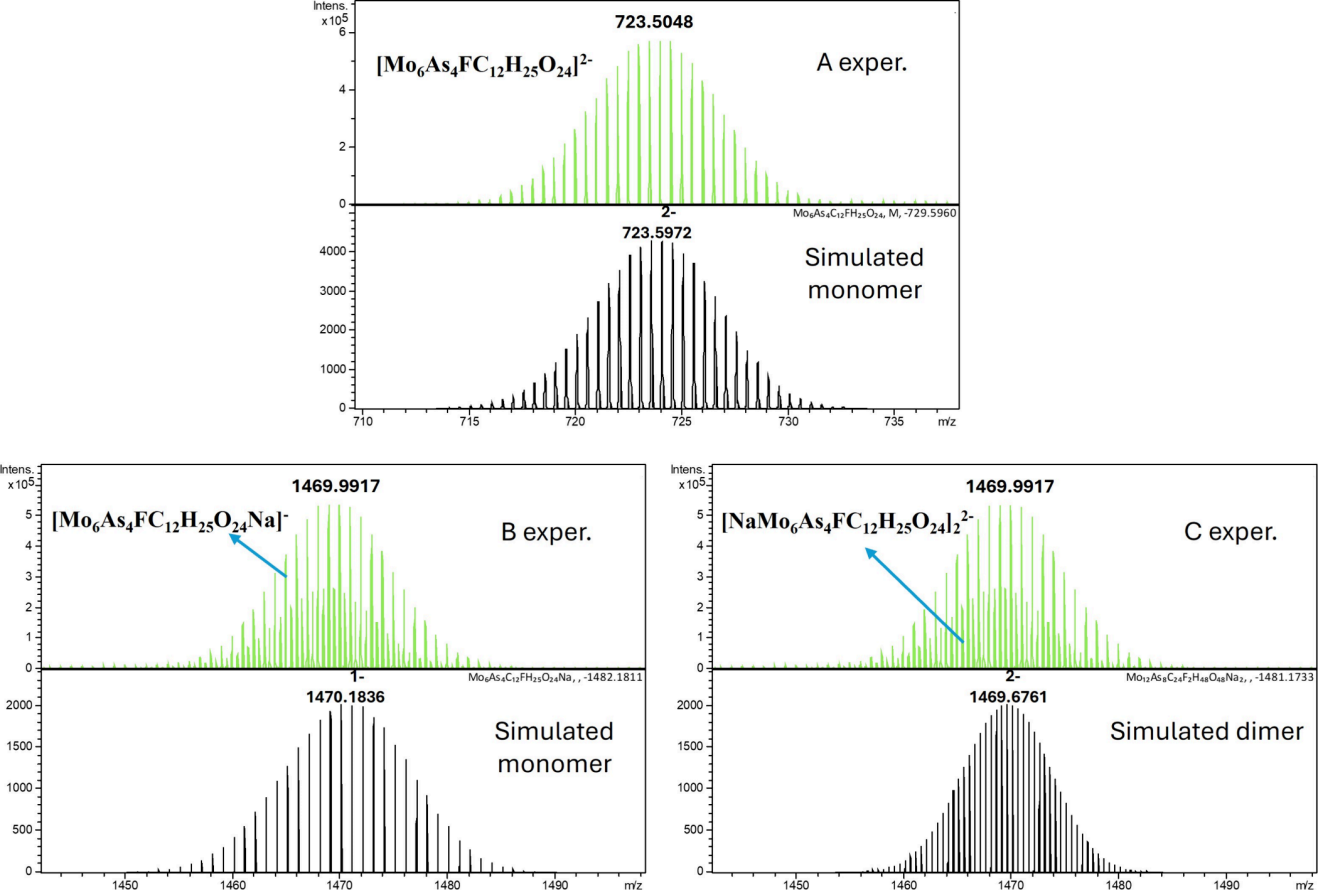
Experimental (upper panel) and simulated (bottom panel) mass spectra of **4-FC_6_H_4_AsMo_6_** at *m*/*z* 723 and 1469. Panel A shows simulation of doubly charged monomeric species [Mo_6_As_4_FC_12_H_25_O_24_]^2–^; panel B shows singly charged monomeric species [Mo_6_As_4_FC_12_H_25_O_24_Na]^−^, and panel C shows simulation of doubly charged dimeric species [NaMo_6_As_4_FC_12_H_25_O_24_]_2_^2–^.

The ESI spectra are reminiscent of related phosphorus-containing analogues reported earlier.^4^ In three samples containing a 4-trifluoromethoxy, 4-bromo, or azido substituent on an aromatic moiety, an additional cluster of signals could be observed with an additional mass increment of 125 *m*/*z* (Figures S40–S42). We tentatively assign this mass difference to added water molecules.

### Tandem Mass Spectrometry

Due to the stability of the compounds in the solution and in the gas-phase, tandem MS experiments could be carried out. The **C_6_H_5_AsMo_6_** ions were isolated in the quadrupole with an isolation width of 20 *m*/*z* and fragmented at a collision energy of 50 eV. Fragment spectra of monomeric doubly charged species at *m*/*z* 714 (Figure 4) and singly charged species at *m*/*z* 1452 as precursor ions were almost identical. Key fragment ion clusters could be observed centered at *m*/*z* 719.4, 575.2, 431.6. The same fragment ions could be observed for another compound subjected to tandem MS analysis (see Figure S43).

By comparison of experimental and simulated mass spectra, the fragment ions were assigned as [Mo_5_O_15_H_4_]^−^ at *m*/*z* 719, [C_6_H_5_AsMo_6_O_17_(OH)­{AsO_2_(CH_3_)_2_}]^2–^ at *m*/*z* 575, and [C_6_H_5_AsMo_6_O_17_(OH)­{AsO_2_(CH_3_)_2_}_2_]^3–^ at *m*/*z* 431. Consequently, the polyanions loose in the gas phase up to two dimethylarsinate ligands and in addition the central arsenic moiety to yield stable Mo_*x*_O_3*x*_ species (Figure 6).

**Figure 6:**
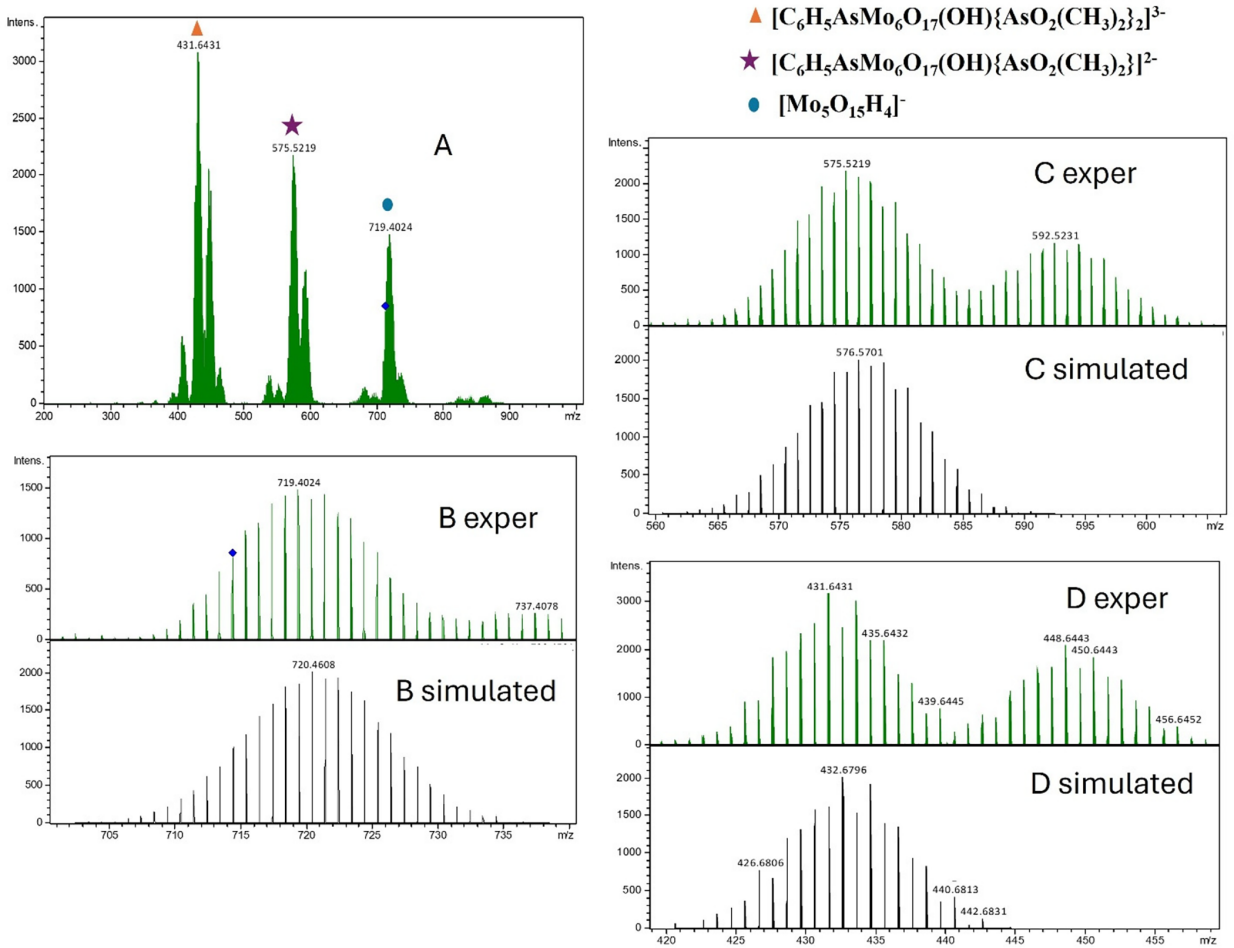
(A) Tandem MS spectra in negative mode of **C_6_H_5_AsMo_6_** with the precursor ion at *m*/*z* 714. Panel B shows experimental and simulated spectra of the fragment ion centered around *m*/*z* 719, panel C of the experimental and simulated fragment ion at *m*/*z* 575, and panel D experimental and simulated spectra at *m*/*z* 431.

### Biological Activity Determination of Minimal Inhibitory Concentrations (MIC)

The antimicrobial efficacy of organoarsonate-functionalized polyoxomolybdates was assessed against the Gram-negative bacteria *Escherichia coli* (DSM 6897, grown in LB Medium), *Vibrio parahaemolyticus* (grown in LB Medium) and *Salmonella enterica* (DSM 19587, grown in LB Medium) as well as the Gram-positive bacteria *Bacillus subtilis* (DSM 1088, grown in TSY Medium) and *Listeria monocytogenes* (DSM 15675, grown in LB Medium).

All tested POMs exhibited MIC values exceeding 500 μg/mL against all Gram-negative bacteria. Only the POM **4-BrC_6_H_4_AsMo_6_** gave weak activity against *V. parahaemolyticus* (250 μg/mL) and *S. enterica* (500 μg/mL), indicating an overall negligible antibacterial activity (Table 1). This suggests that the outer membrane of Gram-negative bacteria is a strong permeability barrier, limiting the intracellular accumulation of these large, charged molecules. Similar findings have been reported for other polyoxometalates, which generally demonstrate low efficacy against Gram-negative bacteria due to their structural properties.^32^

**Table 1:** Minimum Inhibitory Concentration (MIC) Values (μg/mL) of the Polyanions Reported Here against Three Different Gram-Negative and Two Gram-Positive Bacteria

**microorganism**		**C**_**6**_ **H**_**5**_ **AsMo**_**6**_	**4-F**_**3**_ **COC**_**6**_ **H**_**4**_ **AsMo**_**6**_	**4-BrC**_**6**_ **H**_**4**_ **AsMo**_**6**_	other POMs^a^
*Escherichia coli*	Gram-negative	>500	>500	>500	>500
*Vibrio parahaemolyticus*	Gram-negative	>500	>500	250	>500
*Salmonella enterica*	Gram-negative	>500	>500	500	>500
*Bacillus subtilis*	Gram-positive	250	62.5–125	125	>500
*Listeria monocytogenes*	Gram-positive	>500	303	40	>500

a Other POMs are **As^V^Mo_6_**, **CH_3_AsMo_6_**, **4-FC_6_H_4_AsMo_6_**, **4-F_3_CC_6_H_4_AsMo_6_**, **4-N_3_C_6_H_4_AsMo_6_**, and **H_2_O_4_C_2_C_6_H_3_AsMo_6_**.

However, certain POMs with aromatic or halogenated phenyl groups (e.g., **NaNH_4_–C_6_H_5_AsMo_6_**, **NaNH_4_–F_3_COC_6_H_4_AsMo_6_**, and **NaNH_4_–BrC_6_H_4_AsMo_6_**) demonstrate moderate activity against the Gram-positive bacteria *B. subtilis* and *L. monocytogenes*, with MICs ranging from 62.5 to 250 μg/mL and 40–303 μg/mL, respectively. The agar diffusion plates confirm the activity of **4-BrC_6_H_4_AsMo_6_** as the best sample with the highest clear zone of inhibition against the pathogen *Listeria monocytogenes* (Figure S44). This activity may be attributed to the lack of an outer membrane compared to Gram-negative bacteria, allowing for easier cell wall penetration^33^ and showing a clear distinction in susceptibility between these bacterial types. MIC values of the control gentamycin lie in the general ranges for all bacterial strains.^34^

Previous studies have demonstrated that halogenation can enhance bioactivity by altering compound–membrane interactions and improving uptake.^35^ All active compounds have a phenyl ring, which supports the findings that the phenyl ring serves as a structural backbone and the antimicrobial activity depends on the substituents.^36^ Among the tested compounds, **4-BrC_6_H_4_AsMo_6_** exhibited the highest antibacterial activity, likely due to the effect of bromine substitution on the polyanion structure (Figure S44). Control studies using the reference ligands sodium cacodylate and BrC_6_H_4_AsO_3_H_2_ against *Listeria monocytogenes* showed MIC values of >1000 and 62.5 μg/mL, respectively. This indicates that while the BrC_6_H_4_AsO_3_H_2_ ligand alone exhibits moderate activity, the corresponding polyanion **4-BrC_6_H_4_AsMo_6_** displays enhanced efficacy with an MIC of 40 μg/mL. Hence, this could lead to medicinal relevance in combating bacterial diseases for both Gram-positive and Gram-negative pathogenic microbes, although further structure–activity relationship (SAR) studies are necessary to elucidate the underlying mechanisms.

## Conclusions

In summary, we have conducted a comprehensive investigation into the synthesis and characterization of a novel family of water-soluble, stable, and organically-functionalized, reduced polyoxomolybdates. Eleven dimethylarsinate-functionalized arsenomolybdates­(V), [RAsMo^V^_6_O_15_(OH)_3_{AsO_2_(CH_3_)_2_}_3_]^2–^ (R = HO, CH_3_, C_2_H_5_, C_6_H_5_, 3,5-(HOOC)_2_C_6_H_3_, 4-FC_6_H_4_, 4-F_3_CC_6_H_4_, 4-F_3_COC_6_H_4_, 4-BrC_6_H_4_, and 4-N_3_C_6_H_4_) and [As^III^Mo^V^_6_O_15_(OH)_3_{AsO_2_(CH_3_)_2_}_3_]^3–^, were successfully isolated via a straightforward one-pot aqueous synthesis under ambient, open-beaker conditions. Fine-tuning of reagent stoichiometry, ionic strength, and most critically, pH was essential to achieve selective formation of the target species. The incorporation of structurally diverse organoarsonate groups, including fluorinated, brominated, and carboxylated substituents, within a robust hexamolybdate­(V) core provided a versatile platform to examine solubility and solution stability near physiological pH. Detailed multinuclear NMR (^1^H, ^19^F, and ^13^C) spectroscopy confirmed their structural integrity in solution, while ESI-MS revealed the coexistence of both monomeric and dimeric species. Moreover, tandem MS (CID) enabled stepwise fragmentation studies of the peripherally bound dimethylarsinate ligands, offering insights into the gas-phase behavior of these complex assemblies. Given the tunable organic functionality, these polyanions present exciting opportunities for biomolecular interaction studies. Preliminary biological assays revealed that the three derivatives **C_6_H_5_AsMo_6_**, **4-F_3_COC_6_H_4_AsMo_6_**, and **4-BrC_6_H_4_AsMo_6_** exhibit moderate antibacterial activity, suggesting some potential as functional POM-based antimicrobial agents.

## Supplementary Material



## Notes

Single-crystal XRD data, bond valence sum calculations, FT-IR spectra, thermograms, multinuclear NMR spectra (^1^H, ^19^F, and ^13^C), ESI-MS data and spectra, tandem MS spectrum, UV–vis spectra, agar diffusion test, and MIC plate images (PDF)
