# Supplementary material for: Organoarsonate- and Dimethylarsinate-Functionalized Hexamolybdates(V): A Multifaceted Study on Synthesis, Structural Dynamics, and Antibacterial Properties
Source: Inorg Chem. 2025 Aug 25;64(35):17890–903. doi: 10.1021/acs.inorgchem.5c02852 (PMC12421668; doi:10.1021/acs.inorgchem.5c02852)
Supplement: Supplementary file 1 [file ic5c02852_si_001.pdf]

Associated Content

Supporting Information

Organoarsonate- and Dimethylarsinate-Functionalized Hexamolybdates(V): A Multifaceted Study on Synthesis, Structural Dynamics, and Antibacterial Properties

Vinaya Siby,[a] Arun Pal,[a] Anupam Sarkar,[a] Bassem S. Bassil,[a] Anneke Immoor,[a] James Ziemah,[a] Jana Hölscher,[a] Levente Kiss,[b] Cristian Silvestru,[b] Matthias S. Ullrich,[a] Nikolai Kuhnert,[a] Ulrich Kortz*[a]

[a]*School of Science, Constructor University, Campus Ring 1, 28759 Bremen, Germany,* Email: ukortz@constructor.university

[b]*Department of Chemistry, Supramolecular Organic and Organometallic Chemistry Centre (SOOMCC), Faculty of Chemistry and Chemical Engineering, Babeş-Bolyai University, 11 Arany Janos, 400028 Cluj-Napoca, Romania*

Table S1. Crystal data and structure refinement for **$NaNH_4$-$As^{III}Mo_6$**, **$NaNH_4$-$As^{V}Mo_6$**, **$NaNH_4$-CH_3AsMo_6**, **$NaNH_4$-$C_2H_5AsMo_6$**, **$NaNH_4$-$C_6H_5AsMo_6$**, **$NaNH_4$-$FC_6H_4AsMo_6$**, **$NaNH_4$-$F_3CC_6H_4AsMo_6$**, **$NaNH_4$-$F_3COC_6H_4AsMo_6$**, **$NaNH_4$-$BrC_6H_4AsMo_6$**, **$NaNH_4$-$N_3C_6H_4AsMo_6$** and **$NaNH_4$-$H_2O_4C_2C_6H_3AsMo_6$.**

compound	**$NaNH_4$-$As^{III}Mo_6$**	**$NaNH_4$-$As^{V}Mo_6$**	**$NaNH_4$-CH_3AsMo_6**	**$NaNH_4$-$C_2H_5AsMo_6$**	**$NaNH_4$-$C_6H_5AsMo_6$**	**$NaNH_4$-$FC_6H_4AsMo_6$**	**$NaNH_4$-$F_3CC_6H_4AsMo_6$**	**$NaNH_4$-$F_3COC_6H_4AsMo_6$**	**$NaNH_4$-$BrC_6H_4AsMo_6$**	**$NaNH_4$-$N_3C_6H_4AsMo_6$**	**$NaNH_4$-$H_2O_4C_2C_6H_3AsMo_6$**
empirical formula[a]	$C_{12}H_{36}As_8Mo_{12}NaO_{48}$ ($C_6H_{45}As_4Mo_6N_{0.5}Na_{2.5}O_{35}$)	$C_{12}H_{36}As_8Mo_{12}NaO_{50}$ ($C_6H_{46.4}As_4Cl_{0.6}Mo_6N_{1.1}Na_{1.5}O_{35}$)	$C_{14}H_{42}As_8Mo_{12}NaO_{48}$ ($C_7H_{48}As_4Mo_6NNaO_{34}$)	$C_{16}H_{36}As_8Mo_{12}NaO_{48}$ ($C_8H_{48}As_4Mo_6N_{0.5}Na_{1.5}O_{34}$)	$C_{24}H_{44}As_8Mo_{12}NaO_{48}$ ($C_{12}H_{50}As_4Mo_6NNaO_{34}$)	$C_{24}H_{44}As_8F_2Mo_{12}NaO_{48}$ ($C_{12}H_{43}As_4FMo_6NNaO_{31}$)	$C_{26}H_{44}As_8F_6Mo_{12}NaO_{48}$ ($C_{13}H_{51}As_4F_3Mo_6NNaO_{35}$)	$C_{26}H_{44}As_8F_6Mo_{12}NaO_{50}$ ($C_{13}H_{49}As_4F_3Mo_6NNaO_{35}$)	$C_{24}H_{44}As_8Br_2Mo_{12}NaO_{48}$ ($C_{12}H_{46.2}As_4BrMo_6N_{1.3}Na_{0.7}O_{32}$)	$C_{24}H_{44}As_8Mo_{12}N_6NaO_{48}$ ($C_{12}H_{47.8}As_4Mo_6N_{3.7}Na_{1.3}O_{34}$)	$C_{28}H_{43}As_8Mo_{12}NaO_{56}$ ($C_{14}H_{58}As_4Mo_6NNaO_{42}$)
fw, g mol^{-1}	2722.04	2754.04	2752.10	2770.08	2874.22	2912.22	3012.24	3044.24	3034.04	2958.28	3050.26
wavelength, λ	0.71073 Å	0.71073 Å	0.71073 Å	0.71073 Å	0.71073 Å	1.54184 Å	1.54184 Å	1.54184 Å	0.71073 Å	1.54184 Å	0.71073 Å
cryst syst	orthorhombic	trigonal	trigonal	trigonal	monoclinic	monoclinic	triclinic	triclinic	triclinic	triclinic	triclinic
space group	Cmce	R-3	R-3	R-3	C2/c	C2/c	P-1	P-1	P-1	P-1	P-1
a, Å	21.3273(4)	19.5888(5)	19.4936(6)	19.6450(3)	17.1895(2)	17.07130(10)	12.99953(9)	13.12451(11)	13.0878(13)	13.11405(19)	13.0085(2)
b, Å	13.3913(2)	19.5888(5)	19.4936(6)	19.6450(3)	19.6644(2)	19.75620(10)	13.32682(9)	13.25955(14)	13.1251(13)	13.44511(17)	13.0168(2)
c, Å	30.0798(5)	19.1897(6)	19.2110(8)	19.0944(3)	30.8943(5)	30.4721(2)	17.01712(9)	17.18372(16)	17.2923(17)	16.93935(17)	16.6003(3)
α, deg	90	90	90	90	90	90	105.2840(5)	105.7332(9)	108.865(2)	110.1182(10)	107.8400(10)
β, deg	90	90	90	90	103.7160(10)	102.9450(10)	99.2811(5)	98.4231(7)	96.563(3)	99.2863(11)	94.1380(10)
γ, deg	90	120	120	120	90	90	100.4196(6)	100.3361(8)	99.213(2)	101.2768(12)	97.5760(10)
volume, $Å^3$	8590.8(3)	6377.0(4)	6322.1(5)	6381.8(2)	10145.1(2)	10015.95(11)	2728.24(3)	2770.88(5)	2730.4(5)	2664.24(6)	2633.46(8)
Z	4	3	3	3	4	4	1	1	1	1	1
D_{calc}, g cm^{-3}	2.105	2.151	2.169	2.162	1.882	1.931	1.833	1.824	1.845	1.844	1.923
abs coeff, mm^{-1}	4.824	4.877	4.917	4.872	4.090	15.513	14.317	14.119	4.532	14.581	3.951
F(000)	5084.0	3861.0	3867.0	3885.0	5404.0	5476.0	1417.0	1433.0	1421.0	1393.0	1439.0
2Θ range for data collection, deg	4.682 to 67.574	7.61 to 54.826	7.242 to 61.004	6.402 to 67.516	4.554 to 67.704	5.952 to 160.18	5.52 to 160.758	5.46 to 160.192	4.86 to 57.35	5.736 to 165.892	4.89 to 49.424

completeness to Θ_{max}, %	92.3	99.2	99.8	92.7	92.2	98.6	98.5	98.7	98.1	96.4	100
index ranges	-31 ≤ h ≤ 32, -20 ≤ k ≤ 20, -46 ≤ l ≤ 46	-17 ≤ h ≤ 22, -25 ≤ k ≤ 24, -20 ≤ l ≤ 24	-27 ≤ h ≤ 27, -26 ≤ k ≤ 27, -27 ≤ l ≤ 26	-30 ≤ h ≤ 29, -28 ≤ k ≤ 29, -28 ≤ l ≤ 28	-26 ≤ h ≤ 26, -29 ≤ k ≤ 30, -47 ≤ l ≤ 44	-21 ≤ h ≤ 21, -24 ≤ k ≤ 25, -38 ≤ l ≤ 36	-16 ≤ h ≤ 16, -17 ≤ k ≤ 16, -21 ≤ l ≤ 21	-16 ≤ h ≤ 16, -15 ≤ k ≤ 16, -21 ≤ l ≤ 21	-17 ≤ h ≤ 15, -17 ≤ k ≤ 17, -21 ≤ l ≤ 23	-16 ≤ h ≤ 16, -16 ≤ k ≤ 17, -21 ≤ l ≤ 21	-15 ≤ h ≤ 15, -15 ≤ k ≤ 15, -19 ≤ l ≤ 19
reflns collected	79486	12376	44964	46392	170289	118801	169390	186551	39052	133732	126875
indep reflns	8144	3212	4293	5269	18891	10804	11755	11956	13819	11478	8976
R(int)	0.0510	0.0463	0.0987	0.0543	0.0600	0.0373	0.0564	0.0656	0.1033	0.0678	0.0440
data/restraints/param	8144/0/201	3212/0/126	4293/0/130	5269/103/135	18891/288/435	10804/519/436	11755/0/463	11956/554/472	13819/291/430	11478/535/454	8976/0/481
GOF on F^2	1.094	1.038	0.871	1.042	1.071	1.042	1.038	0.991	1.030	1.032	1.071
R_1,[b] wR_2[c] [$I > 2\sigma(I)$]	R_1 = 0.0240, wR_2 = 0.0592	R_1 = 0.0205, wR_2 = 0.0550	R_1 = 0.0285, wR_2 = 0.0642	R_1 = 0.0267, wR_2 = 0.0617	R_1 = 0.0479, wR_2 = 0.1055	R_1 = 0.0575, wR_2 = 0.1441	R_1 = 0.0330, wR_2 = 0.0916	R_1 = 0.0440, wR_2 = 0.1195	R_1 = 0.0624, wR_2 = 0.1620	R_1 = 0.0563, wR_2 = 0.1688	R_1 = 0.0385, wR_2 = 0.0961
R_1,[b] wR_2[c] (all data)	R_1 = 0.0333, wR_2 = 0.0609	R_1 = 0.0221, wR_2 = 0.0556	R_1 = 0.0397, wR_2 = 0.0685	R_1 = 0.0346, wR_2 = 0.0635	R_1 = 0.0603, wR_2 = 0.1091	R_1 = 0.0602, wR_2 = 0.1464	R_1 = 0.0362, wR_2 = 0.0942	R_1 = 0.0472, wR_2 = 0.1231	R_1 = 0.0984, wR_2 = 0.1817	R_1 = 0.0615, wR_2 = 0.1776	R_1 = 0.0398, wR_2 = 0.0969
largest diff peak and hole, e Å^{-3}	1.60/-0.77	0.87/-0.79	1.06/-0.83	1.02/-0.71	1.37/-1.12	6.46/-6.97	1.12/-0.95	1.82/-1.06	4.29/-2.02	3.46/-1.58	2.98/-0.65

[a] The entries in the parentheses represent the actual formula units as obtained from bulk elemental analysis. [b] $R_1 = \Sigma||F_o| - |F_c||/\Sigma|F_o|$. [c] $wR_2 = [\Sigma w(F_o^2 - F_c^2)^2/\Sigma w(F_o^2)^2]^{1/2}$.

Table S2. Bond valence sum (BVS) values for **$NaNH_4$-HOAsMo$_6$**. Bond Valence (v_{ij}) = exp[(R_{ij}-d_{ij})/b (R_{ij} = bond valence parameter; b = 0.37); Bond Valence Sum (V_i) = $\Sigma_j v_{ij}$.

i	j	d_{ij} (Å)	v_{ij}	i	j	d_{ij} (Å)	v_{ij}
Mo1	O2	1.697	1.763971	Mo2	O6	1.6901	1.797175
	O4	1.9405	0.913437		O4	1.9387	0.917892
	O5	1.9425	0.908513		O5	1.9516	0.886441
	O8	2.1161	0.568283		O7	2.0842	0.619453
	O1	2.122	0.559293		O1	2.1329	0.543057
	O3	2.2885	0.356621		O3	2.2896	0.355563
			**5.070119**				**5.119581**
O1	Mo1	2.122	0.559293	O9	As1	1.6887	**1.23568**
	Mo2	2.1329	0.543057				
			**1.102351**				

Table S3. Bond valence sum (BVS) values for **$NaNH_4$-As^{III}Mo$_6$**. Bond Valence (v_{ij}) = exp[(R_{ij}-d_{ij})/b (R_{ij} = bond valence parameter; b = 0.37); Bond Valence Sum (V_i) = $\Sigma_j v_{ij}$.

i	j	d_{ij} (Å)	v_{ij}	i	j	d_{ij} (Å)	v_{ij}
Mo1	O1	1.6971	1.763494	Mo2	O7	1.6933	1.781699
	O6	1.9405	0.913437		O6	1.9411	0.911957
	O5	1.9434	0.906306		O5	1.9463	0.89923
	O3	2.1052	0.585274		O9	2.1185	0.564609
	O2	2.1368	0.537363		O8	2.1284	0.549702
	O4	2.2055	0.446303		O14	2.2089	0.442221
			**5.152178**				**5.149419**
Mo3	O11	1.6923	1.786521	As1			
	O12	1.9312	0.936688		O14	1.7778	0.971233
	O10	1.9465	0.898744		O4	1.7883	0.944058
	O13	2.0888	0.611799		O14	1.7778	0.971233
	O9	2.1218	0.559596				**2.886524**
	O14	2.2279	0.420085				
			**5.213433**				
O2	Mo1	2.1368	0.537363	O9	Mo2	2.1185	0.564609
	Mo1	2.1368	0.537363		Mo3	2.1218	0.559596
			**1.074726**				**1.124205**

Table S4. Bond valence sum (BVS) values for **$NaNH_4$-CH_3AsMo$_6$**. Bond Valence (v_{ij}) = exp[(R_{ij}-d_{ij})/b (R_{ij} = bond valence parameter; b = 0.37); Bond Valence Sum (V_i) = $\Sigma_j v_{ij}$.

i	j	d_{ij} (Å)	v_{ij}	i	j	d_{ij} (Å)	v_{ij}
Mo1	O2	1.6908	1.793779	Mo2	O5	1.6842	1.826063
	O3	1.9326	0.93315		O3	1.9346	0.92812
	O4	1.9419	0.909988		O4	1.9456	0.900933
	O8	2.1012	0.591635		O7	2.0749	0.63522
	O6	2.1235	0.557031		O6	2.1304	0.546739
	O1	2.2955	0.349938		O1	2.2865	0.358554
			**5.13552**				**5.195629**
O1	Mo1	2.1235	0.557031				
	Mo2	2.1304	0.546739				
			**1.10377**				

Table S5. Bond valence sum (BVS) values for **$NaNH_4$-$C_2H_5AsMo_6$**. Bond Valence (v_{ij}) = exp[(R_{ij}-d_{ij})/b (R_{ij} = bond valence parameter; b = 0.37); Bond Valence Sum (V_i) = $\Sigma_j v_{ij}$.

i	j	d_{ij} (Å)	v_{ij}	i	j	d_{ij} (Å)	v_{ij}
Mo1	O3	1.6882	1.806428	Mo2	O6	1.693	1.783144
	O4	1.9374	0.921123		O4	1.9361	0.924365
	O5	1.9499	0.890523		O5	1.9425	0.908513
	O2	2.0769	0.631796		O8	2.1008	0.592275
	O7	2.1297	0.547774		O7	2.1148	0.570284
	O1	2.283	0.361962		O1	2.2877	0.357393
			**5.159606**				**5.135974**
O7	Mo2	2.1148	0.570284				
	Mo1	2.1297	0.547774				
			**1.118058**				

Table S6. Bond valence sum (BVS) values for **$NaNH_4$-$C_6H_5AsMo_6$**. Bond Valence (v_{ij}) = exp[(R_{ij}-d_{ij})/b (R_{ij} = bond valence parameter; b = 0.37); Bond Valence Sum (V_i) = $\Sigma_j v_{ij}$.

i	j	d_{ij} (Å)	v_{ij}	i	j	d_{ij} (Å)	v_{ij}
Mo1	O1	1.69	1.797661	Mo2	O7	1.6841	1.826556
	O5	1.929	0.942274		O5	1.9367	0.922867
	O6	1.9499	0.890523		O6	1.9578	0.871711
	O2	2.0978	0.597097		O8	2.0626	0.656692
	O3	2.1205	0.561565		O9	2.1359	0.538672
	O4	2.2946	0.35079		O10	2.2948	0.3506
			**5.139911**				**5.167098**
Mo3	O12	1.6991	1.753988	Mo4	O15	1.7055	1.723909
	O13	1.9416	0.910726		O14	1.9281	0.944569
	O14	1.9485	0.893899		O13	1.9408	0.912697
	O11	2.08	0.626524		O16	2.0856	0.617113
	O9	2.1128	0.573374		O17	2.1238	0.556579
	O10	2.2755	0.369374		O18	2.299	0.346643
			**5.127885**				**5.10151**
Mo5	O20	1.7071	1.716471	Mo6	O23	1.6927	1.784591
	O22	1.9265	0.948662		O21	1.9371	0.92187
	O21	1.9328	0.932646		O22	1.9453	0.901664
	O19	2.111	0.576171		O24	2.066	0.650685
	O17	2.1113	0.575704		O3	2.1323	0.543939
	O18	2.3027	0.343194		O4	2.2769	0.367979
			**5.092847**				**5.170727**
O9	Mo3	2.1128	0.573374	O17	Mo5	2.1113	0.575704
	Mo2	2.1359	0.538672		Mo4	2.1238	0.556579
			**1.112046**				**1.132283**
O3	Mo1	2.1205	0.561565				
	Mo6	2.1323	0.543939				
			**1.105504**				

Table S7. Bond valence sum (BVS) values for **$NaNH_4$-$FC_6H_4AsMo_6$**. Bond Valence (v_{ij}) = exp[(R_{ij}-d_{ij})/b (R_{ij} = bond valence parameter; b = 0.37); Bond Valence Sum (V_i) = $\Sigma_j v_{ij}$.

i	j	d_{ij} (Å)	v_{ij}	i	j	d_{ij} (Å)	v_{ij}
Mo1	O1	1.6862	1.816219	Mo2	O8	1.6876	1.80936
	O2	1.934	0.929626		O9	1.9432	0.906796
	O3	1.9568	0.87407		O10	1.9505	0.88908
	O6	2.0788	0.62856		O7	2.0828	0.621801
	O5	2.1351	0.539838		O5	2.1132	0.572755
	O4	2.3017	0.344123		O4	2.2848	0.360205
			**5.132435**				**5.159997**
Mo3	O11	1.6822	1.83596	Mo4	O16	1.6932	1.782181
	O10	1.9372	0.921621		O18	1.942	0.909742
	O9	1.9461	0.899716		O17	1.9436	0.905816
	O12	2.0883	0.612626		O15	2.0965	0.599199
	O13	2.1211	0.560655		O13	2.1154	0.569359
	O14	2.2946	0.35079		O14	2.3115	0.335128
			**5.181369**				**5.101425**
Mo5	O19	1.6846	1.82409	Mo6	O24	1.6956	1.770658
	O17	1.9488	0.893175		O2	1.937	0.922119
	O18	1.9513	0.88716		O3	1.9545	0.879521
	O20	2.0626	0.656692		O23	2.0982	0.596452
	O22	2.1347	0.540422		O22	2.1214	0.560201
	O21	2.2797	0.365205		O21	2.2948	0.3506
			**5.166743**				**5.079551**
O5	Mo2	2.1132	0.572755	O13	Mo4	2.1154	0.569359
	Mo1	2.1351	0.539838		Mo3	2.1211	0.560655
			**1.112593**				**1.130015**
O22	Mo6	2.1214	0.560201				
	Mo5	2.1347	0.540422				
			**1.100623**				

Table S8. Bond valence sum (BVS) values for **$NaNH_4$-$F_3CC_6H_4AsMo_6$**. Bond Valence (v_{ij}) = exp[(R_{ij}-d_{ij})/b (R_{ij} = bond valence parameter; b = 0.37); Bond Valence Sum (V_i) = $\Sigma_j v_{ij}$.

i	j	d_{ij} (Å)	v_{ij}	i	j	d_{ij} (Å)	v_{ij}
Mo1	O1	1.6865	1.814747	Mo2	O8	1.6847	1.823597
	O2	1.9506	0.88884		O9	1.9501	0.890042
	O3	1.9542	0.880234		O10	1.9521	0.885244
	O5	2.0756	0.634019		O7	2.0766	0.632308
	O6	2.1132	0.572755		O6	2.1294	0.548219
	O4	2.2807	0.364219		O4	2.2911	0.354124
			**5.154814**				**5.133534**
Mo3	O11	1.6842	1.826063	Mo4	O16	1.6823	1.835464
	O9	1.9483	0.894383		O17	1.938	0.91963
	O10	1.9563	0.875252		O18	1.957	0.873598
	O12	2.083	0.621465		O15	2.0822	0.62281
	O13	2.1032	0.588446		O13	2.1249	0.554927
	O14	2.3512	0.301031		O14	2.2966	0.348899
			**5.10664**				**5.155328**
Mo5	O19	1.6838	1.828038	Mo6	O24	1.6848	1.823104
	O18	1.9398	0.915167		O3	1.9465	0.898744
	O17	1.9471	0.897288		O2	1.9489	0.892933
	O20	2.0976	0.59742		O23	2.0595	0.662217
	O22	2.1424	0.529291		O22	2.1317	0.544821

	O21	2.294	0.351359		O21	2.3048	0.341252
			**5.118564**				**5.163072**
O6	Mo1	2.1132	0.572755	O13	Mo3	2.1032	0.588446
	Mo2	2.1294	0.548219		Mo4	2.1249	0.554927
			**1.120974**				**1.143373**
O22	Mo6	2.1317	0.544821				
	Mo5	2.1424	0.529291				
			**1.074113**				

Table S9. Bond valence sum (BVS) values for **$NaNH_4$-$F_3COC_6H_4AsMo_6$**. Bond Valence (v_{ij}) = exp[(R_{ij}-d_{ij})/b (R_{ij} = bond valence parameter; b = 0.37); Bond Valence Sum (V_i) = $\Sigma_j v_{ij}$.

i	j	d_{ij} (Å)	v_{ij}	i	j	d_{ij} (Å)	v_{ij}
Mo1	O1	1.6789	1.852408	Mo2	O8	1.688	1.807405
	O2	1.9416	0.910726		O9	1.9428	0.907777
	O3	1.9421	0.909496		O10	1.9449	0.902639
	O4	2.1008	0.592275		O7	2.0535	0.673043
	O5	2.1392	0.533889		O5	2.1291	0.548663
	O6	2.3019	0.343937		O6	2.3127	0.334043
			**5.142731**				**5.17357**
Mo3	O11	1.6827	1.833481	Mo4	O16	1.6852	1.821134
	O9	1.949	0.892692		O17	1.9424	0.908759
	O10	1.9561	0.875725		O18	1.9553	0.877621
	O12	2.0768	0.631966		O15	2.0874	0.614118
	O13	2.1185	0.564609		O13	2.1187	0.564304
	O14	2.2842	0.36079		O14	2.3049	0.341159
			**5.159264**				**5.127096**
Mo5	O19	1.6819	1.837449	Mo6	O24	1.6804	1.844914
	O17	1.9454	0.90142		O2	1.9404	0.913684
	O18	1.9507	0.8886		O3	1.9558	0.876436
	O20	2.0863	0.615947		O23	2.0839	0.619955
	O22	2.1033	0.588287		O22	2.1312	0.545558
	O21	2.3224	0.325399		O21	2.296	0.349465
			**5.157102**				**5.150012**
O5	Mo2	2.1291	0.548663	O13	Mo3	2.1185	0.564609
	Mo1	2.1392	0.533889		Mo4	2.1187	0.564304
			**1.082552**				**1.128913**
O22	Mo5	2.1033	0.588287				
	Mo6	2.1312	0.545558				
			**1.133845**				

Table S10. Bond valence sum (BVS) values for **$NaNH_4$-$BrC_6H_4AsMo_6$**. Bond Valence (v_{ij}) = exp[(R_{ij}-d_{ij})/b (R_{ij} = bond valence parameter; b = 0.37); Bond Valence Sum (V_i) = $\Sigma_j v_{ij}$.

i	j	d_{ij} (Å)	v_{ij}	i	j	d_{ij} (Å)	v_{ij}
Mo1	O1	1.6727	1.88371	Mo2	O8	1.6912	1.79184
	O2	1.9375	0.920874		O9	1.9343	0.928873
	O3	1.9679	0.848238		O10	1.9459	0.900203
	O6	2.0614	0.658825		O7	2.0909	0.608337
	O5	2.1253	0.554327		O5	2.1252	0.554477
	O4	2.297	0.348522		O4	2.2919	0.353359
			**5.214495**				**5.137089**
Mo3	O11	1.679	1.851908	Mo4	O16	1.6885	1.804964
	O9	1.9424	0.908759		O18	1.9213	0.962089
	O10	1.9503	0.889561		O17	1.9454	0.90142
	O12	2.0877	0.613621		O15	2.1016	0.590996
	O13	2.1215	0.56005		O13	2.1104	0.577106
	O14	2.308	0.338313		O14	2.3096	0.336853
			**5.162211**				**5.173428**
Mo5	O19	1.6837	1.828532	Mo6	O24	1.6791	1.851407
	O18	1.9433	0.906551		O3	1.9379	0.919879
	O17	1.9479	0.89535		O2	1.9488	0.893175
	O20	2.0737	0.637284		O23	2.0823	0.622642
	O22	2.1236	0.55688		O22	2.1299	0.547478
	O21	2.2577	0.387578		O21	2.3021	0.343751
			**5.212175**				**5.178332**
O5	Mo2	2.1252	0.554477	O13	Mo4	2.1104	0.577106
	Mo1	2.1253	0.554327		Mo3	2.1215	0.56005
			**1.108804**				**1.137155**
O22	Mo5	2.1236	0.55688				
	Mo6	2.1299	0.547478				
			**1.104358**				

Table S11. Bond valence sum (BVS) values for **$NaNH_4$-$N_3C_6H_4AsMo_6$**. Bond Valence (v_{ij}) = exp[(R_{ij}-d_{ij})/b (R_{ij} = bond valence parameter; b = 0.37); Bond Valence Sum (V_i) = $\Sigma_j v_{ij}$.

i	j	d_{ij} (Å)	v_{ij}	i	j	d_{ij} (Å)	v_{ij}
Mo1	O1	1.6796	1.848907	Mo2	O8	1.6838	1.828038
	O2	1.9315	0.935928		O9	1.9406	0.91319
	O3	1.9778	0.825842		O10	1.9483	0.894383
	O6	2.0841	0.61962		O7	2.0768	0.631966
	O5	2.1254	0.554177		O5	2.096	0.600009
	O4	2.2929	0.352405		O4	2.3403	0.310032
			**5.136881**				**5.177618**
Mo3	O11	1.682	1.836953	Mo4	O16	1.6844	1.825076
	O9	1.9401	0.914425		O18	1.944	0.904837
	O10	1.9479	0.89535		O17	1.9527	0.88381
	O12	2.0969	0.598551		O15	2.0627	0.656514
	O13	2.1277	0.550743		O13	2.0976	0.59742
	O14	2.2912	0.354028		O14	2.291	0.35422
			**5.150051**				**5.221877**
Mo5	O19	1.6956	1.770658	Mo6	O24	1.691	1.792809
	O17	1.9383	0.918885		O3	1.9242	0.954577
	O18	1.9502	0.889802		O2	1.9452	0.901908
	O20	2.0644	0.653505		O23	2.1072	0.582119
	O22	2.1191	0.563694		O22	2.1232	0.557482

	O21	2.2877	0.357393		O21	2.2805	0.364416
			**5.153937**				**5.153311**
O5	Mo2	2.096	0.600009	O13	Mo4	2.0976	0.59742
	Mo1	2.1254	0.554177		Mo3	2.1277	0.550743
			**1.154186**				**1.148163**
O22	Mo5	2.1191	0.563694				
	Mo6	2.1232	0.557482				
			**1.121177**				

Table S12. Bond valence sum (BVS) values for **$NaNH_4$-$H_2O_4C_2C_6H_3AsMo_6$**. Bond Valence (v_{ij}) = exp[(R_{ij}-d_{ij})/b (R_{ij} = bond valence parameter; b = 0.37); Bond Valence Sum (V_i) = $\Sigma_j v_{ij}$.

i	j	d_{ij} (Å)	v_{ij}	i	j	d_{ij} (Å)	v_{ij}
Mo1	O1	1.69	1.797661	Mo2	O8	1.6887	1.803988
	O3	1.9471	0.897288		O9	1.9296	0.940747
	O2	1.9491	0.892451		O10	1.9384	0.918636
	O5	2.0607	0.660073		O7	2.0935	0.604077
	O6	2.1271	0.551637		O6	2.1221	0.559142
	O4	2.269	0.37592		O4	2.2938	0.351549
			**5.17503**				**5.17814**
Mo3	O11	1.6888	1.803501	Mo4	O16	1.6826	1.833976
	O9	1.9344	0.928621		O17	1.9417	0.91048
	O10	1.9559	0.876199		O18	1.9428	0.907777
	O12	2.0711	0.641778		O15	2.0923	0.606039
	O13	2.1309	0.546001		O13	2.1118	0.574926
	O14	2.2785	0.366391		O14	2.2825	0.362451
			**5.162491**				**5.195649**
Mo5	O19	1.6733	1.880658	Mo6	O24	1.6895	1.800092
	O17	1.9323	0.933907		O3	1.9408	0.912697
	O18	1.9386	0.91814		O2	1.9464	0.898987
	O20	2.0776	0.630602		O23	2.0811	0.624665
	O22	2.1267	0.552234		O22	2.1094	0.578668
	O21	2.2984	0.347206		O21	2.2943	0.351075
			**5.262746**				**5.166183**
O6	Mo2	2.1221	0.559142	O13	Mo4	2.1118	0.574926
	Mo1	2.1271	0.551637		Mo3	2.1309	0.546001
			**1.110779**				**1.120927**
O22	Mo6	2.1094	0.578668	O26	C7	1.2779	**1.353878**
	Mo5	2.1267	0.552234	O27	C8	1.262	**1.413326**
			**1.130901**				

Table S13. ESI mass spectrometry data of polyoxometalates investigated in negative ion mode. *m/z* values always refer to the most intense peak of isotope envelope cluster. The molecular formulae stated refers to the best fit for a simulated mass spectrum.

Compound	Monomeric 2- ion *m/z* and elemental composition	Monomeric – ion *m/z*	Dimeric 2- ion *m/z* and elemental composition	Ratio Monomer/Dimer
**$HOAsMo_6$**	695.55 $Mo_6As_4C_6H_{22}O_{25}$ $Mo_6As_4C_6H_{21}O_{25}Na$	1391.09 $Mo_6As_4C_6H_{22}O_{25}Na$	1391.61 $Mo_{12}As_8C_{12}H_{44}O_{50}Na_2$	9.1
**CH_3AsMo_6**	683.56 $Mo_6As_4C_7H_{24}O_{24}$	1390.12 $Mo_6As_4C_7H_{24}O_{24}Na$	1390.62 $Mo_{12}As_8C_{14}H_{48}O_{48}Na_2$	8.3
**$C_2H_5AsMo_6$**	690.54 $Mo_6As_4C_8H_{26}O_{24}$	1404.07 $Mo_6As_4C_8H_{26}O_{24}Na$	1404.57 $Mo_{12}As_8C_{16}H_{52}O_{48}Na_2$	8.3
**$C_6H_5AsMo_6$**	714.55 $Mo_6As_4C_{12}H_{26}O_{24}$	1452.09 $Mo_6As_4C_{12}H_{26}O_{24}Na$	1452.69 $Mo_{12}As_8C_{24}H_{52}O_{48}Na_2$	6.5
**4-$FC_6H_4AsMo_6$**	723.54 $Mo_6As_4FC_{12}H_{25}O_{24}$	1469.08 $Mo_6As_4FC_{12}H_{25}O_{24}Na$	1469.61 $Mo_{12}As_8F_2C_{24}H_{50}O_{48}Na_2$	3.1
**4-$BrC_6H_4AsMo_6$**	754.56 $Mo_6As_4BrC_{12}H_{25}O_{24}$	1531.09 $Mo_6As_4BrC_{12}H_{25}O_{24}Na$	1531.58 $Mo_{12}As_8Br_2C_{24}H_{50}O_{48}Na_2$	3.4
**4-$CF_3C_6H_4AsMo_6$**	748.60 $Mo_6As_4F_3C_{13}H_{25}O_{24}$	1520.20 $Mo_6As_4F_3C_{13}H_{25}O_{24}Na$	1520.50 $Mo_{12}As_8F_6C_{26}H_{50}O_{48}Na_2$	2.6
**4-$F_3COC_6H_4AsMo_6$**	750.54 $Mo_6As_4F_3C_{13}H_{25}O_{25}$	1534.16 $Mo_6As_4F_3C_{13}H_{25}O_{25}Na$	1533.68 $Mo_{12}As_8F_6C_{26}H_{50}O_{50}Na_2$	3.4
**4-$N_3C_6H_4AsMo_6$**	729.05 $Mo_6As_4N_3C_{12}H_{25}O_{24}$	1493.18 $Mo_6As_4N_3C_{12}H_{25}O_{24}Na$	1493.67 $Mo_{12}As_8N_6C_{24}H_{50}O_{48}Na_2$	4.1

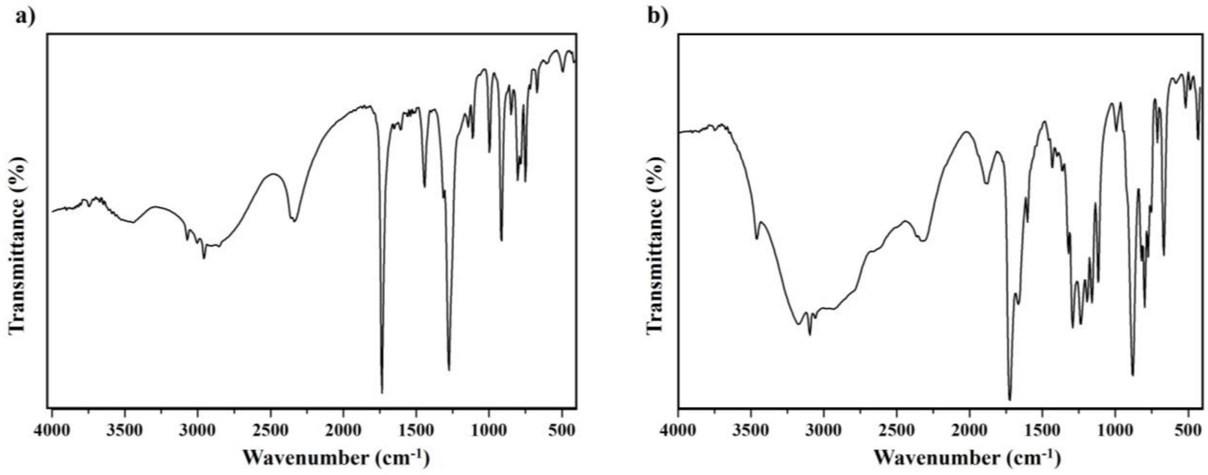

Figure S1. FT-IR spectra of (a) 3,5-bis(methoxycarbonyl)phenylarsonic acid and (b) 3,5-bis(carboxy)phenylarsonic acid on KBr pellet.

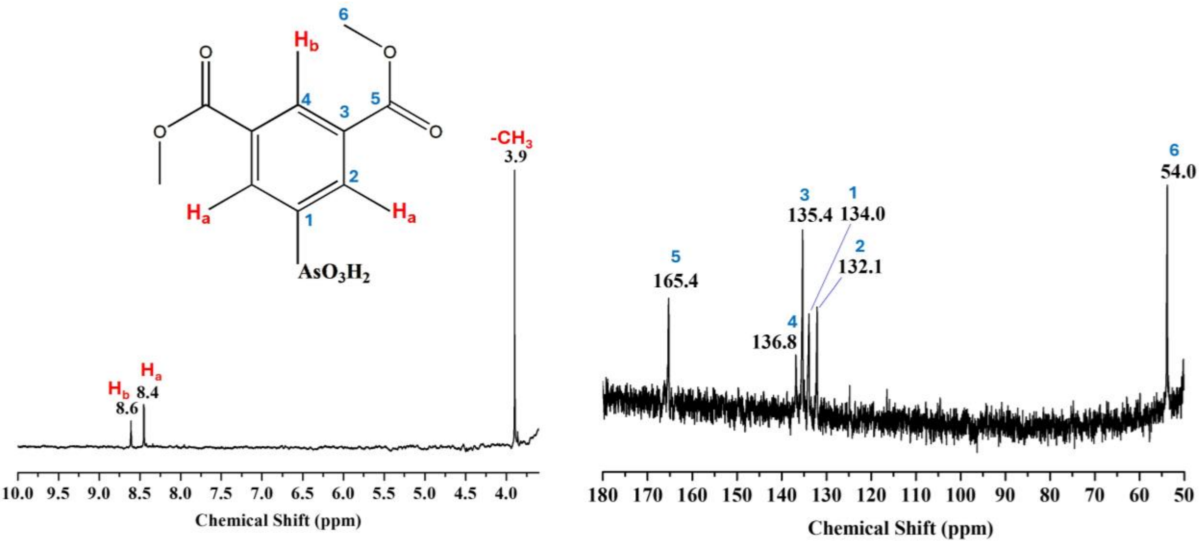

Figure S2. ^{1}H (left) and $^{13}C\{^{1}H\}$ (right) NMR spectra 3,5-bis(methoxycarbonyl)phenylarsonic acid, recorded at room temperature in DMSO-d_6.

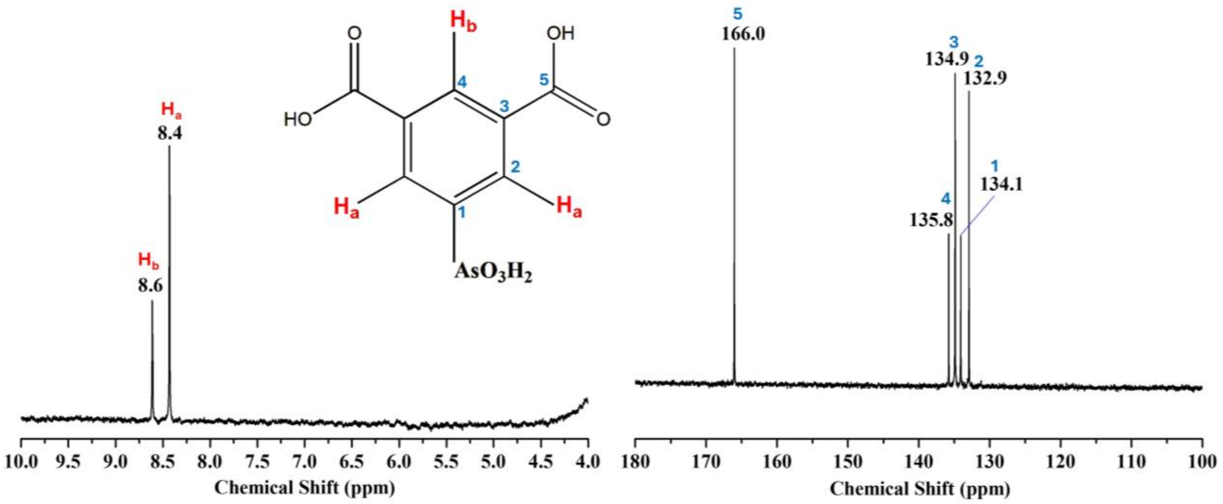

Figure S3. ^{1}H (left) and $^{13}C\{^{1}H\}$ (right) NMR spectra 3,5-bis(carboxy)phenylarsonic acid, recorded at room temperature in DMSO-d_6.

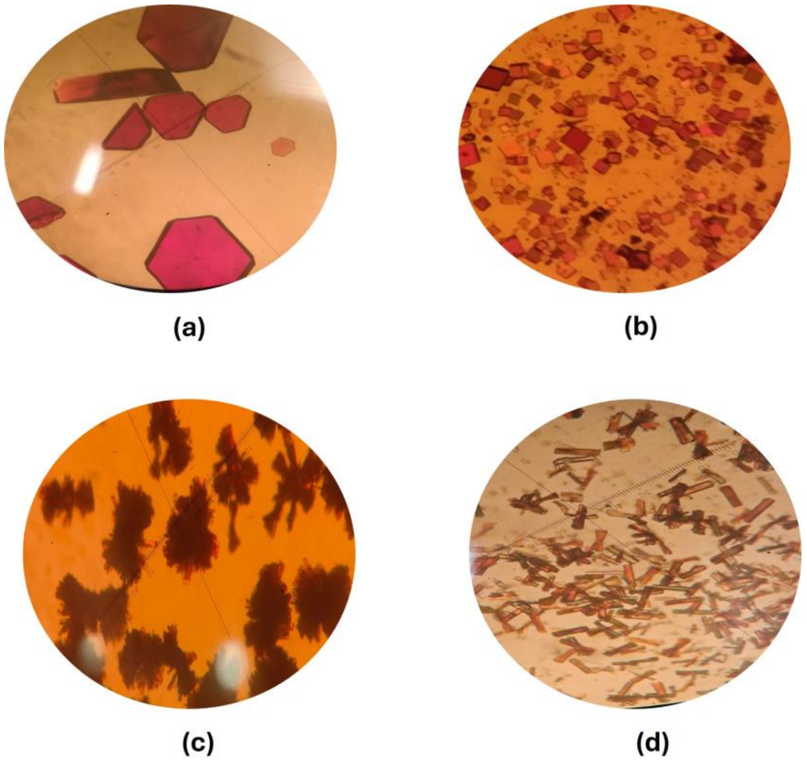

Figure S4. Optical micrographs of crystals of (a) **NaNH$_4$-As^{III}Mo$_6$** (b) **NaNH$_4$-As^{V}Mo$_6$** (c) **NaNH$_4$-C$_2$H$_5$AsMo$_6$** (d) **NaNH$_4$-F$_3$COC$_6$H$_4$AsMo$_6$**

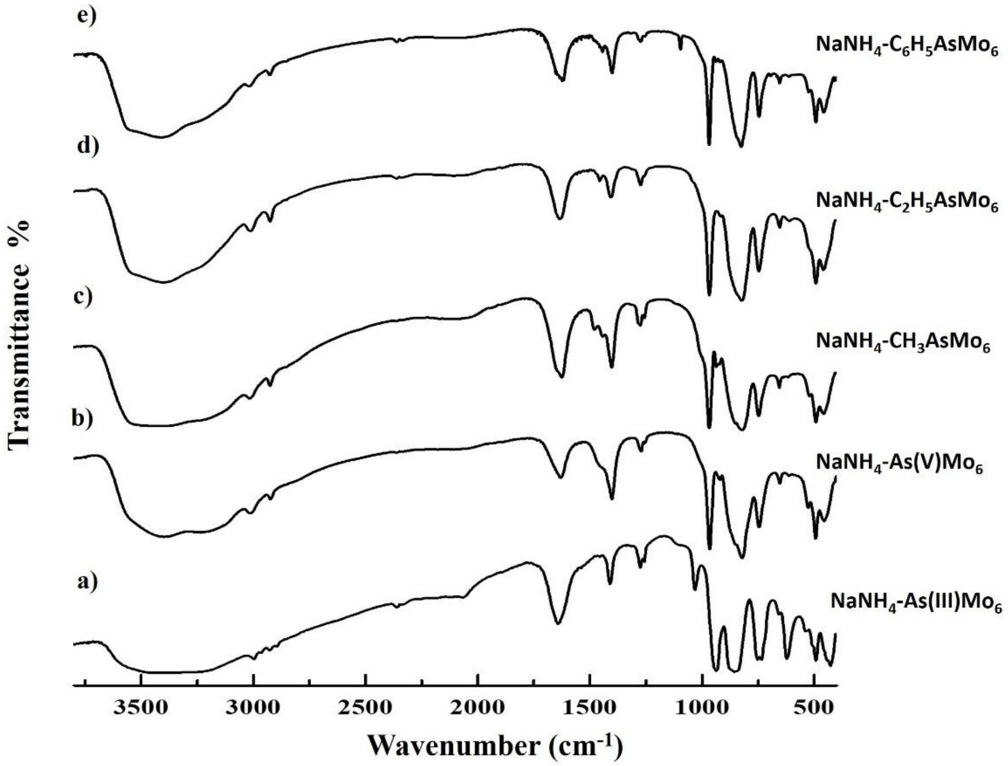

Figure S5. FT-IR spectra of (a) **$NaNH_4$-$As^{III}Mo_6$** (b) **$NaNH_4$-$As^{V}Mo_6$** (c) **$NaNH_4$-CH_3AsMo_6** (d) **$NaNH_4$-$C_2H_5AsMo_6$** and (e) **$NaNH_4$-$C_6H_5AsMo_6$** on KBr pellet.

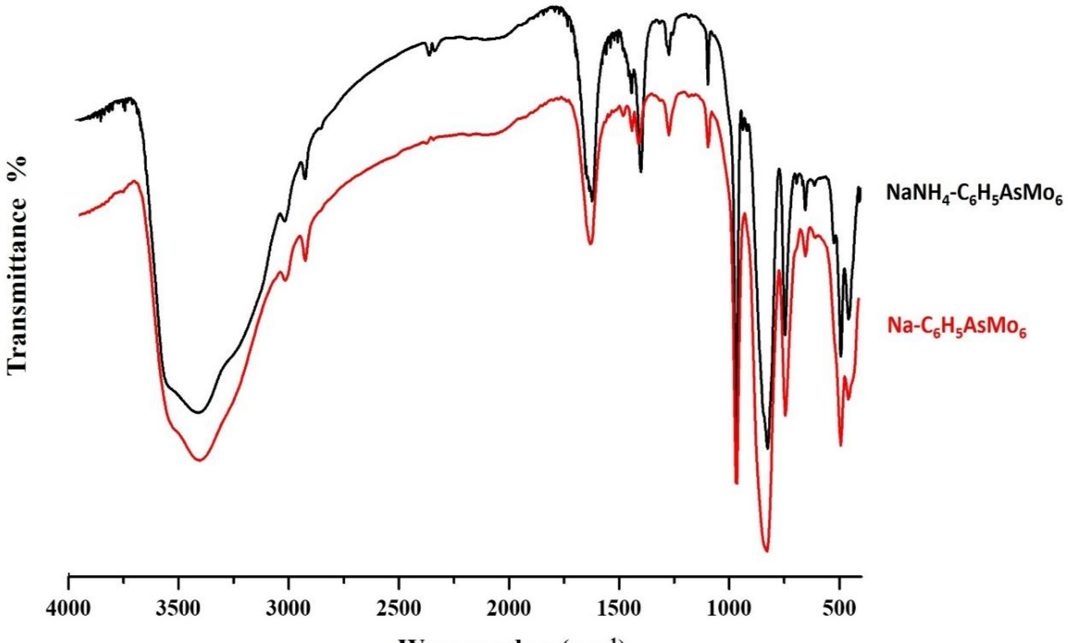

Figure S6. FT-IR spectra of **$NaNH_4$-$C_6H_5AsMo_6$** (black) and **Na-$C_6H_5AsMo_6$** (red) on a KBr pellet.

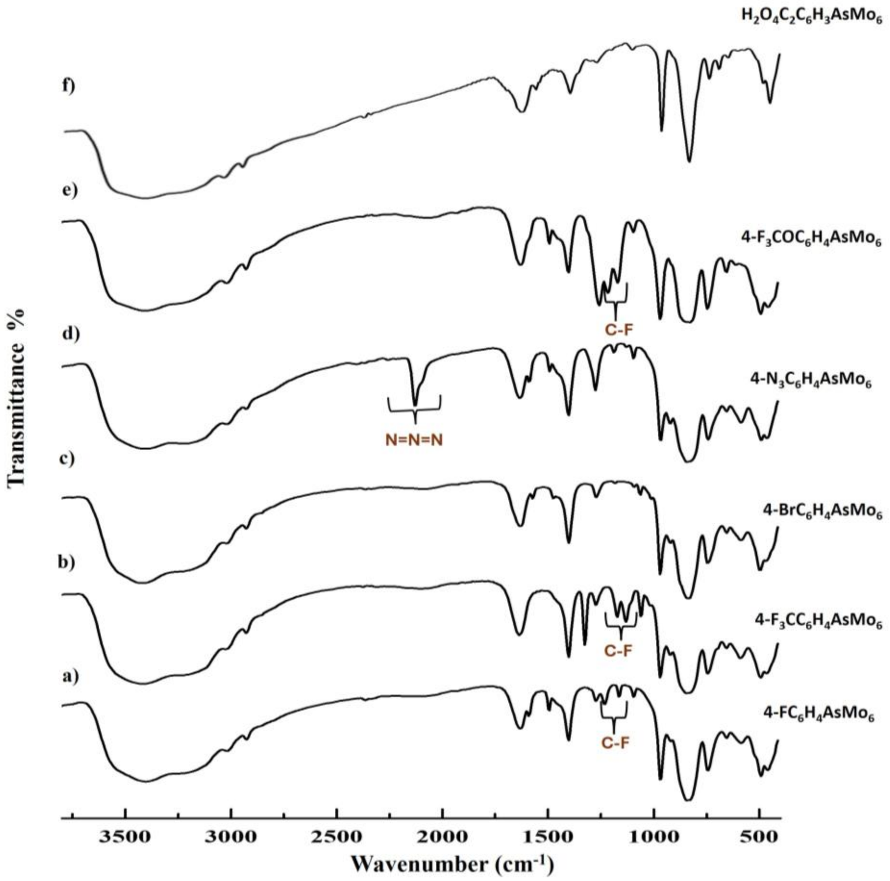

Figure S7. FT-IR spectra of (a) **$NaNH_4$-$FC_6H_4AsMo_6$** (b) **$NaNH_4$-$F_3CC_6H_4AsMo_6$** (c) **$NaNH_4$-$BrC_6H_4AsMo_6$** (d) **$NaNH_4$-$N_3C_6H_4AsMo_6$** (e) **$NaNH_4$-$F_3COC_6H_4AsMo_6$** and (f) **$NaNH_4$-$H_2O_4C_2C_6H_3AsMo_6$** on KBr pellet.

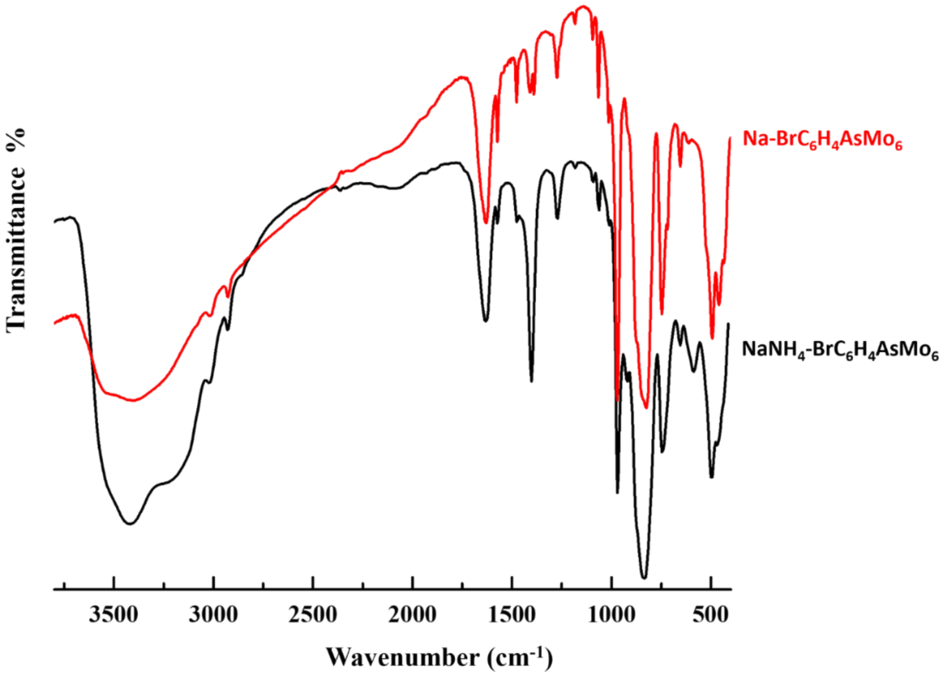

Figure S8. FT-IR spectra of **$NaNH_4$-$BrC_6H_4AsMo_6$** (black) and **Na-$BrC_6H_4AsMo_6$** (red) on a KBr pellet.

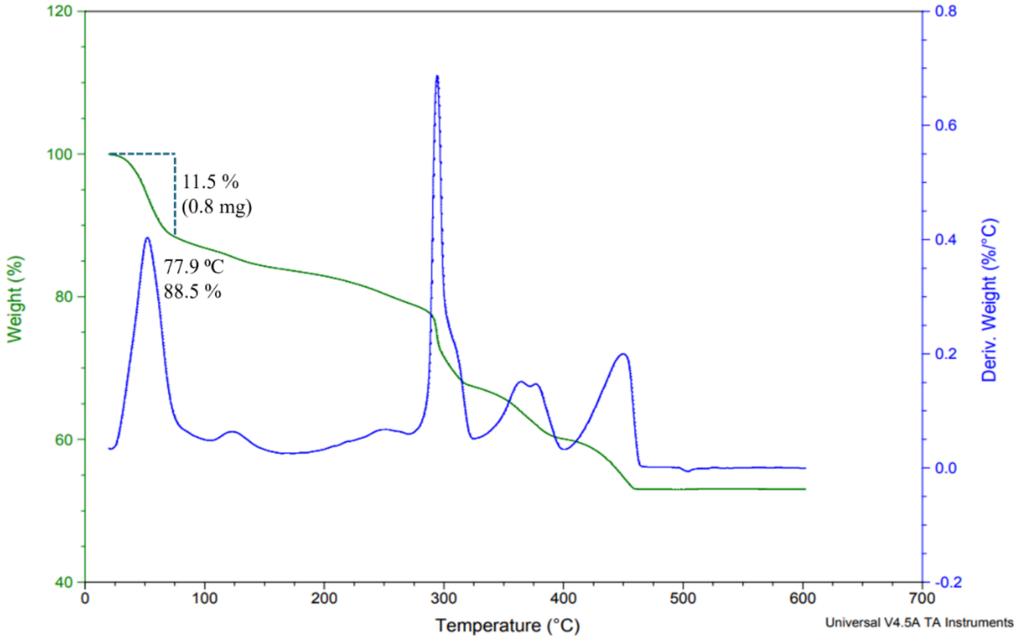

Figure S9. Thermogram of **NaNH4-As^{III}Mo6** from room temperature to 600 °C under N_2 atmosphere.

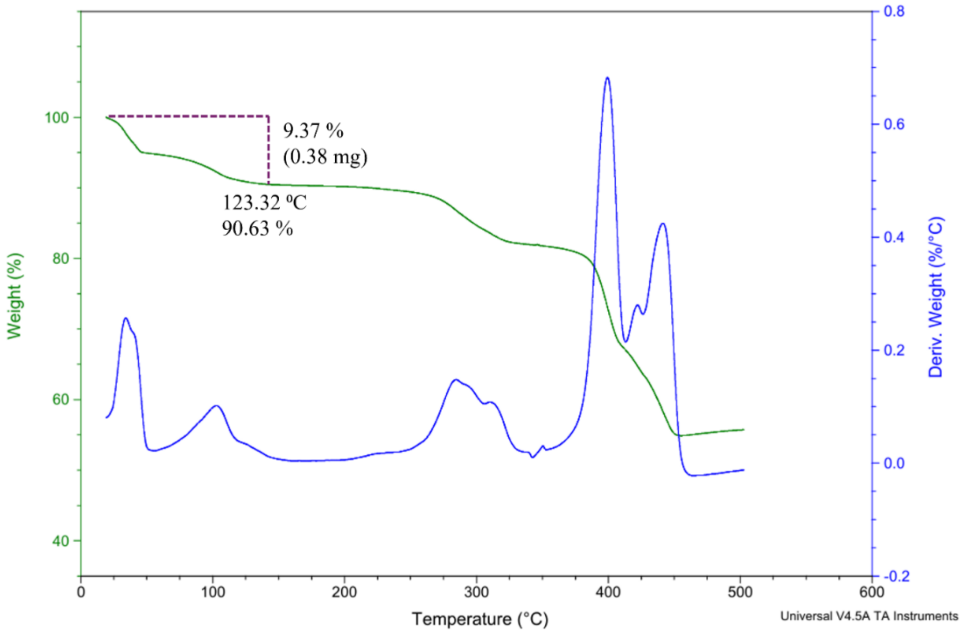

Figure S10. Thermogram of **NaNH4-As^{V}Mo6** from room temperature to 500 °C under N_2 atmosphere.

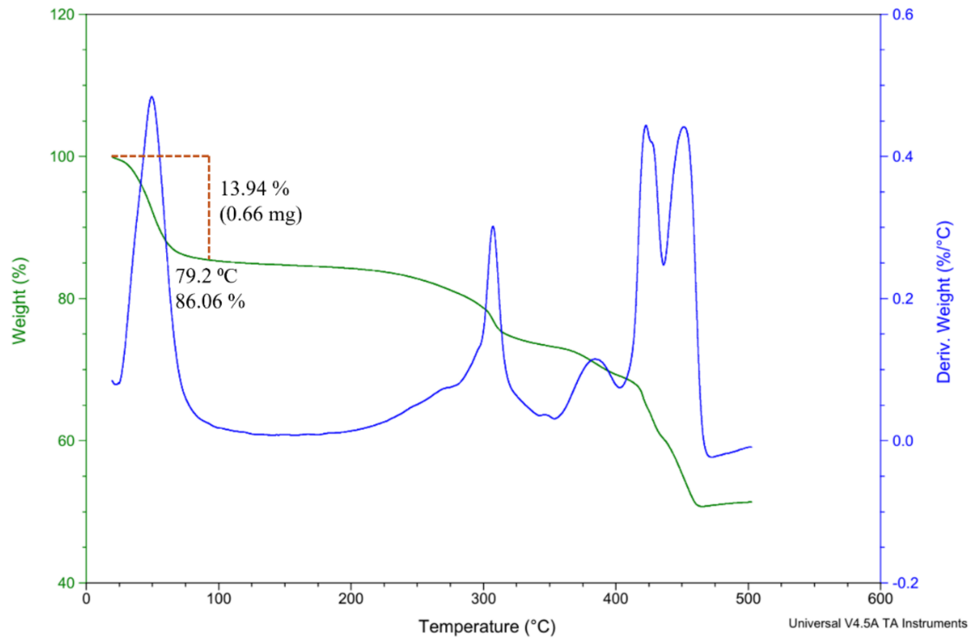

Figure S11. Thermogram of **$NaNH_4$-CH_3AsMo_6** from room temperature to 500 °C under N_2 atmosphere.

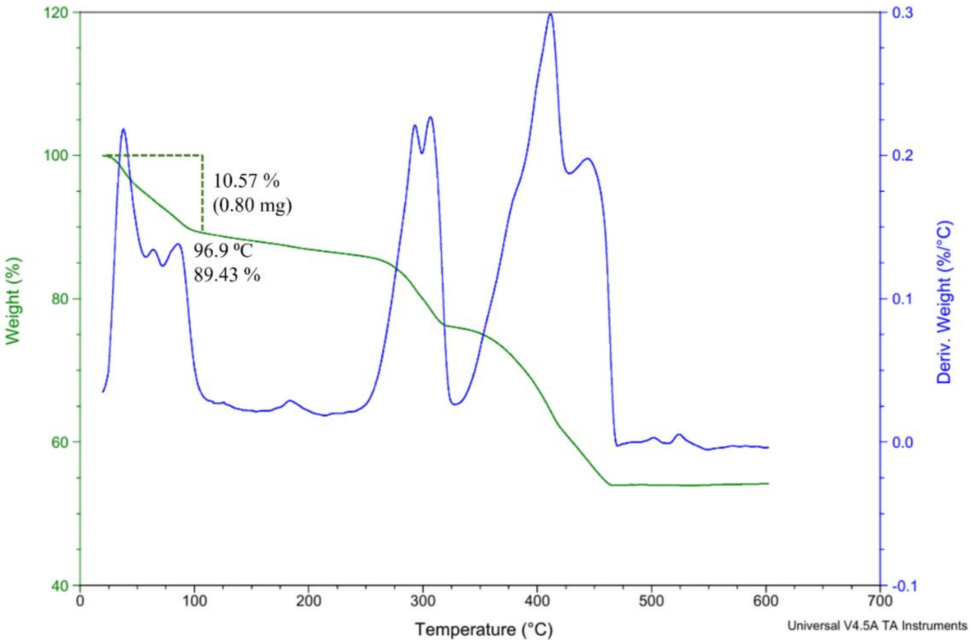

Figure S12. Thermogram of **$NaNH_4$-$C_2H_5AsMo_6$** from room temperature to 600 °C under N_2 atmosphere.

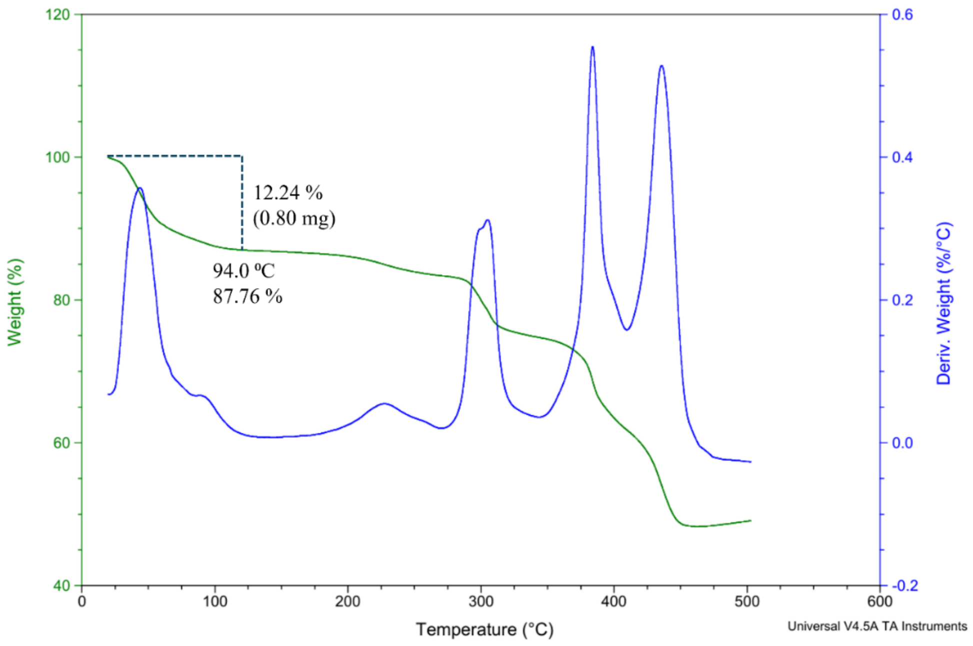

Figure S13. Thermogram of **NaNH4-C6H5AsMo6** from room temperature to 500 °C under N_2 atmosphere.

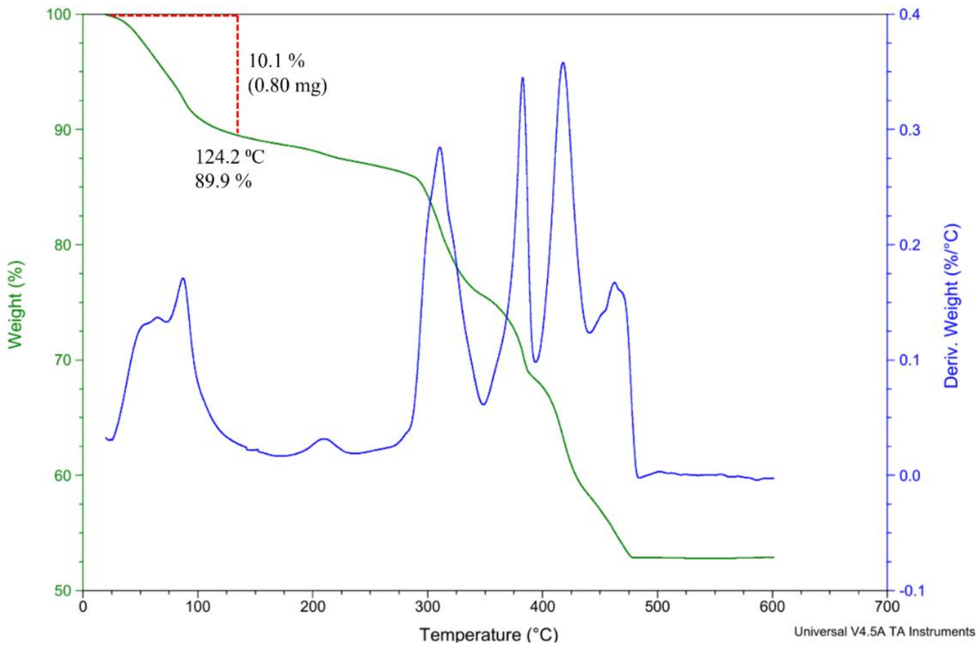

Figure S14. Thermogram of **NaNH4-FC6H4AsMo6** from room temperature to 600 °C under N_2 atmosphere.

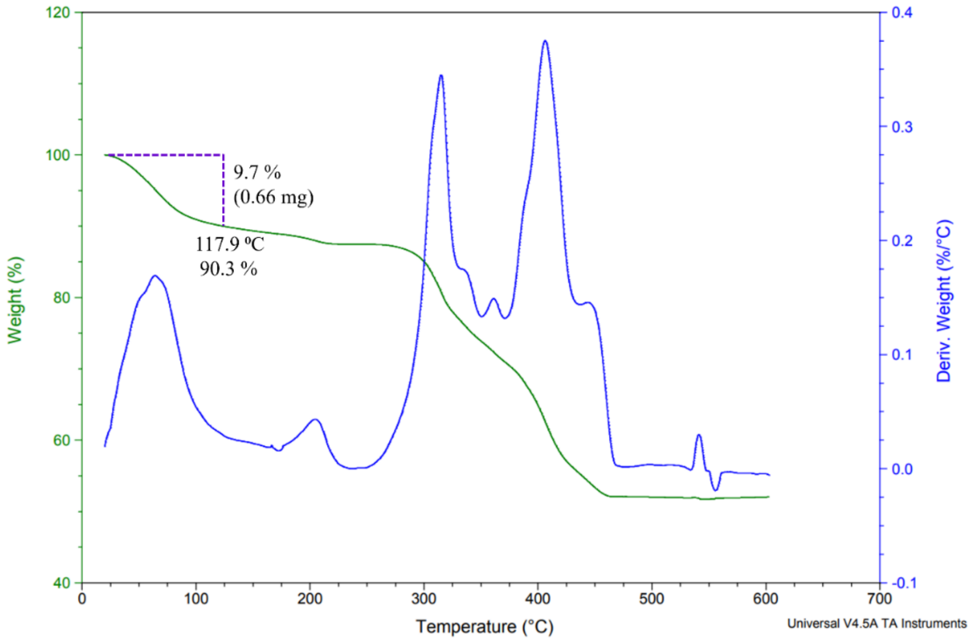

Figure S15. Thermogram of **NaNH4-F3CC6H4AsMo6** from room temperature to 600 °C under N_2 atmosphere.

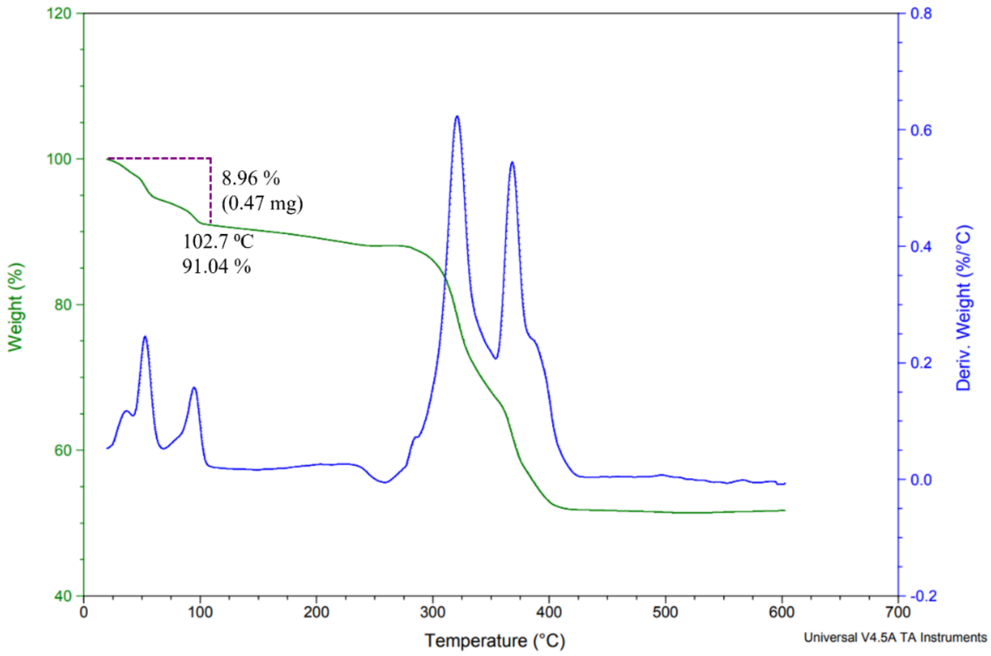

Figure S16. Thermogram of **NaNH4-F3COC6H4AsMo6** from room temperature to 600 °C under N_2 atmosphere.

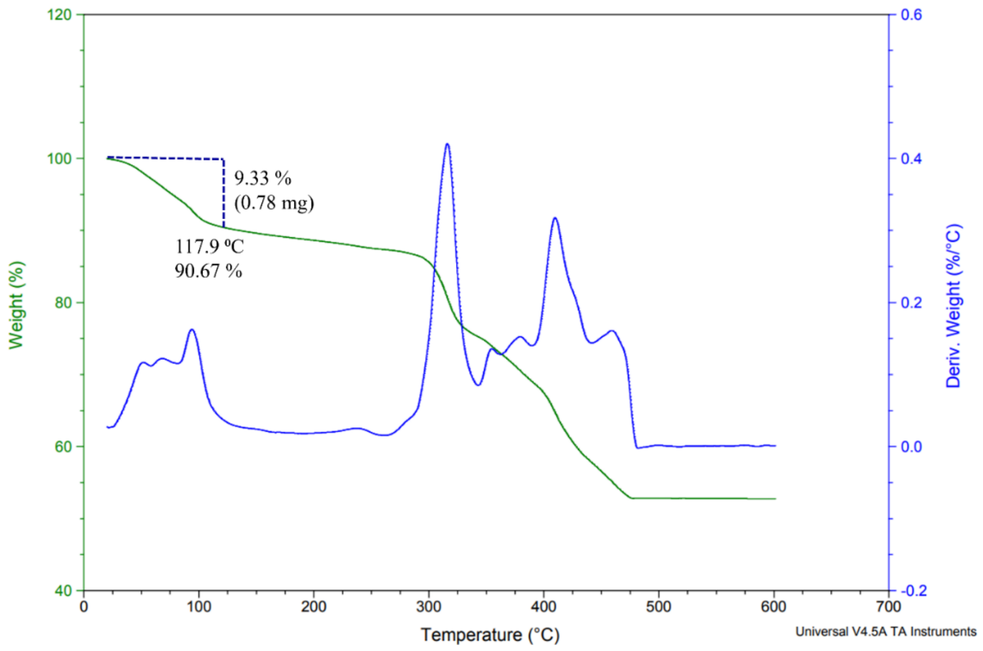

Figure S17. Thermogram of **$NaNH_4$-$BrC_6H_4AsMo_6$** from room temperature to 600 °C under N_2 atmosphere.

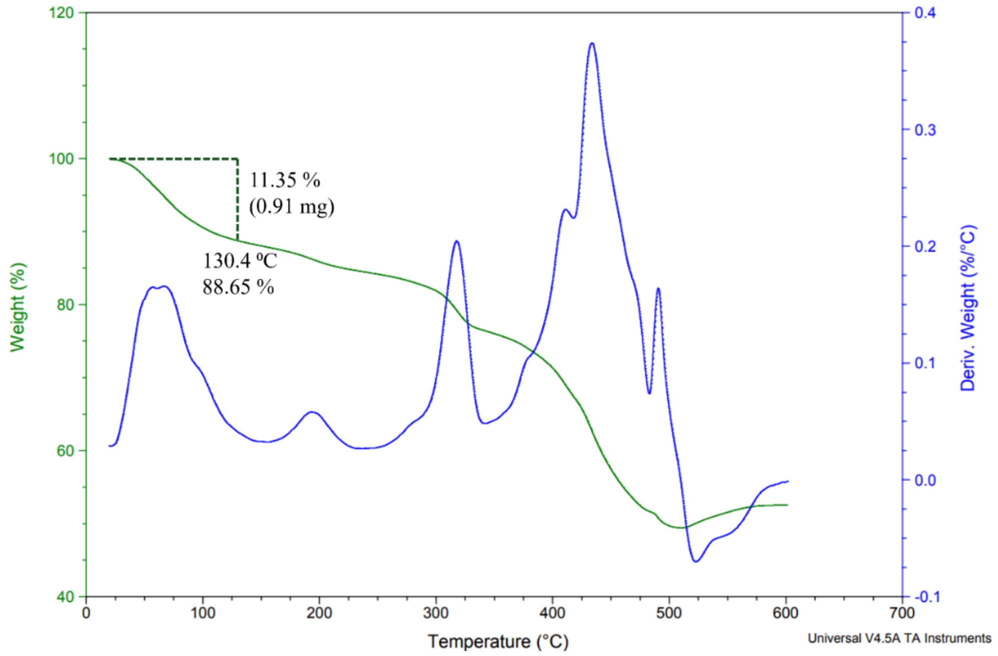

Figure S18. Thermogram of **$NaNH_4$-$N_3C_6H_4AsMo_6$** from room temperature to 600 °C under N_2 atmosphere.

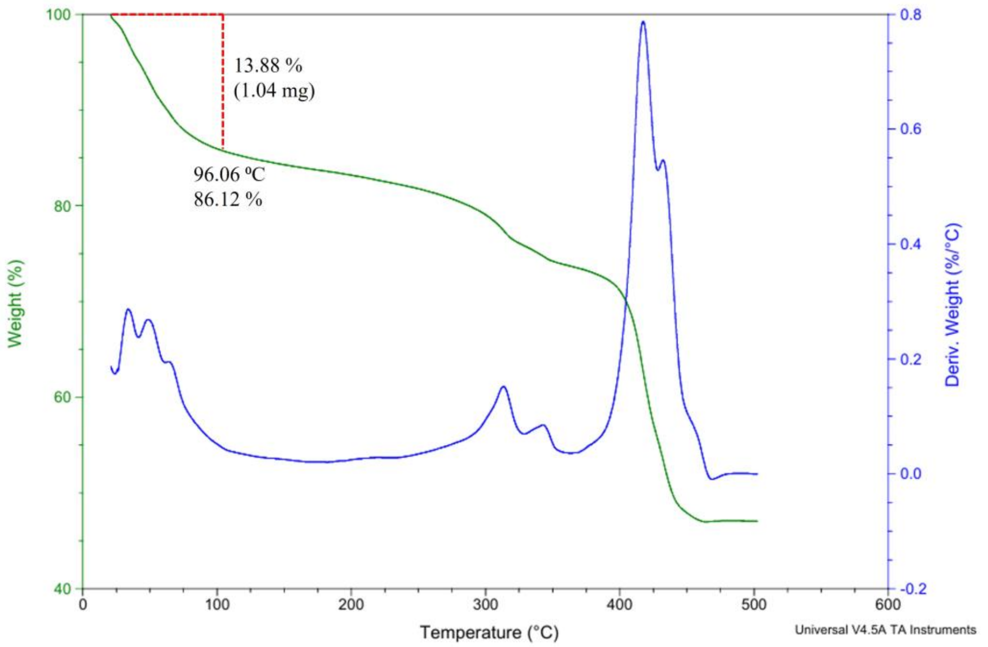

Figure S19. Thermogram of **NaNH4-H2O4C2C6H3AsMo6** from room temperature to 500 °C under N_2 atmosphere.

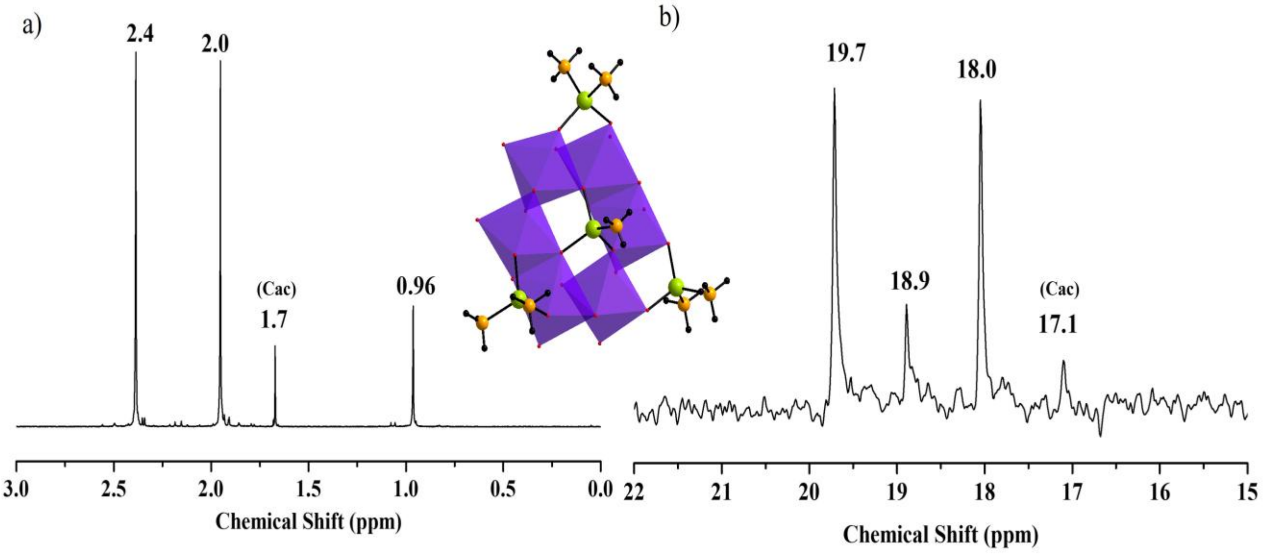

Figure S20. (a) ^{1}H and (b) $^{13}C\{^{1}H\}$ NMR spectra of **NaNH4-CH3AsMo6** dissolved in H_2O/D_2O at 40 °C.

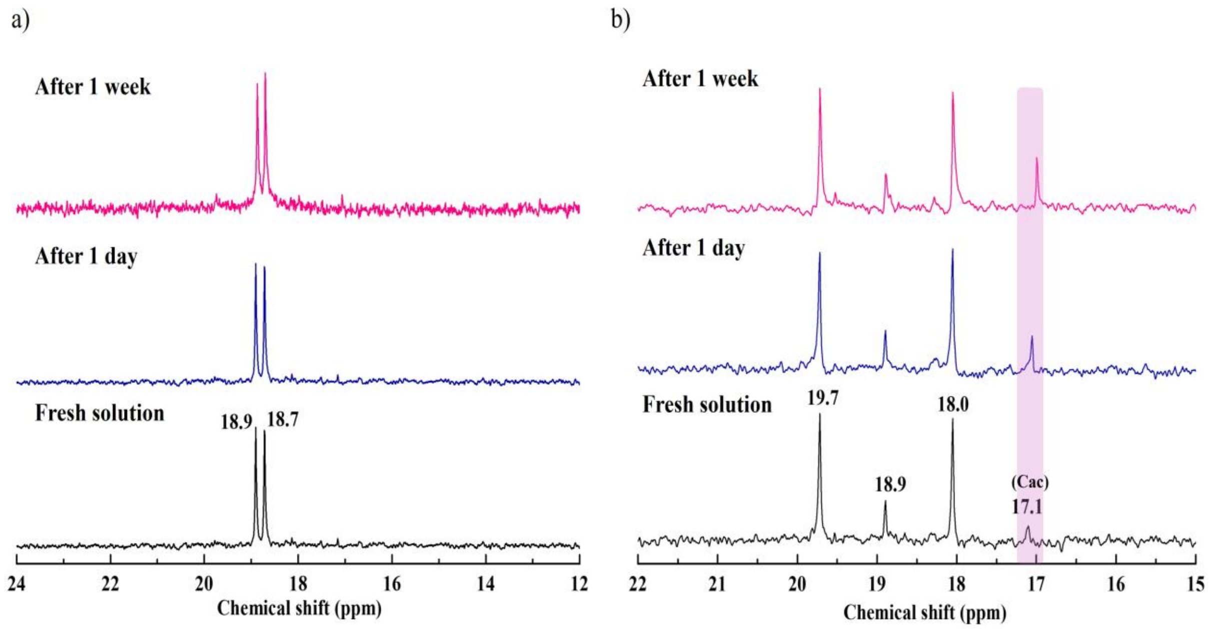

Figure S21. Time-dependent $^{13}C\{^{1}H\}$ spectra of (a) **NaNH$_4$-As^{V}Mo$_6$** and (b) **NaNH$_4$-CH$_3$AsMo$_6$** dissolved in H_2O/D_2O.

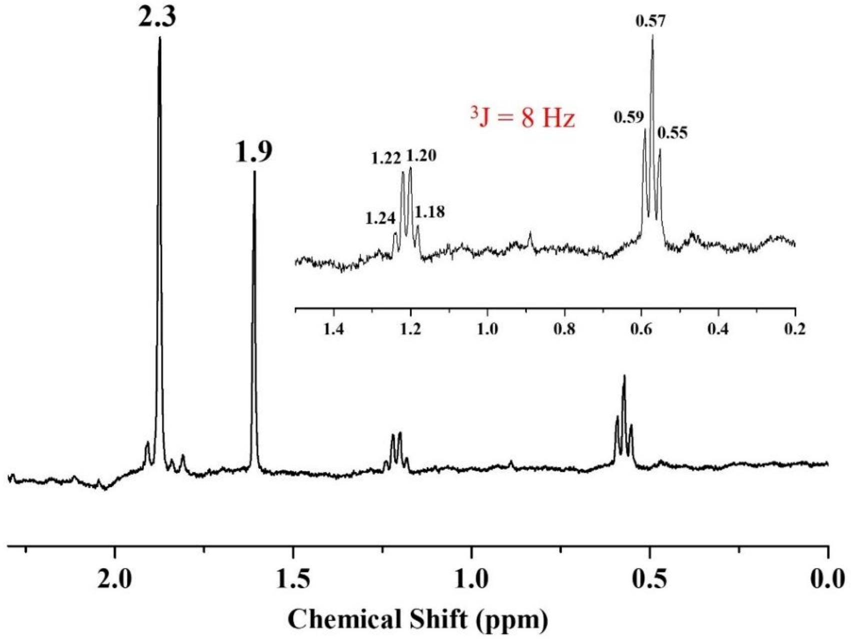

Figure S22. ^1H NMR spectra of **NaNH$_4$-C$_2$H$_5$AsMo$_6$** dissolved in H_2O/D_2O at 40 °C.

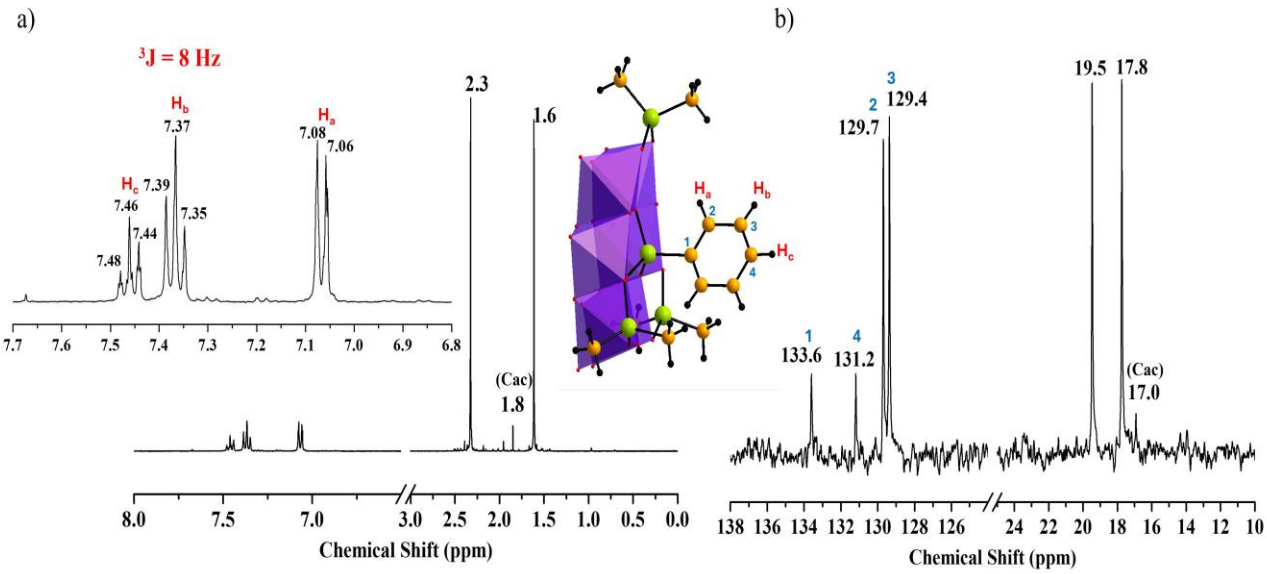

Figure S23. (a) ^{1}H and (b) $^{13}C\{^{1}H\}$ NMR spectra of **NaNH4-C6H5AsMo6** dissolved in H_2O/D_2O at room temperature.

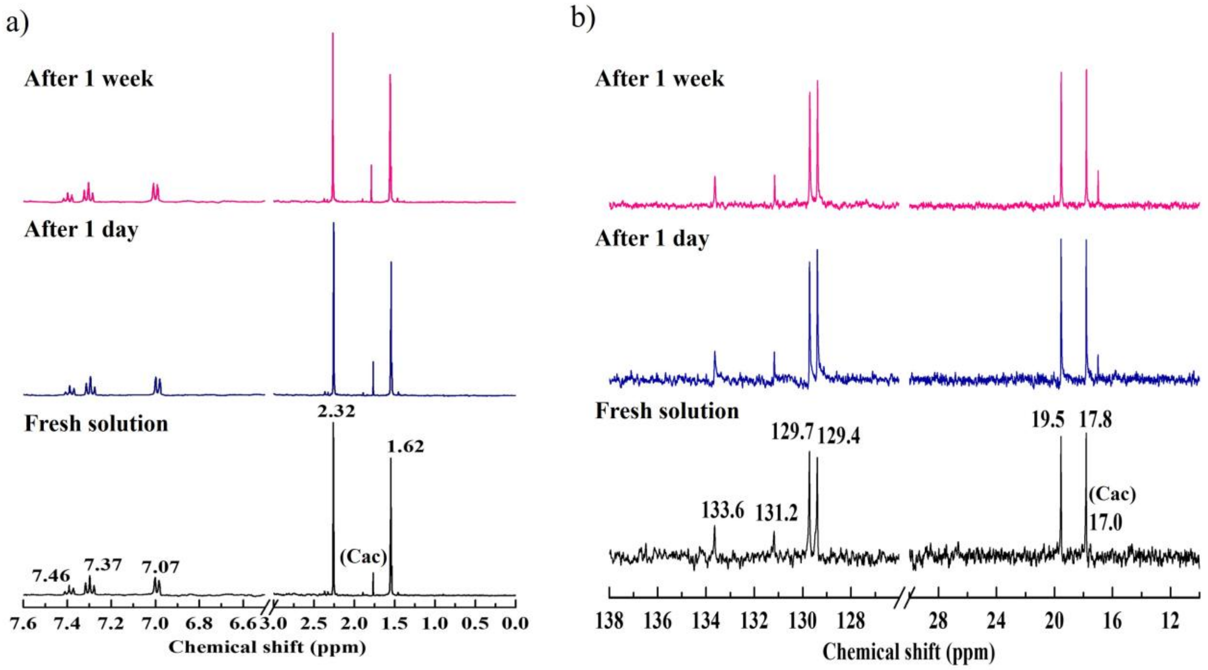

Figure S24. Time-dependent (a) ^{1}H and (b) $^{13}C\{^{1}H\}$ NMR spectra of **NaNH4-C6H5AsMo6** dissolved in H_2O/D_2O at room temperature.

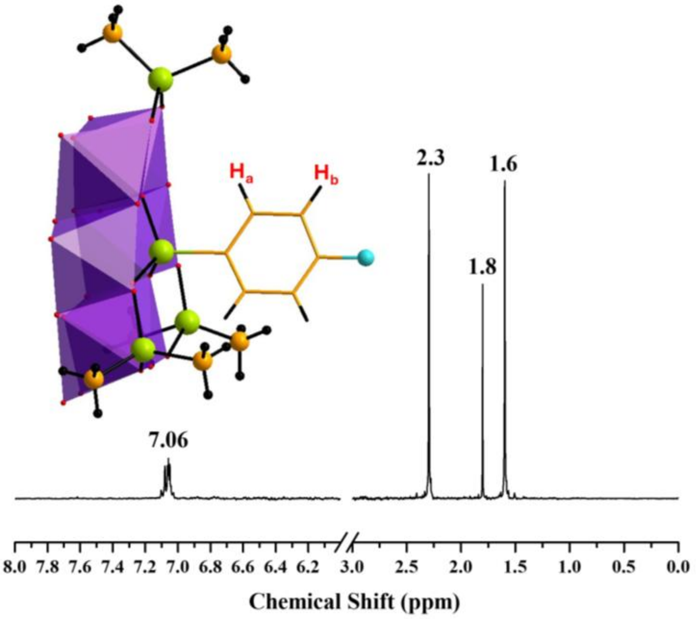

Figure S25. ^{1}H NMR spectrum of **NaNH4-FC6H4AsMo6** dissolved in H_2O/D_2O at room temperature.

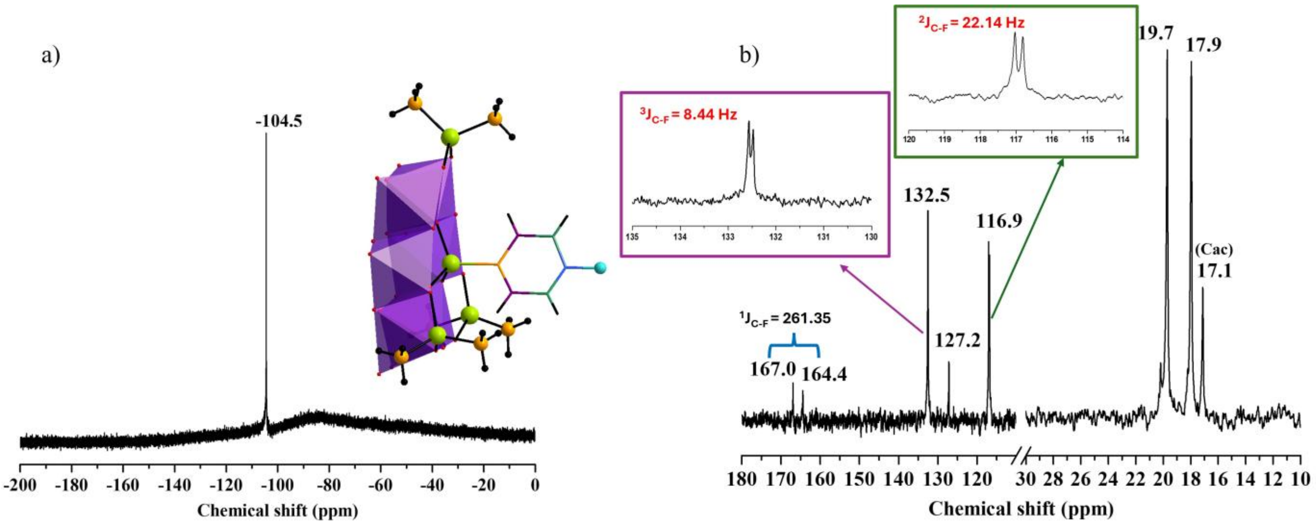

Figure S26. (a) ^{19}F and (b) ^{13}C{^{1}H} NMR spectra of **NaNH4-FC6H4AsMo6** dissolved in H_2O/D_2O at room temperature.

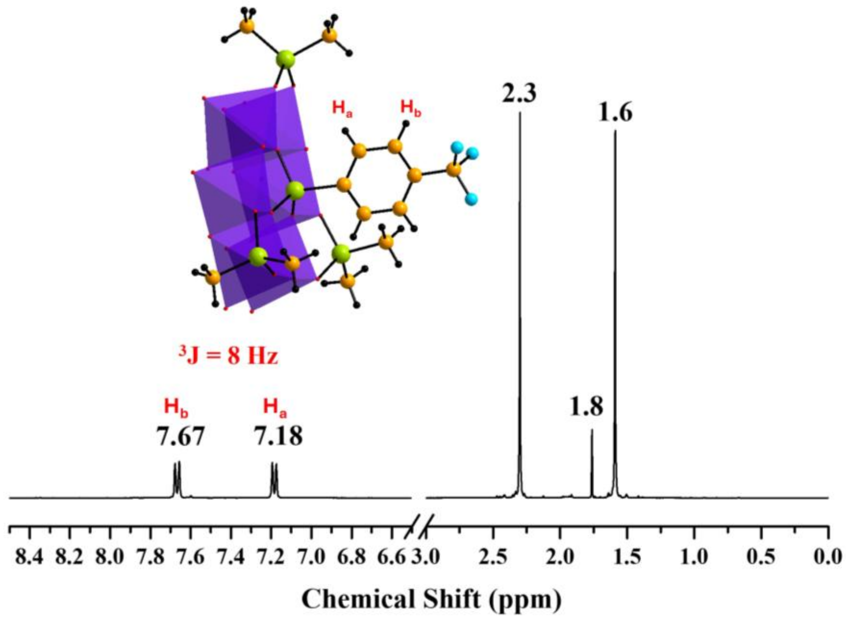

Figure S27. ¹H NMR spectrum of **NaNH$_4$-F$_3$CC$_6$H$_4$AsMo$_6$** dissolved in H_2O/D_2O at room temperature.

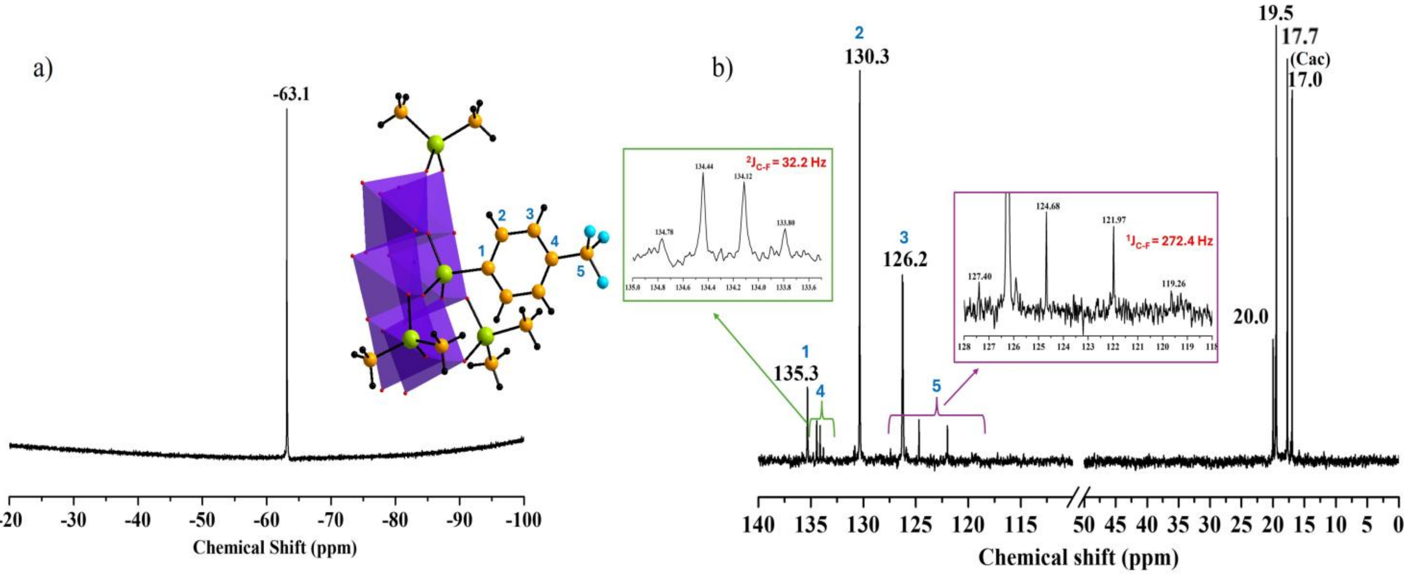

Figure S28. (a) ^{19}F and (b) ^{13}C{^{1}H} NMR spectra of **NaNH$_4$-F$_3$CC$_6$H$_4$AsMo$_6$** dissolved in H_2O/D_2O at room temperature.

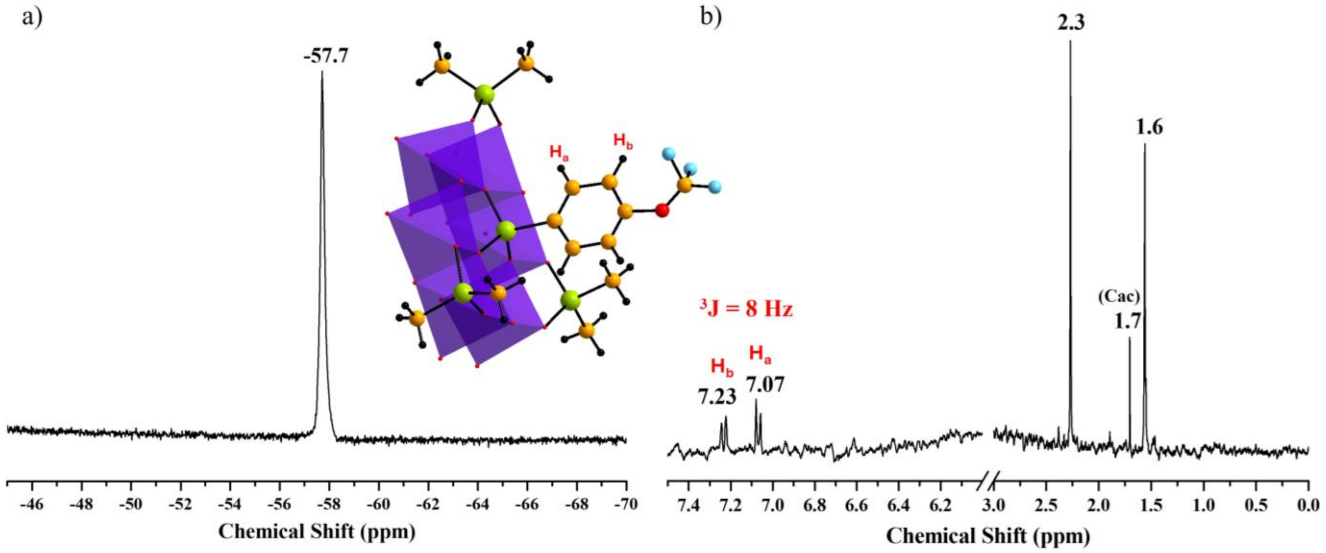

Figure S29. (a) ^{19}F and (b) ^{1}H NMR spectra of **NaNH$_4$-F$_3$COC$_6$H$_4$AsMo$_6$** dissolved in H_2O/D_2O at room temperature.

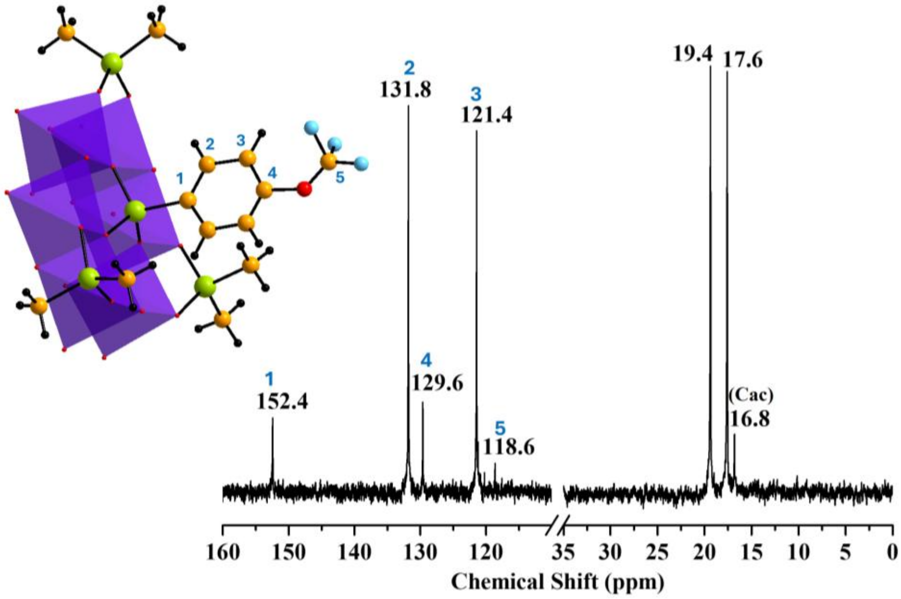

Figure S30. ^{13}C{^{1}H} NMR spectra of **NaNH$_4$-F$_3$COC$_6$H$_4$AsMo$_6$** dissolved in H_2O/D_2O at room temperature.

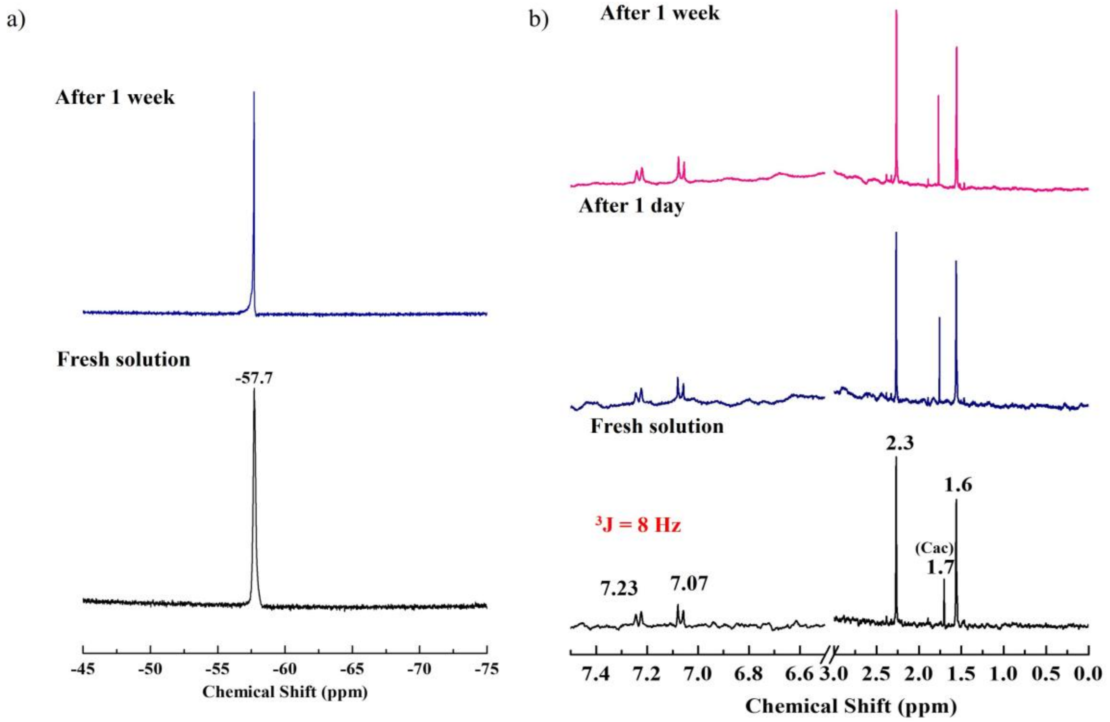

Figure S31. Time-dependent (a) ^{19}F and (b) ^{1}H NMR spectra of **NaNH$_4$-F$_3$COC$_6$H$_4$AsMo$_6$** dissolved in H_2O/D_2O at room temperature.

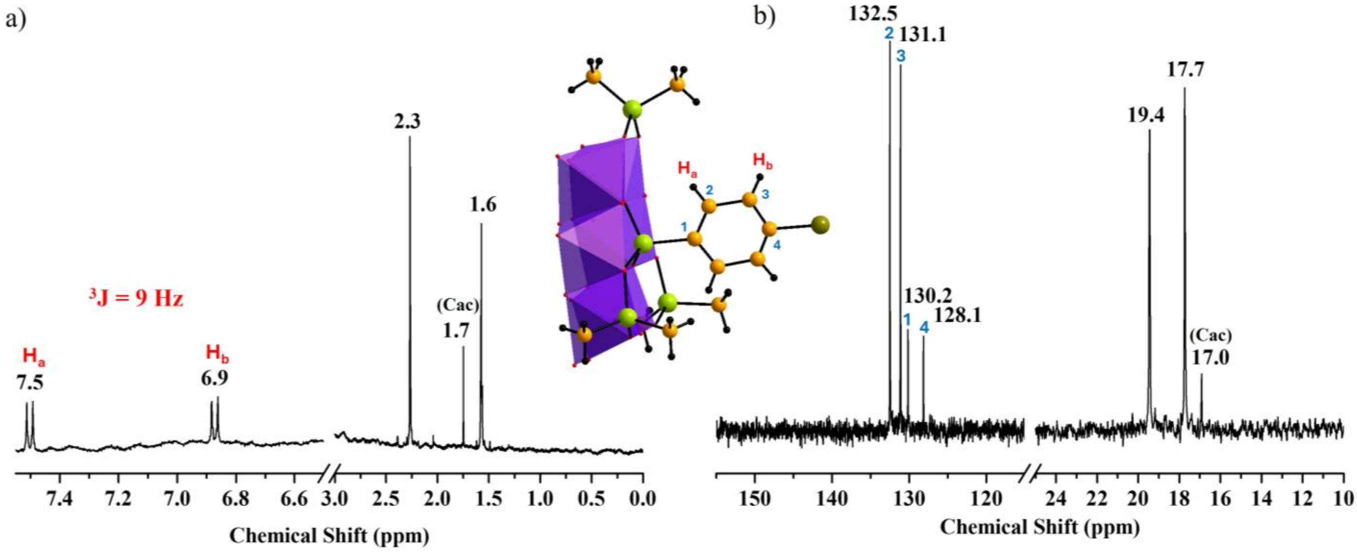

Figure S32. (a) ^{1}H and (b) ^{13}C{H}NMR spectra of **NaNH$_4$-BrC$_6$H$_4$AsMo$_6$** dissolved in H_2O/D_2O at room temperature.

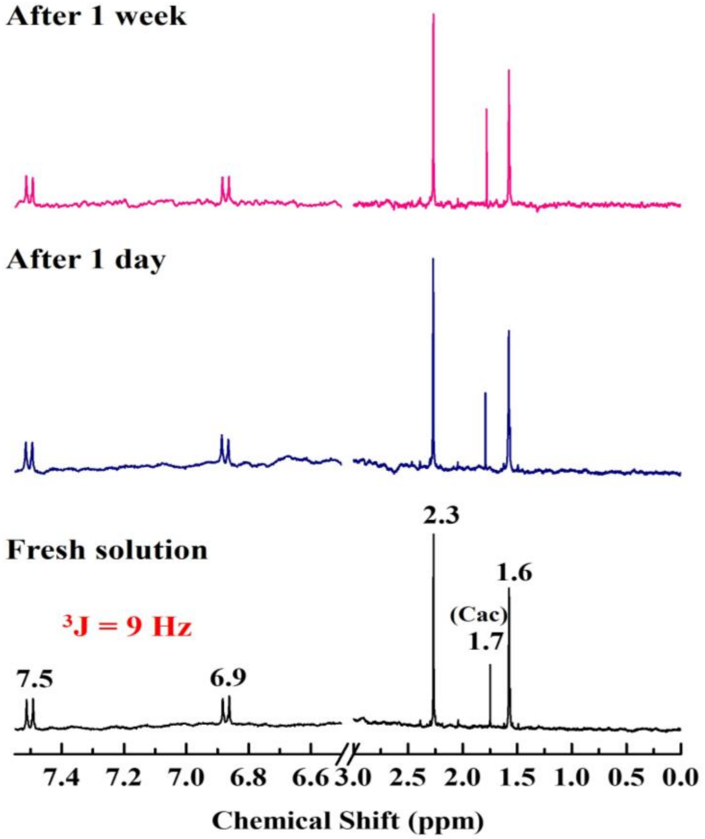

Figure S33. Time-dependent ^{1}H NMR spectra of **NaNH4-BrC6H4AsMo6** dissolved in H_2O/D_2O at room temperature.

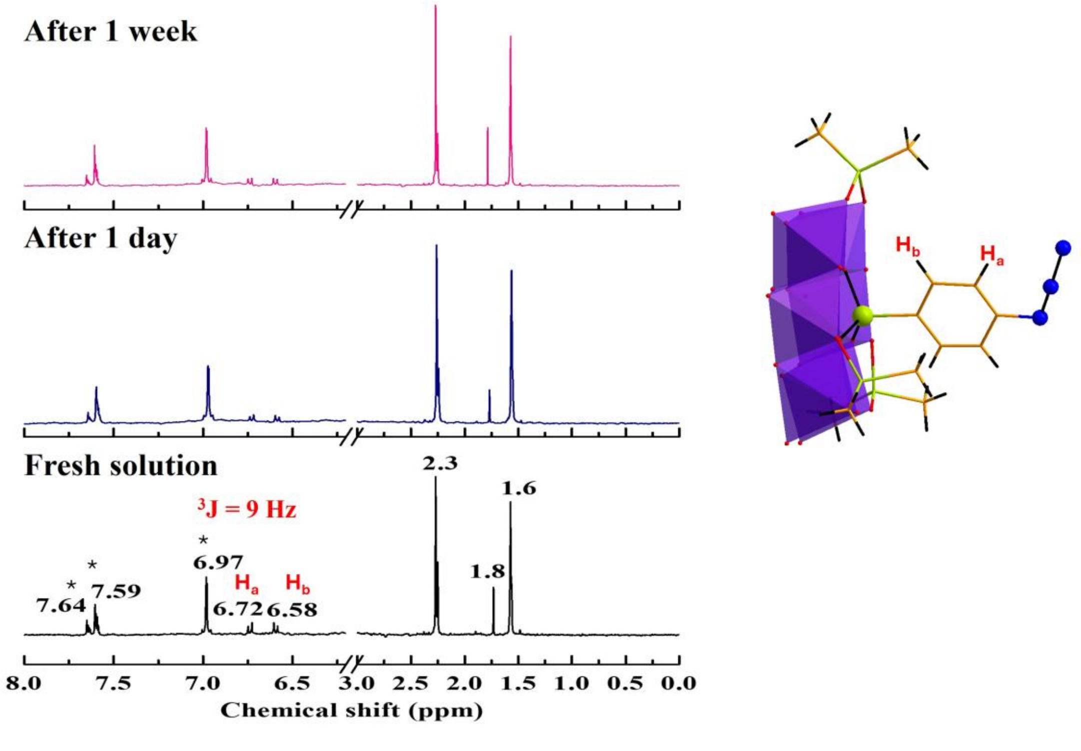

Figure S34. Time-dependent ^{1}H NMR spectra of **NaNH4-N3C6H4AsMo6** dissolved in H_2O/D_2O at room temperature.

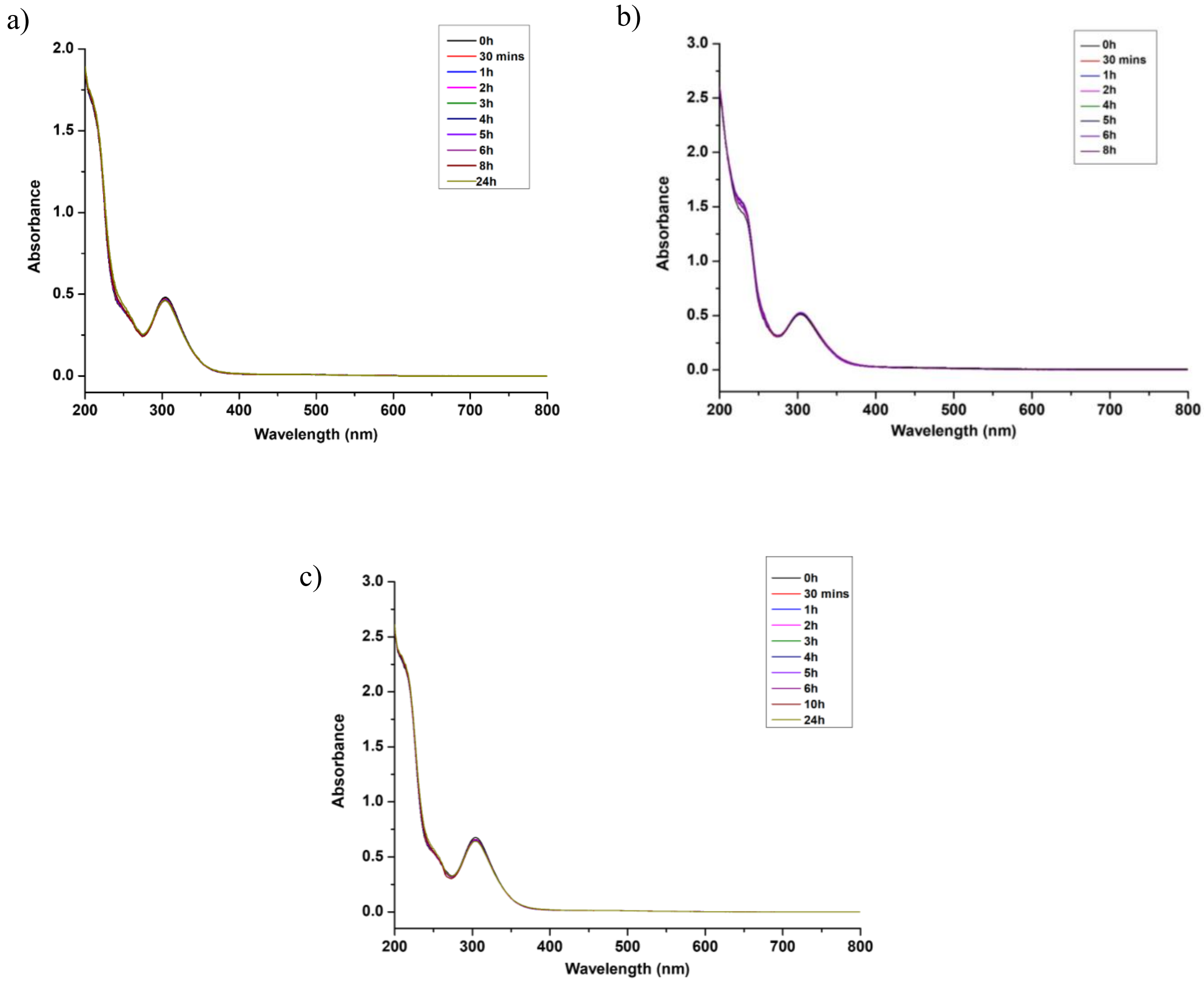

Figure S35. UV-vis spectra of a) **NaNH$_4$-C$_6$H$_4$AsMo$_6$** b) **NaNH$_4$-BrC$_6$H$_4$AsMo$_6$** and c) **NaNH$_4$-F$_3$COC$_6$H$_4$AsMo$_6$** in H_2O with 0.05 mM concentration. The time-dependent UV-vis spectra showed nice overlap, indicating stability in H_2O.

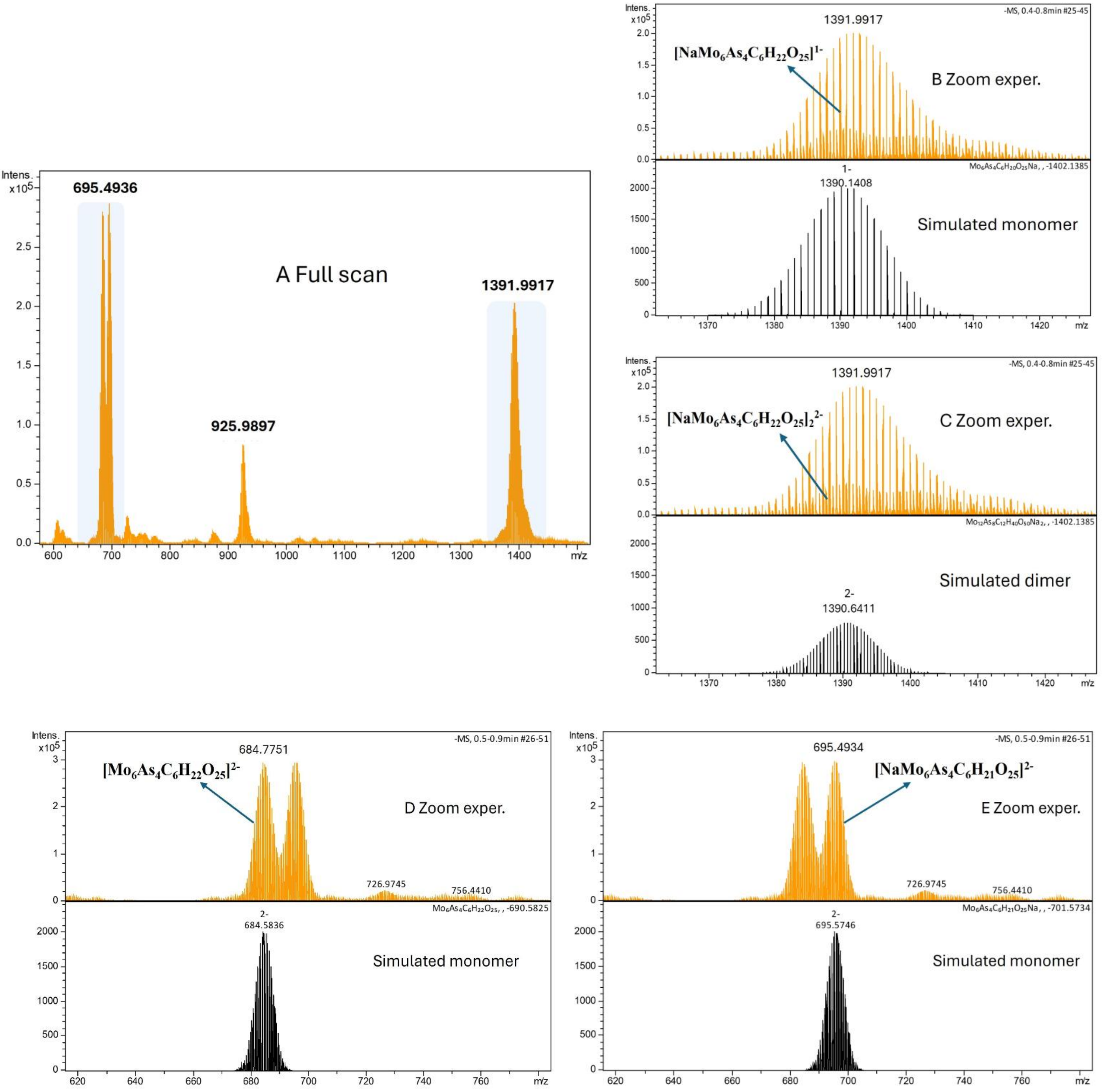

Figure S36. Negative mode ESI mass spectra of polyanion **As^{V}Mo$_6$**. **A:** Full scan full range view; **B:** expanded region around *m/z* 1391 with experimental spectra in upper panel (please note signals of dimeric species at lower intensity in between main signals) and simulated isotope pattern for the monomer in bottom panel; **C:** expanded region around *m/z* 1391 with experimental spectra in upper panel and simulated isotope pattern for the dimer in bottom panel; **D:** expanded region around *m/z* 684 with experimental spectra in upper panel and simulated isotope pattern for the monomer (H-adduct) in bottom panel; **E:** expanded region around *m/z* 695 with experimental spectra in upper panel and simulated isotope pattern for the monomer (Na-adduct) in bottom panel.

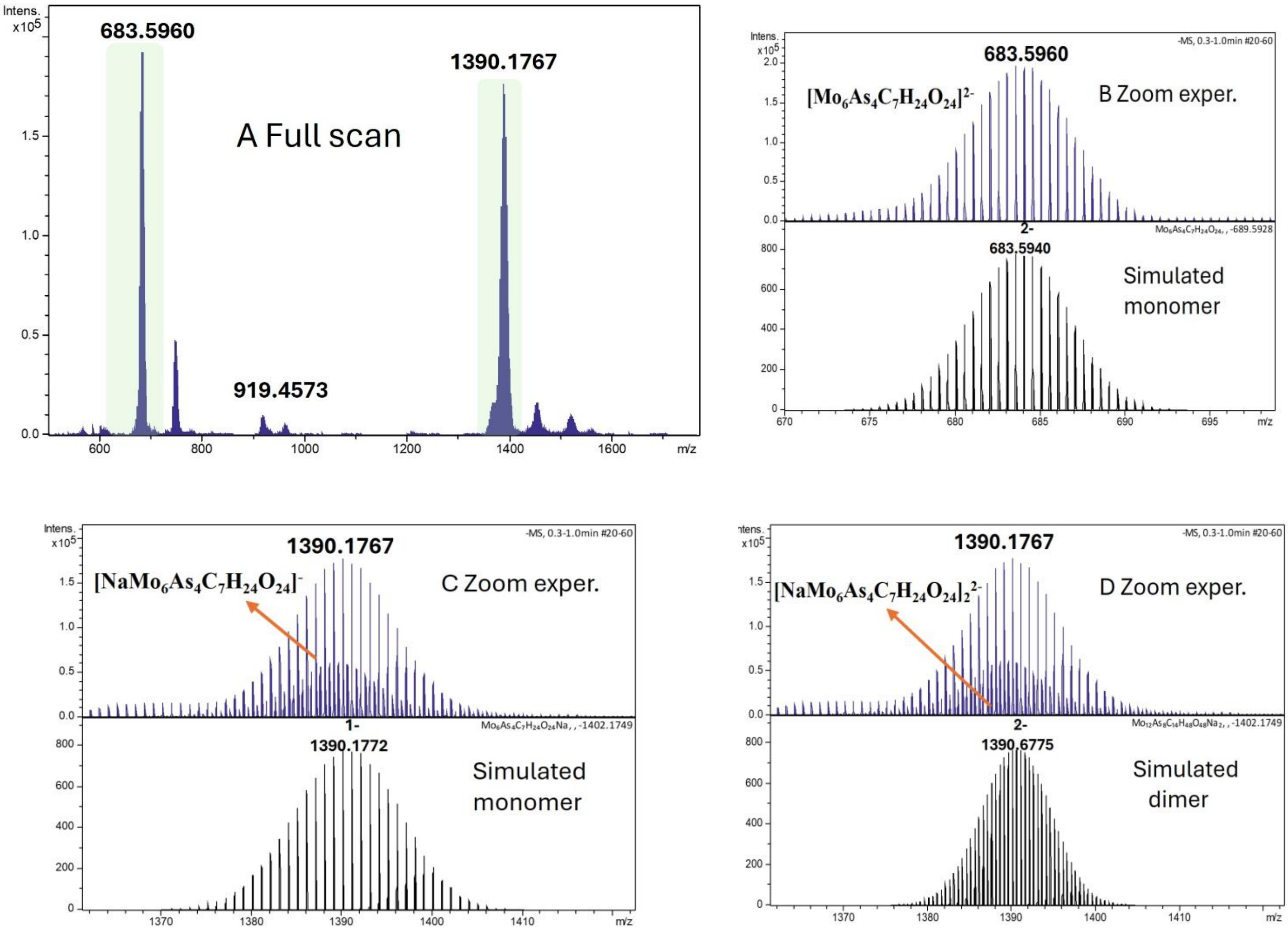

Figure S37. Negative mode ESI mass spectra of polyanion **CH_3AsMo_6**. **A:** Full scan full range view; **B:** expanded region around *m/z* 683 with experimental spectra in upper panel and simulated isotope pattern for the monomer in bottom panel; **C:** expanded region around *m/z* 1390 with experimental spectra in upper panel and simulated isotope pattern for the monomer in bottom panel; **D:** expanded region around *m/z* 1390 with experimental spectra in upper panel and simulated isotope pattern for the dimer in bottom panel.

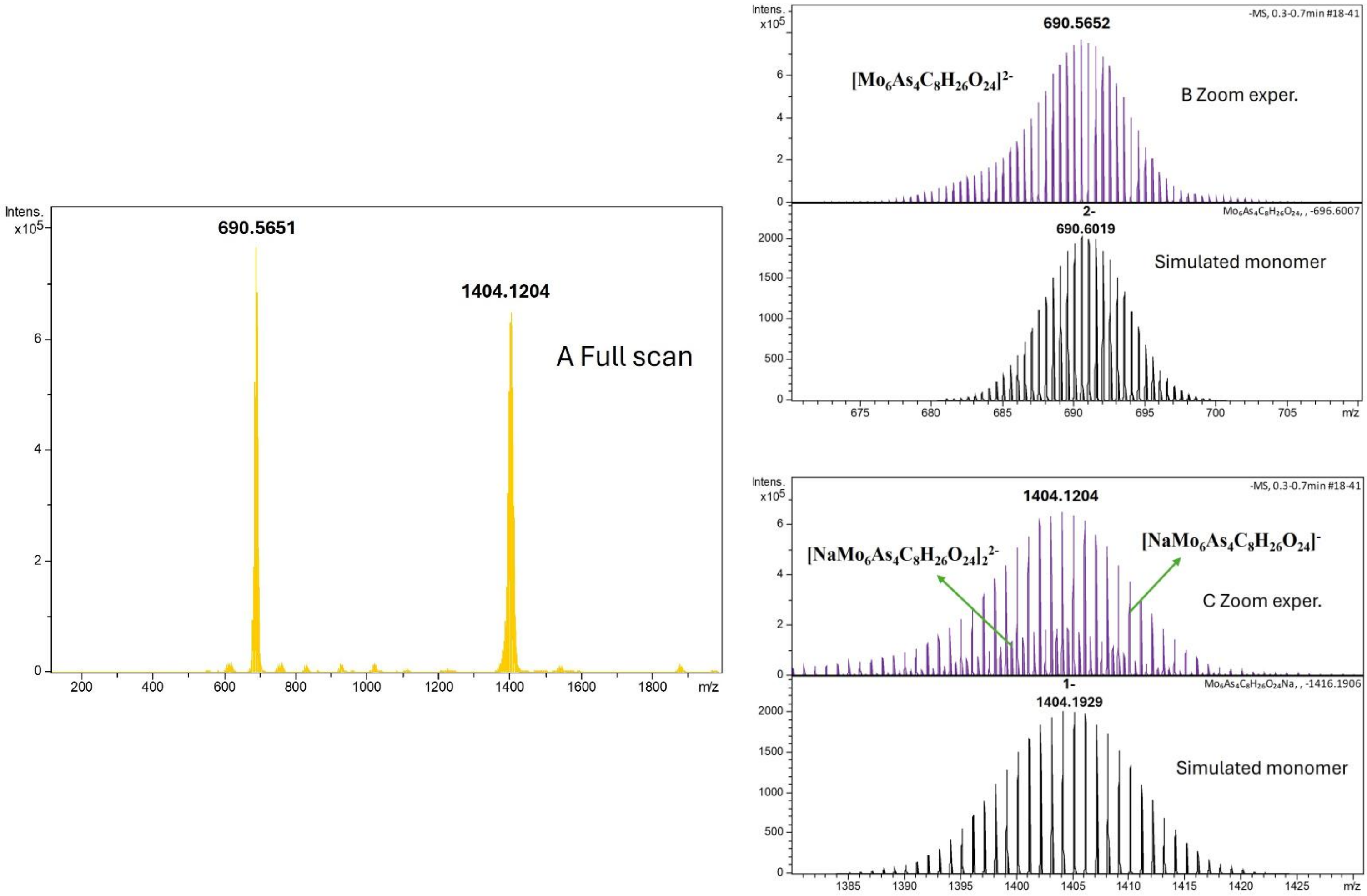

Figure S38. Negative mode ESI mass spectra of polyanion **$C_2H_5AsMo_6$**. **A:** Full scan full range view; **B:** expanded region around *m/z* 690 with experimental spectra in upper panel and simulated isotope pattern for the monomer in bottom panel; **C:** expanded region around *m/z* 1404 with experimental spectra in upper panel (please note signals of dimeric species at 0.5 *m/z* mass difference at lower intensity in between main signals) and simulated isotope pattern for the monomer in bottom panel.

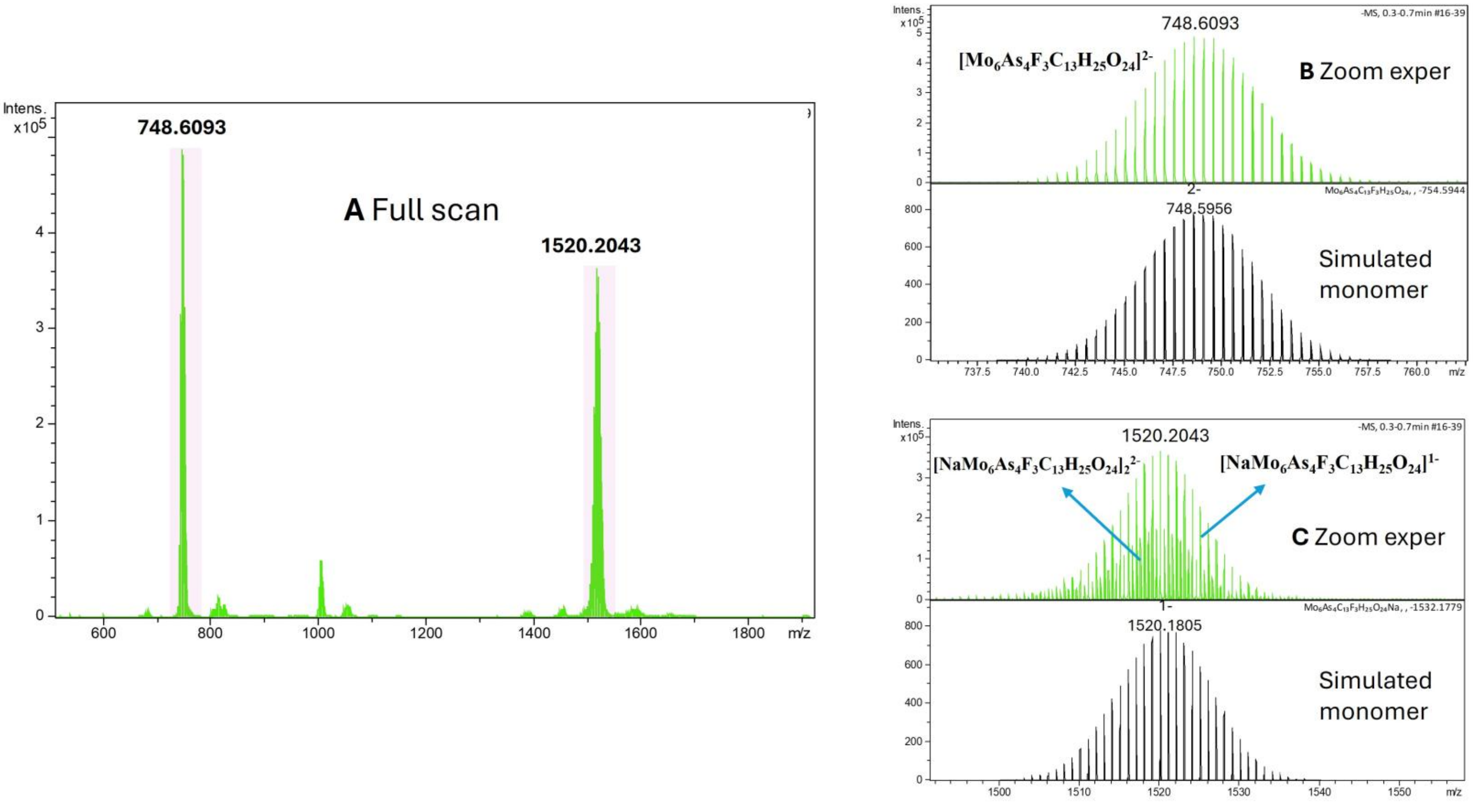

Figure S39. Negative mode ESI mass spectra of polyanion **$F_3CC_6H_4AsMo_6$**. **A:** Full scan full range view; **B:** expanded region around *m/z* 748 with experimental spectra in upper panel and simulated isotope pattern for the monomer in bottom panel; **C:** expanded region around *m/z* 1520 with experimental spectra in upper panel (please note signals of dimeric species at 0.5 *m/z* mass difference at lower intensity in between main signals) and simulated isotope pattern for the monomer in bottom panel.

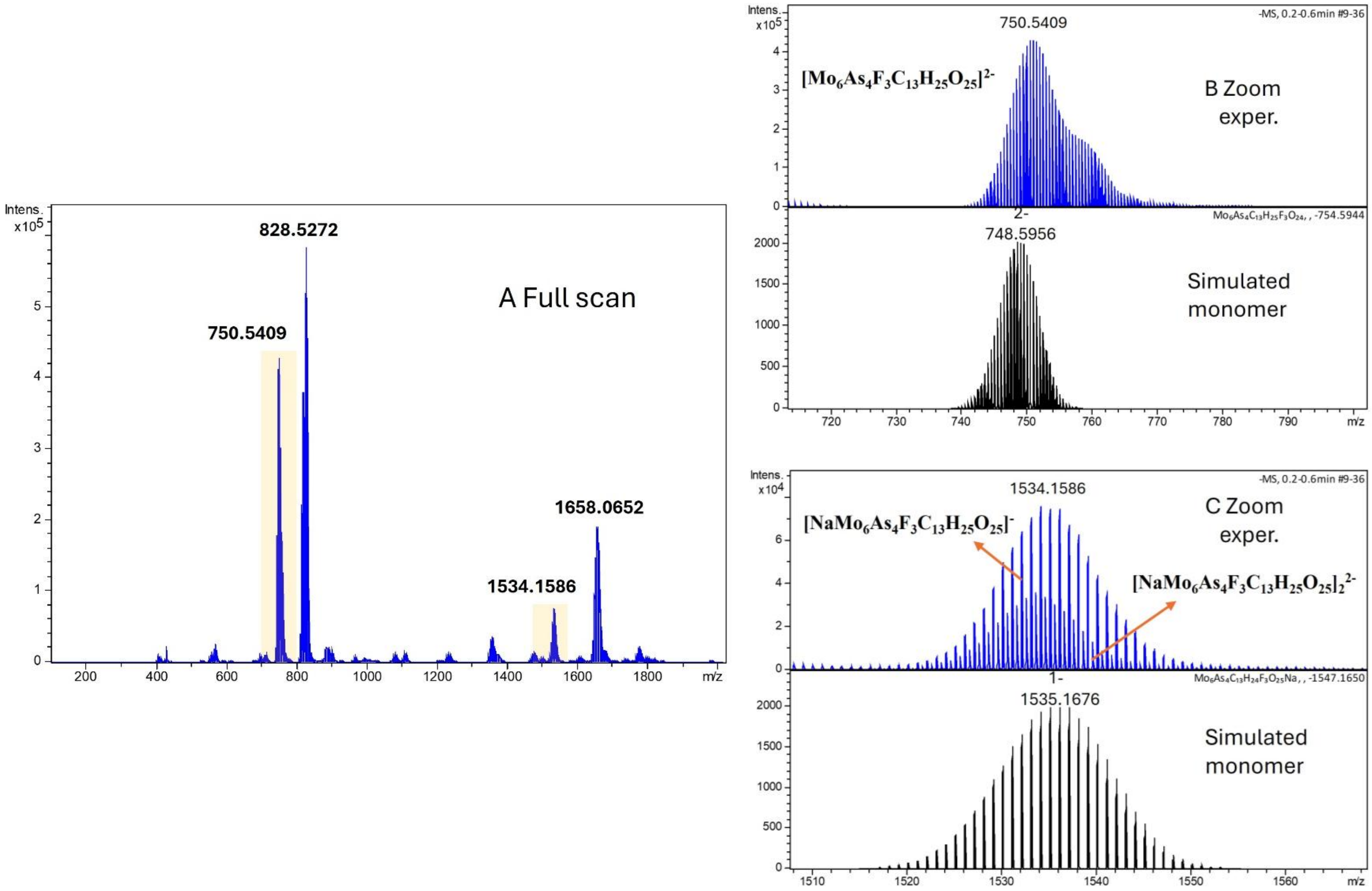

Figure S40. Negative mode ESI mass spectra of polyanion **$F_3COC_6H_4AsMo_6$**. **A:** Full scan full range view; **B:** expanded region around *m/z* 748 with experimental spectra in upper panel and simulated isotope pattern for the monomer in bottom panel; **C:** expanded region around *m/z* 1534 with experimental spectra in upper panel (please note signals of dimeric species at 0.5 *m/z* mass difference at lower intensity in between main signals) and simulated isotope pattern for the monomer in bottom panel.

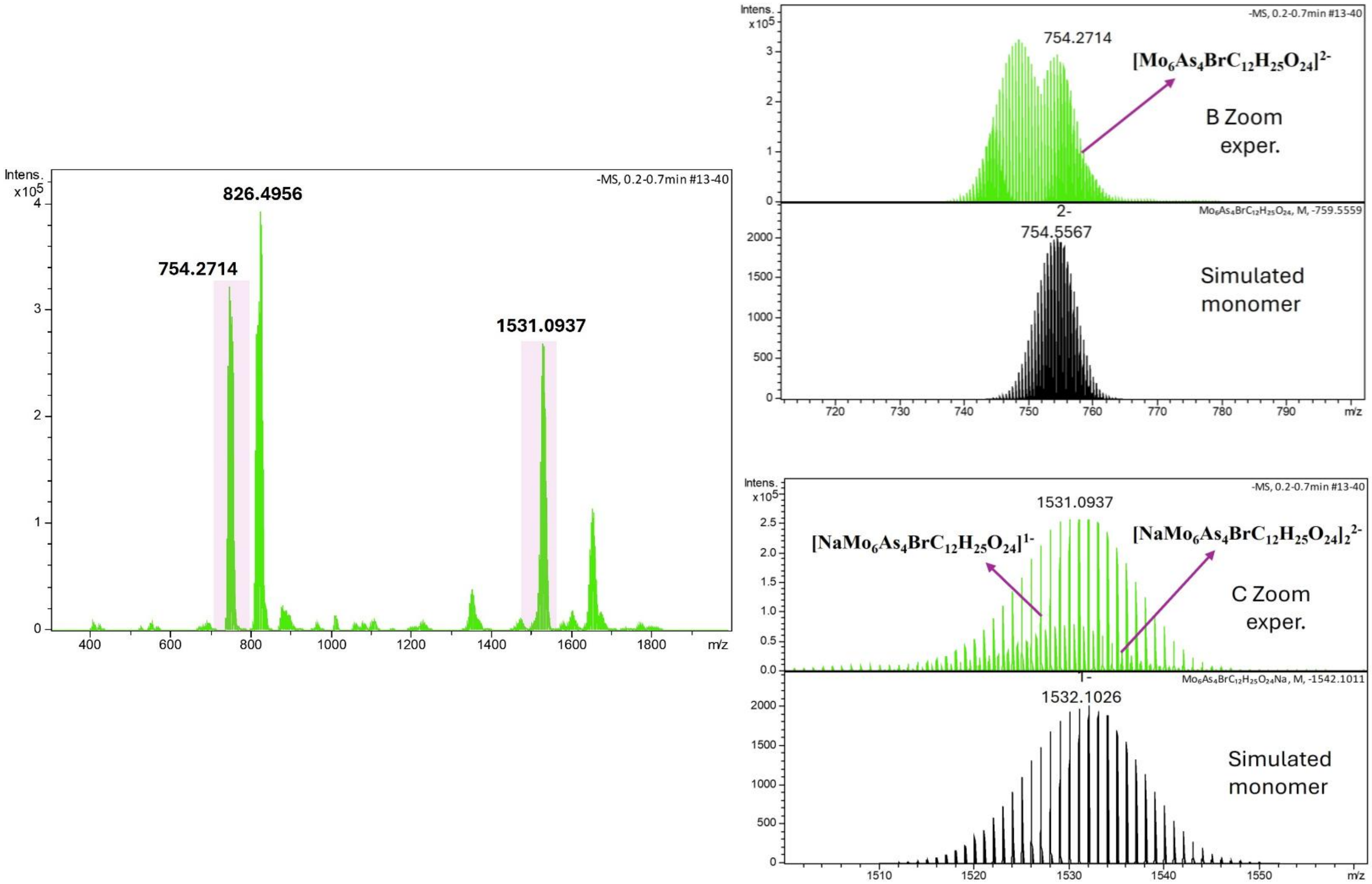

Figure S41. Negative mode ESI mass spectra of polyanion **$BrC_6H_4AsMo_6$**. **A:** Full scan full range view; **B:** expanded region around *m/z* 754 with experimental spectra in upper panel and simulated isotope pattern for the monomer in bottom panel; **C:** expanded region around *m/z* 1531 with experimental spectra in upper panel (please note signals of dimeric species at 0.5 *m/z* mass difference at lower intensity in between main signals) and simulated isotope pattern for the monomer in bottom panel.

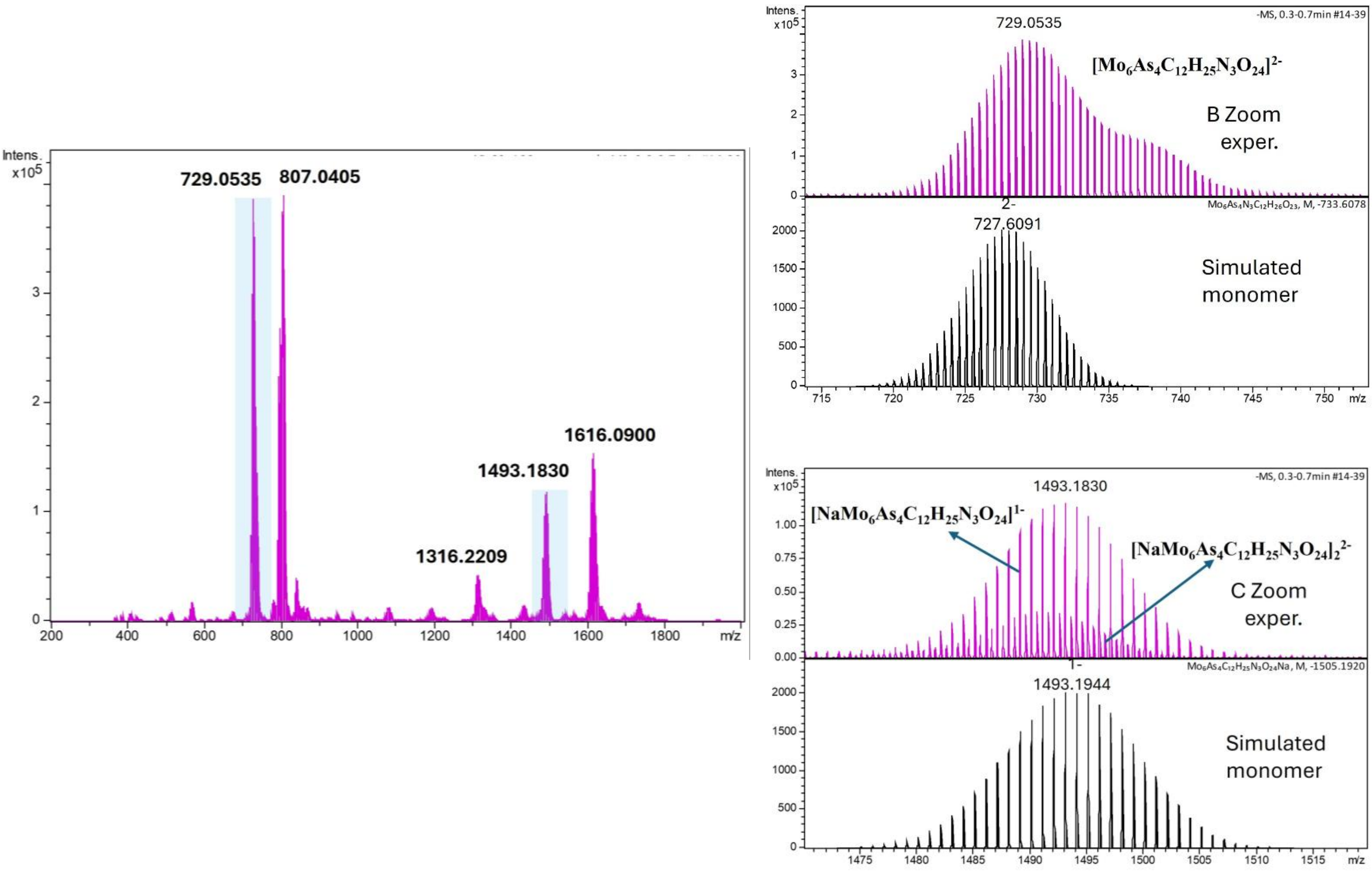

Figure S42. Negative mode ESI mass spectra of polyanion **$N_3C_6H_4AsMo_6$**. **A:** Full scan full range view; **B:** expanded region around *m/z* 729 with experimental spectra in upper panel and simulated isotope pattern for the monomer in bottom panel; **C:** expanded region around *m/z* 1493 with experimental spectra in upper panel (please note signals of dimeric species at 0.5 *m/z* mass difference at lower intensity in between main signals) and simulated isotope pattern for the monomer in bottom panel.

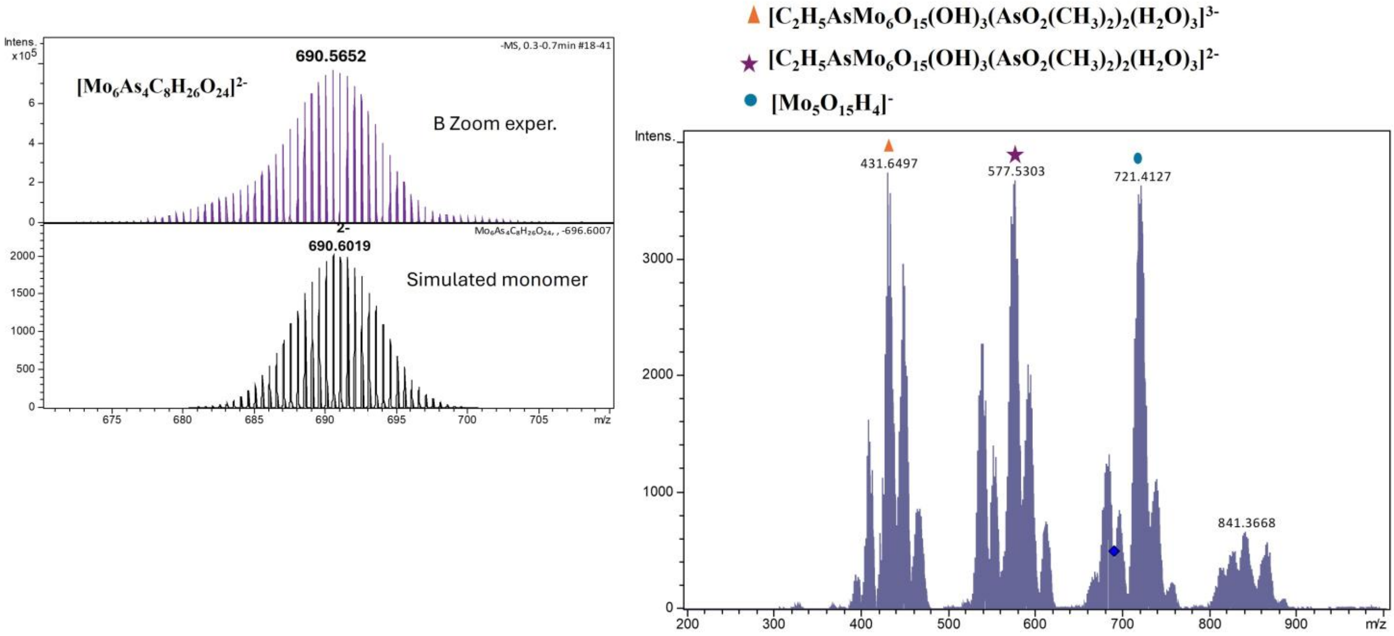

Figure S43. Tandem MS spectra in negative mode of **$C_2H_5AsMo_6$** with precursor ion at *m/z* 690. Right Panel shows experimental spectra of fragment ions centered around *m/z* 721, *m/z* 577 and *m/z* 431.

Agar diffusion test for *Listeria monocytogenes* MIC Plate for *Listeria monocytogenes*

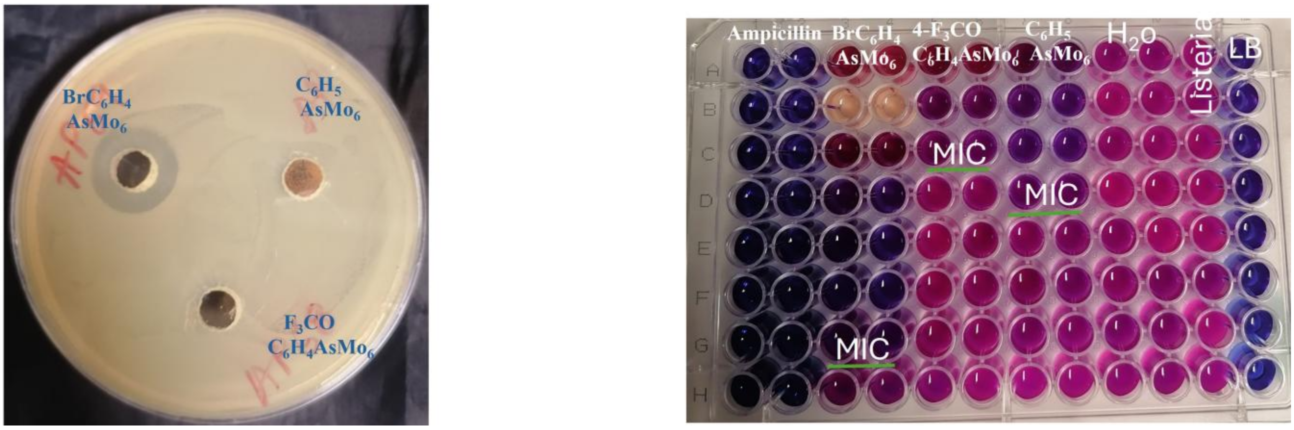

Figure S44. Agar diffusion test (left) showing the zone of inhibition and MIC plate (right) for *Listeria Monocytogenes*.
